# Deep active learning and knowledge transfer for rapid discovery of lithium metal battery electrolytes

**DOI:** 10.1038/s41467-026-70973-4

**Published:** 2026-03-27

**Authors:** Xufeng Hong, Xizhe Wang, Stephen J. Harris, Hongbo Zhao, Jiashen Meng, Qingshan Jia, Qianchuan Zhao, Kang Xu, Quanquan Pang, Benben Jiang

**Affiliations:** 1 Beijing Key Laboratory of Theory and Technology for Advanced Batteries Materials, School of Materials Science and Engineering, Peking University, Beijing, China; 2 CFINS, Department of Automation, Beijing National Research Center for Information Science and Technology, Tsinghua University, Beijing, China; 3 Lawrence Berkeley National Laboratory, Berkeley, CA USA; 4 Department of Chemical and Biological Engineering, Princeton University, Princeton, NJ USA; 5 SES AI Corp, Woburn, MA USA

**Keywords:** Batteries, Batteries, Computational science, Batteries

## Abstract

Designing electrolyte materials for high-energy lithium metal batteries requires navigating vast, discrete chemical spaces, where intricate interphasial and electrolyte chemistries render component interactions largely unclear. Traditional trial-and-error methods struggle with discontinuous electrolyte-performance relationships and inefficient adaptation to new molecular candidates, hindering discovery. Here, we propose a two-stage deep active learning framework with knowledge transfer for rapid electrolyte design. In stage one, deep active learning with deep kernel learning selects informative experiments and models discontinuous relationships between formulation and performance, improving sample efficiency and reducing experimental cost. In stage two, target statistic coding quantifies what was learned and transfers it to new design settings, such as expanded formulation spaces and newly introduced components, using only a small number of additional measurements. Using this framework, we found electrolytes that increase the average lifetime of lithium metal symmetric cells by threefold after three learning iterations, and we rapidly identified improved formulations for Li^0^ | |LiNi_0.8_Co_0.1_Mn_0.1_O_2_ full cells in expanded chemical spaces. This work provides an experiment-driven, sample-efficient route to explore complex electrolyte formulation spaces and quantify inter-component correlations, as well as a realistic, high-cost, small-data benchmark for probabilistic surrogate modeling and sequential decision-making in discrete chemical spaces.

## Abstract (web summary)

Developing efficient battery electrolytes is a slow and complex challenge. Here, authors create a deep active learning framework that rapidly identifies high-performance materials and transfers knowledge to new designs, tripling battery lifespan while requiring significantly fewer experiments.

## Introduction

Deconvoluting complex systems is central to materials science, chemistry and physics^1–4^. Key descriptors include the basic components, their properties, and their correlations, which must be decoded for rational design^4,5^. However, when multiple independent components are introduced, correlations grow exponentially, making hidden relationships difficult or impossible to identify analytically^4–6^. To circumvent such complexity, researchers often seek unified theories, such as the structure-property-performance relationships in materials science, which was conventionally derived through extensive trial-and-error experiments^6–9^. Notably, many materials systems exhibit marked “discontinuity”: changing a single chemical component can induce a sudden rather than incremental change in system behaviour, even when all other variables are kept identical^9,10^. In the absence of accurate knowledge of the correlations among those components, building unified theories demands prohibitively costly and time-consuming data acquisition^11^. In parallel, statistical and machine learning (ML) approaches have emerged as useful tools to extract hidden relationships from data without requiring full atomic-level understanding^2,4,12–14^. In such methods, purely data-driven models offer flexibility but often lack interpretability^8,15–17^, whereas knowledge-driven approaches leverage empirical rules but can fall short in rigour when applied to large, complex systems^6,15,18^.

Lithium metal batteries (LMBs) using metallic lithium negative electrodes (Li^0^) are promising next-generation batteries because Li^0^ possesses the lowest redox potential (−3.04 V *vs*. standard hydrogen electrode) and high specific capacities (3860 mA h g^−1^), the combination of which promises the highest possible specific energy^9,19–21^. However, the same low redox potential also leads to high reactivity and irreversibility, causing dendrite formation, dead Li^0^ and capacity loss through continuous Li^0^-electrolyte reactions^3,22,23^. For decades, stabilizing Li^0^ has been hampered by the lack of electrolytes that can form chemically, electrochemically, and mechanically stable interfaces^24–27^. Nevertheless, the pursuit of LMB motivates researchers to generate and verify new electrolyte formulas in the vast design space that includes solvent molecules, additives and salt anions of various structures, as well as their relative ratios^27–30^. Buried relationships between these variables and Li^0^ reversibility are generally attributed to solvation structures, solid-electrolyte interphase (SEI) chemistry and morphology, interfacial ion and mass transport, and charge-transfer processes^31^. However, owing to the complexity of Li^0^-electrolyte interactions, a unified mechanistic description remains elusive^32–35^. Unlike the situation in particle reaction kinetics, high-entropy alloys or carbon-nitrogen coupling reactions^12,14,36^, it has so far been impractical to incorporate generalized physical and chemical principles into predictive electrolyte design for LMBs.

Material discovery for LMBs requires substantial resources and time, as experiments are labour-intensive and often test only a single variable^3^. Verifying reversibility and stability demands long-term cycling, while promising candidates consume even more time and resources. In contrast, failed or negative results, which would be especially valuable for ML, are frequently neglected, leading to wasted or misdirected efforts^7,8,37,38^. The challenge is compounded by large experimental variability, particularly in electrolyte design, where results are often incomparable across studies due to different cell configurations and testing conditions^38–40^. To complicate it further, unlike the optimization of nonchemical (particularly numerical) subjects of interest—the examples of which include battery fast-charging protocols^16,18,41^ or tuning electrolyte physical properties^42^ like solubility, conductivity, and viscosity, which vary continuously—electrolyte design faces a highly discrete search space. This arises because the relationship between components and performance is sensitive to small changes, owing to complex equilibrium and non-equilibrium processes within the cell, especially at the interfaces. As a result, even a single component change can qualitatively alter the underlying chemical interactions, leading to a combinatorial explosion of the design space^43^. Such discontinuities remain beyond precise chemical intuition and must otherwise be identified through painstaking trial-and-error exploration^6,44^.

Pioneering ML-assisted approaches, often coupled with computational screening or robotic experimentation, have shown promise in electrolyte design^11,32,42,45^. However, these efforts have largely focused on numerically continuous parameter spaces^42,45^. Learning methods capable of handling large parameter-performance discontinuities, critical for industrially relevant spaces, remain underdeveloped. Some studies have relied on theory-driven descriptors such as the lowest-unoccupied-molecular-orbital or highest-occupied-molecular-orbital (LUMO/HOMO) theory, ionic conductivity, or atomic ratios, etc.^32,45^; but these are retrospective and capture only known principles rather than uncovering new rules^32^. Furthermore, exploration was typically restricted to a predefined small parameter space, restricting knowledge transfer to broader chemical domains^8,14^. Given the importance of deriving new knowledge directly from experimental data, it is essential to develop advanced ML methods that can effectively explore discontinuous chemical spaces while revealing novel correlation patterns.

**Fig. 1:**
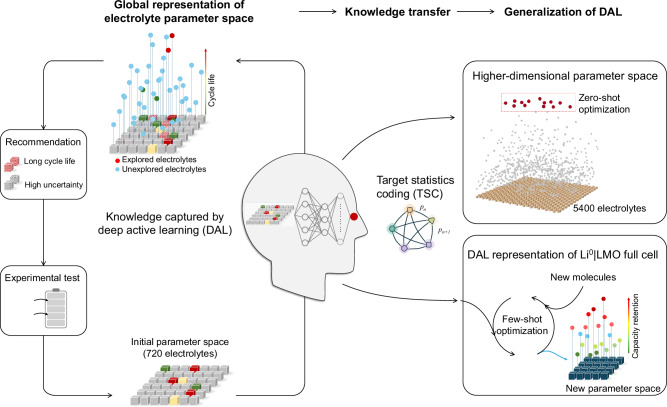
Two-stage framework for rapid discovery of lithium metal battery electrolyte enabled by deep active learning. In the first step, DAL iterative optimization is performed in a relatively small parameter space consisting of 720 electrolytes, which not only quickly identifies high-performance electrolytes through iterations, but importantly serves to capture the electrolyte design knowledge (left panel). After three optimization iterations, DAL achieves a well-represented model of the full parameter space, which then transfers the knowledge through a proposed TSC approach for new-task optimizations: optimizing electrolytes (1) in a higher-dimensional parameter space (with zero-shot optimization, top right panel) and (2) Li^0^||LMO full cells, including exploration of a new parameter space incorporating new molecules from recent literature (few-shot optimization, bottom right panel).

To address these challenges, we developed a two-stage optimization framework that leverages deep active learning (DAL) and knowledge transfer for rapid electrolyte optimization (Fig. 1). The first stage employs DAL, which integrates deep kernel learning (DKL) with Thompson sampling (TS) to effectively model highly discontinuous component-performance correlations. This approach, based on DKL’s capability for global representation of the electrolyte parameter space, enables efficient exploration of vast chemical spaces with minimal experimental cost and without reliance on empirical knowledge. Subsequently, a target statistic coding (TSC) scheme is used to quantify correlations among components based on both positive and negative outcomes accumulated during the DAL process. This quantified information, comprising substantial new knowledge, serves as the foundation for the second stage of our framework. The second stage involves transferring this accumulated knowledge into DAL optimization processes targeting substantially larger parameter spaces, thereby facilitating identification of high-performance electrolytes beyond the original parameter space. To validate this stepwise DAL workflow, we demonstrate successful knowledge transfer from the optimization of Li^0^||Li^0^ symmetric cells to optimization of strictly parameterized high-energy Li metal full cells, as well as to expanded parameter spaces incorporating newly reported molecules from recent literature. From the perspective of battery research, this framework organizes costly cycling experiments into a structured, sample-efficient exploration of electrolyte formulations; from the perspective of ML, it provides a realistic, high-cost and small-data testbed for probabilistic surrogate modelling and sequential decision-making in discrete chemical spaces.

As an example, we construct a large discrete parameter space within a fixed discrete component library containing 720 electrolyte formulas consisting of diversified solvents, lithium salts, salt concentrations and additives and then optimize with limited experimental battery testing resources (32 battery testing channels). Given the high discontinuity of the electrolyte formulas and a large experimental measurement noise^39,40^, a probabilistic surrogate model of DKL with highly expressive and scalable power of kernel functions is utilized to model the abrupt changes in the performances across the chemical space of Li^0^ electrolytes. An acquisition function of TS is then constructed to efficiently probe the parameter space of electrolyte formulas by balancing exploration and exploitation. After three DAL recommendation-experimentation iterations, that is, with a total testing expense of 4.4% of the testing resources if the full parameter space is explored with high confidence (detailed discussions in “Methods”), optimized electrolyte formulas that enable Li^0^||Li^0^ symmetric cells to show a three-times longer cycling life than those with the state-of-the-art electrolytes can be identified (left panel in Fig. 1). Moreover, with DAL’s global representation of the parameter space, we statistically decode the correlations among components based on the DAL-recommended electrolyte formulas, which reveals not only prior knowledge reported in literature but importantly generates new knowledge of correlation patterns. Based on the revealed correlations, we can zero-shot identify four “complex” electrolytes—composed of the preset chemicals but formulated beyond the preset parameter space—which result in longer cycling life than any of the originally explored electrolytes (top right panel in Fig. 1). With the Li^0^||Li^0^ symmetric cell optimization as the basis of learning for knowledge transfer, we further optimize the electrolytes with regard to the Li^0^||LMO full cells in new parameter space containing newly added molecules (LMO, layered metal oxide; bottom right panel in Fig. 1). By establishing an intercomponent correlation network, the proposed framework can be applied on the exploration of other materials systems that have previously been deemed too complex to study by exhaustive testing due to the lack of theoretical guidance.

## Results

**Fig. 2:**
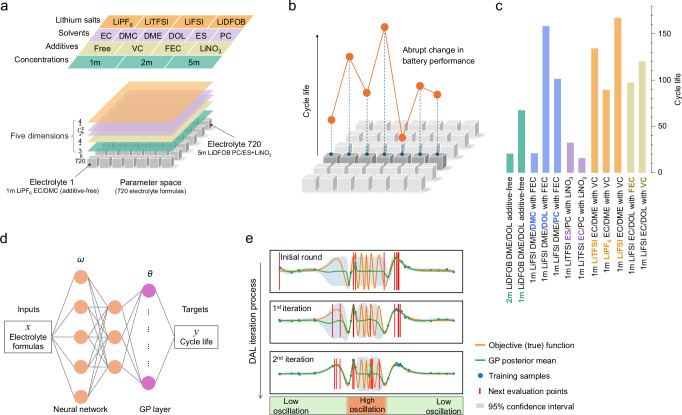
The electrolyte design problem formula and proposed DAL scheme for electrolyte optimization featuring large discontinuities. **a** The illustrative formula of the electrolyte parameter space to be explored; each electrolyte is designed to contain a specific concentration of one lithium salt, with two solvents, one or no additive. This results a five-dimensional space consisting of 720 electrolyte formulas. Abbreviations for components in the diagram can be found in the “Methods”. **b** The illustrated discontinuity between electrolyte formulas and cycle lifespan in a discrete chemical space within a fixed discrete component library. **c** Experimental evidence showing the abrupt change in Li metal battery cycling life without any tractable. pattern as a single component is varied while others remain the same (changes are highlighted in the colored). **d** The illustrative networking structure of the DKL model. A feed forward neural network *g* parametrized by ω projects the input data ***x*** into the latent space, denoted as *g*(***x***|*w*), which is followed by using a standard GP kernel; ${{{\boldsymbol{\theta }}}}$ is the hyperparameter of the base kernel. **e** The numerical simulation of a DAL exploration process on an exemplary objective (true) function with rapid oscillations (i.e., discontinuity), namely, $f=(1-\left|x\right|)\sin (20{(1-\left|x\right|)}^{6})$, where the search space of *x* is defined as [−1,1], and the initial round is obtained through random search. This one-dimensional function can simulate the learning and exploring ability of the DKL-embedded active learning method for optimizing an objective function with large discontinuity.

In this work, the key components (lithium salts, solvents, additives and their concentrations) were selected to form a parameter space for electrolyte design (detailed in Supplementary Note 1), which includes four Li salts (lithium hexafluorophosphate (LiPF_6_), lithium bis(trifluoromethanesulfonyl)imide (LiTFSI), lithium bis(fluorosulfonyl)imide (LiFSI), and lithium difluoro(oxalato)borate (LiDFOB)), six solvent molecules (ethylene carbonate (EC), dimethyl carbonate (DMC), 1,2-dimethoxyethane (DME), 1,3-dioxolane (DOL), ethylene sulfite (ES), and propylene carbonate (PC)) and three additive molecules (vinylene carbonate (VC) and fluoroethylene carbonate (FEC), and lithium nitrate (LiNO_3_), while Li salt concentration is added as an independent component (that is, 1, 2, 5 m in molality). Together, the above components generate a parameter space with 720 electrolyte formulas by applying constraints of each electrolyte having a single Li salt, two solvent molecules with a 1:1 volume ratio, and a single additive molecule (0 or 5 wt%) (each shown as a square in the gray matrix in Fig. 2a). The detailed electrolyte formulas are indexed in Supplementary Data 1, and for clarity, the denotations of the chemical components are not described in detail here.

Our objective is to determine the optimal electrolyte formulas with long cycle life in a sample-efficient manner (i.e., as few experiments as possible). The major challenges in such an electrolyte optimization problem are (i) the large space of possible electrolyte formulas, while the experimental budget is often limited; (ii) the chemical space for electrolyte optimization is discrete, and its component-performance relationship is largely discontinuous, where an abrupt change in the performance often occurs with the change of a single chemical or element, as illustrated in Fig. 2b; and (iii) the experimental output of the electrolytes is noisy due to the large experimental or measurement variability associated with Li^0^.

The performance of these electrolytes was evaluated by the cycle life of a Li^0^||Li^0^ symmetric cell containing such electrolytes, which was conducted using a protocol of an areal capacity of 2 mA h cm^−2^ and a current of 0.5 mA cm^−2^ (Supplementary Fig. 1). The cell lifespan is defined as the number of cycles when the cell ends with a hard short circuit, a soft circuit, or an overpotential greater than 0.5 V (Supplementary Fig. 1). A statistical analysis is conducted on the variations among the electrolyte’s components and lifespan within the parameter space, which underscores that even minor adjustments to a single component can induce significant change in cell lifespan (Supplementary Fig. 2), thus confirming the discontinuity nature of the cell performance as a function of electrolyte component resulting from the complex interplay among these components (Fig. 2c).

Active learning (AL) is widely used in many fields^46–48^, for example, to optimize the fast-charging protocols of lithium-ion batteries under the constraints of battery voltage and temperature^49,50^. The objective function of the fast-charging optimization problem is usually smooth; that is, for two similar charging current configurations, their corresponding outputs of the cycle life are close. However, for the black-box functions with rapid oscillations, which are characteristic of the objective function in the electrolyte optimization problem, the representation ability of the commonly-used surrogate model of the Gaussian process is limited because the covariance function used by the Gaussian process is a stationary covariance function, namely, *κ*(*x*, *x*′) = *κ*((*x* − *x*′), 0), where *κ* and *x*, *x*′ stand for the covariance function and the input variables, respectively. For rapidly oscillating objective functions, simply measuring the similarity of the output by the distance between the input variables makes the surrogate model of AL highly inaccurate. In addition, the selection of the covariance function depends on prior knowledge of the optimization objective, which is usually not available.

To address these issues, we propose a DKL-based Gaussian process approach to model the complex structure-property relationships of the chemical space for LMB electrolytes (Fig. 2d). DKL combines the representational power of deep neural networks with the reliable uncertainty estimates of Gaussian processes, which provides a method to automatically determine covariance functions. On one hand, the deep neural network can learn structure-property relationships directly from data, automatically forming adaptive kernel representations and thereby avoiding the need for manually defined covariance kernels with predefined smoothness assumptions, which enhances the representation ability of models. On the other, the nonparametric model automatically calibrates its complexity through the marginal likelihood function of Gaussian processes, making the model resistant to overfitting.

Given a dataset with the variables ***x*** and ***y*** that represent the electrolyte formulas and cell cycle life, respectively, the inputs can be converted by the DKL as^51^:

1 $${k}_{{{{\rm{DKL}}}}}\left({{{{\boldsymbol{x}}}}}^{\left(i\right)},{{{{\boldsymbol{x}}}}}^{\left(j\right)} | {{{\boldsymbol{\omega }}}},{{{\boldsymbol{\theta }}}}\right)={k}_{{{{\rm{base}}}}}\left(g\left({{{{\boldsymbol{x}}}}}^{\left(i\right)}|{{{\boldsymbol{\omega }}}}\right),g\left({{{{\boldsymbol{x}}}}}^{\left(j\right)} | {{{\boldsymbol{\omega }}}}\right) | {{{\boldsymbol{\theta }}}}\right)$$

where ${k}_{{{{\rm{base}}}}}\left({{{{\boldsymbol{x}}}}}^{\left(i\right)},{{{{\boldsymbol{x}}}}}^{\left(j\right)}\right)$ denotes the base kernel function, ${{{\boldsymbol{\theta }}}}$ is the hyperparameter of the base kernel, and $g$ is the nonlinear mapping learned by the deep neural network, which is parameterized by the weights ***ω****.* The representational capability of deep neural network-based kernels in DKL largely surpasses that of conventional kernels, such as radial basis functions (RBF) employed in standard Gaussian processes. This enhanced capability is particularly valuable when modeling the objective functions of electrolyte optimization problems, where the deep kernel excels at capturing complex and discontinuous relationships inherent in the chemical parameter space (see “Methods” for more details), facilitating a substantially more efficient optimization for this challenging problem.

By maximizing the marginal likelihood of DKL, we learn all deep kernel hyperparameters, including the parameter ${{{\boldsymbol{\omega }}}}$ of the deep neural network and the parameter ${{{\boldsymbol{\theta }}}}$ of the base kernel. The covariance function in (1) can be adaptively learned from the data by utilization of the flexible representational capability of deep neural networks with the reliable uncertainty quantification provided by Gaussian processes. To fully leverage the experimental throughput of parallel evaluations, an acquisition function of TS (see “Methods”) is then constructed based on the posterior distribution from the surrogate model of DKL. This strategy enables scalable batch-parallel optimization and efficiently sample the parameter space of electrolyte formulas by balancing exploration (i.e., testing regions with high uncertainty) and exploitation (i.e., testing promising regions based on completed experiment results)^52^.

As illustrated in Supplementary Fig. 2, the space of electrolyte formulas is discrete in the dimensions of Li salts, solvents and additives, but somewhat relatively continuous in salt concentrations (Supplementary Fig. 2d–f), which points to an objective function featuring both discontinuous transitions and continuous variations. This sharp and non-monotonic response of cycle life to single component changes demonstrates the “rapid oscillations” in the objective function that characterize the electrolyte optimization problem. Therefore, a numerical function of $f=\left(1-\left|x\right|\right)\sin \left(20{\left(1-\left|x\right|\right)}^{6}\right)$ was set up to simulate the process to verify the efficacy of the DAL approach based on the surrogate model of DKL (Fig. 2e and detailed in “Methods”). Notably, the DAL approach can adaptively balance the exploration and exploitation processes over the iterations on both high and low oscillation regions (Fig. 2e). Compared with the standard Gaussian process-based active learning (GP-AL), our DAL approach can build a more accurate surrogate model for such a rapidly oscillating objective function (Fig. 2e and Supplementary Fig. 3), as further validated by experimental results (Supplementary Fig. 3e). Even when the training samples are deliberately assigned with large noise, generally arising from minor differences and large errors in lithium metal negative electrode in experiments, the DAL approach efficiently identifies the optimal solution as early as the second iteration (Supplementary Fig. 4). Concurrently, as the iterations progress, DAL continues to refine potential candidate formula points. After three iterations, the mean square error produced by the DAL approach is 42.2% lower than that by GP-AL, and the optimal solution obtained by DAL outperforms GP-AL by 9.9% (discussed in detail in Supplementary Figs. 3 and 4).

As we observed experimentally, electrolytes with longer cell lifespans exhibit a rather scattered distribution due to experimental variability, whereas electrolytes with shorter lifespans occur with a more concentrated distribution (Supplementary Fig. 4). We also assessed different noise levels during numerical simulations, and observed that the DAL approach possesses good and robust performance for diverse noise levels achieving optimal values of *x* = 0.94 at 5%, 10%, and 20% noise levels (Supplementary Fig. 4). This robustness to noise interference is higher than that of the GP-AL method. The DAL approach showed a marginal 0.4% reduction in performance compared to an 11% decrease in the GP-AL method under a 10% noise level (detailed in Supplementary Note 2).

**Fig. 3:**
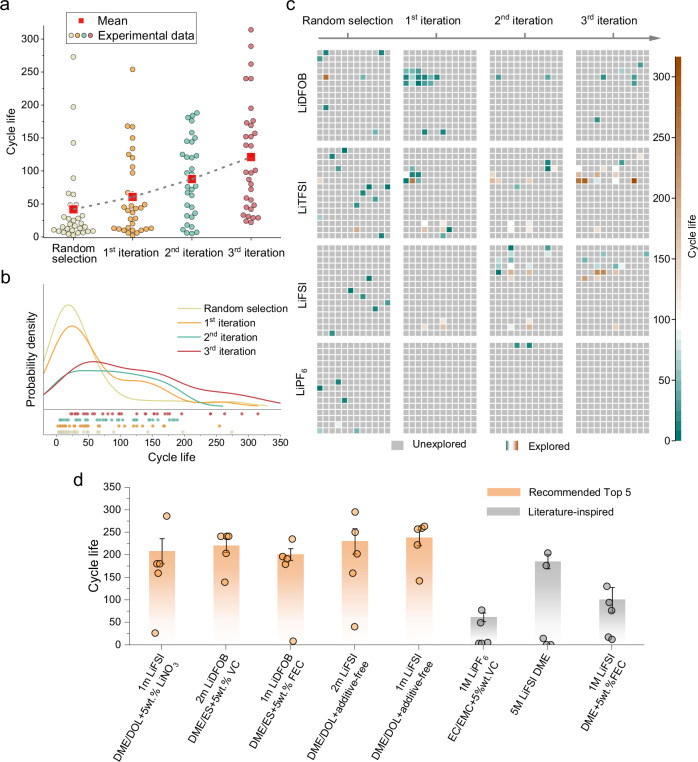
The electrolyte optimization results with three DAL iterations. **a**, **b** Statistical representations of the cycle life of experimentally tested Li metal batteries at 25 °C (**a**) and their probability density distribution (**b**) as the iteration progress. The colored dots in (**a**, **b**) represent the specific data points of experimental tests. The red boxes in (**a**) are the mean lifespan in each batch. **c** The visualization of the DAL iterations showing the evolution of distribution of tested electrolytes and its battery cycle life (color coded) as iteration progress, which is herein representatively grouped by the type of lithium salts for easy visualization. **d** Experimental validation of the top 5 optimized electrolytes ranked by DAL, which is compared to the high-performance electrolytes reported in the literature using similar components. *n* = 5 independent cells per electrolyte formulation. The length of each bar represents the mean cycle life from repeated measurements with standard deviation.

To demonstrate the overall success of our DAL approach, we ran three DAL iterations with a total number of 128 cells tested, including the first round of random selection (see detailed electrolyte formulas in Supplementary Tables 1–4). Figure 3a clearly shows that as DAL iterates, the mean lifespan of the tested cells experiences a notable increase. Specifically, the mean cycle lives are 41.9, 60.7, 87.7, and 125.1 cycles for the random round and subsequent three iterations, respectively. In addition, the percentage of cells with a short cycle life (defined for <50 cycles in this work) shows a significant decrease over iterations, from 80.6% for the initial random round to 28.1% after the third iteration, whereas the percentage of cells with a long cycle life (defined for >100 cycles) increases from 9.7% to 40.6% after the third iteration (Fig. 3b). This clearly shows the efficacy of our DAL approach for electrolyte exploration, even with the large measurement noise associated with Li^0^ experiments. As the iterations progress, DAL’s confidence in optimizing the long-life electrolyte increases as confirmed by statistical ranking of tested cells’ cycle life, and after three iterations, the explored long-life electrolytes sufficiently represent the parameter space (discussed in Supplementary Fig. 5).

We now further show the success of DAL from a microscopic scale. Figure 3c shows the evolution of the space that is grouped by the type of salts (as a representative). The electrolyte formulas for the first round are randomly selected with two replicate tests (first column in Fig. 3c) to generate an initial dataset for DAL model building. Through only one DAL iteration, some electrolytes (i.e., those containing LiTFSI and LiFSI) are identified as “potential candidates” (second column in Fig. 3c). Interestingly, the LiDFOB regions (red dot area, first column in Fig. 3c) were allocated with many resources for the first iteration, but much fewer after that, which indicates the high experimental uncertainty in this zone that requires careful validation. By learning and exploiting structural information of the parameter space, DAL avoids evaluating electrolytes with low estimated cell cycle life and directs more resources to the high-performance region. This is also confirmed by individually analyzing other components of the space (discussed in Supplementary Fig. 6). In all, 621 of 720 electrolyte formulas were never tested (Fig. 3c); that is, we spent 0.22 experiments per electrolyte formula on average (utilizing only 4.4% of resources if one assumes exhaustive testing of five parallel cells for each electrolyte). Note that the DAL repeatedly tested several formulas with high estimated cycle life to decrease uncertainties due to a large experimental measurement variability (e.g., top regions of the LiTFSI and LiFSI groups, Fig. 3c).

In retrospect, the optimization results revealed some overall knowledge patterns; for example, electrolytes containing LiPF_6_, LiDFOB, DMC, and with a high concentration of 5 m salts were not favored in general, indicating a negative outcome and leading to their exclusion from further iterations (Fig. 3, Supplementary Fig. 6, and Supplementary Note 3). Indeed, as the literature has previously reported, electrolytes containing LiPF_6_ and DMC do not support stable operation of Li^0^ due to the persistent formation of SEI^53–55^; this crucial information was apparently quickly captured by DAL.

We further validated the high efficacy of DAL by experimentally testing a subset of five electrolyte formulas identified with the highest cell lifespan recommended by DAL after the third iteration. We tested each of these electrolytes five times by cycling the cells until failure. The mean cycle life, calculated as a weighted average from these multiple tests, is considered an estimate of the true cycle life (Supplementary Note 4). The recommended top five electrolytes, all containing ether components, exhibit a longer lifespan with high reproducibility compared to the state-of-the-art electrolytes with similar components (Fig. 3d, the selection criterion in Supplementary Note 5)^54^, verifying the efficacy of the DAL approach for the electrolyte design of LMBs.

The DAL approach described above allows for the rapid exploration of a defined parameter space; however, being able to quantify the intercomponent correlations would further empower the establishment of general design principles and intelligent exploration of a potentially much larger parameter space. Conventional efforts on decoding correlations are at best qualitative, with limited testing data by variable-controlled experiments and repeated validations. Further, when analyzing the topological network of two-dimensional pairwise correlations (i.e., between two components), there can be substantial disagreement between the positive and negative messages on one specific correlation, making it hard to extract valuable information (discussed in Supplementary Fig. 7). It is thus challenging to holistically evaluate two-dimensional correlations due to the lack of experimental verification of the whole parameter space.

**Fig. 4:**
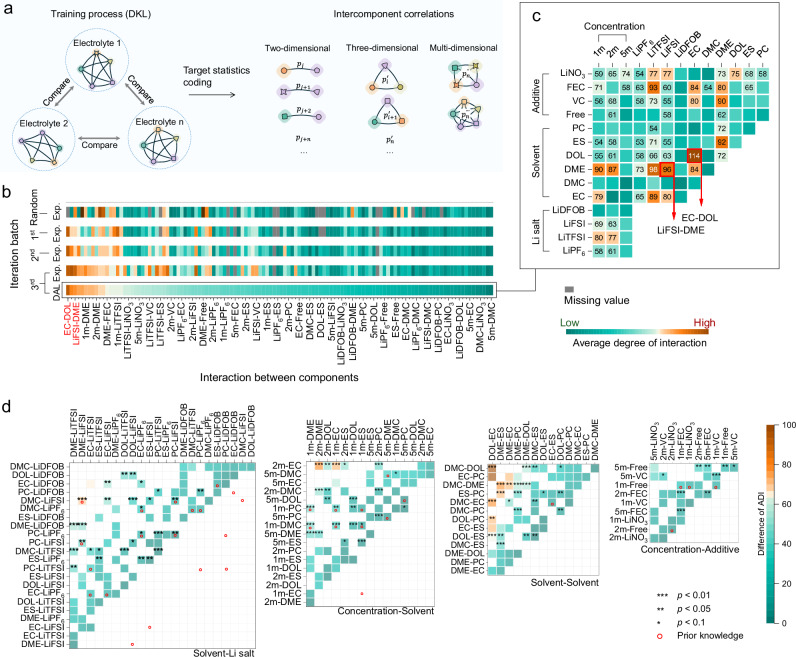
The quantification decoding of intercomponent correlations with DAL-TSC and discovery of new knowledge for electrolyte design. **a** Illustrative scheme showing the decoding of correlations among components in varied dimensions via DAL, where *p* represents the quantitative correlation. **b** The evolution of 121 couples’ correlation between components (average degree of interaction, color coded, quantified by TSC) during iterative optimization. The quantification based on the experimental validation data (denoted as Exp. in the diagram), is compared to that based on DAL’s predictions after each optimization. The values were calculated by averaging all predicted results involving two components, thereby illustrating the impact of the pairwise interactions captured by DAL on the battery cycle life, detailed in “Methods”. **c** The quantified pairwise correlations based on DAL, represented by specific values calculated by averaging all predicted results involving two components. **d** The detailed mapping of pairwise interactions between two components; the pairwise interactions are grouped into 7 groups based on types of the two components, as labelled at the bottom of the maps (four maps are shown here as representative and the other three in Supplementary Fig. 10). The _*_ shown at each pixel means the significance level of each pair of comparison in hypothesis testing based on the experimental dataset: _***_: *p* < 0.01; _**_: *p* < 0.05; _*_: *p* < 0.1. The red circles in the mapping represent prior knowledge reported in the literature.

In contrast, the methodology proposed herein allows for a deeper understanding of the general design principles. We now develop a TSC technique (“Methods” section) to quantify correlation information among components from the electrolyte formulas recommended by DAL (Fig. 4a). TSC is an efficient technique that converts categorical features into numerical features with minimal information loss^56^. Specifically, TSC addresses categorical features (e.g., the co-presence of DME and DOL) by substituting the category of the *k*-*th* electrolyte sample with a numeric feature equal to a target value. It estimates the expected target value (e.g., cell lifespan in this work) conditioned by the category (“Methods” section). With the DAL iteration’s ability to globally represent the entire parameter space and to offer holistic outcome prediction, TSC allows us to statistically quantify the impact of a specific component correlation on cell lifespan (herein defined as average degree of interaction, (ADI)) (“Methods” section). Utilizing TSC, the correlation between two components (two-dimensional) or among multiple components (multi-dimensional) can be quantified. The mapping plot in Fig. 4b depicts the evolution of component interactions captured by our DAL approach with different iterations, specifically highlighted by the color order from the initial to third iteration (details in Supplementary Data 2 and Supplementary Fig. 8). The transformation of the ADI values from a random distribution to an ordered pattern (Fig. 4b) shows that the DAL approach equipped with TSC quantification (DAL-TSC) enables systematic improvement in capturing component interactions as iterations progress. Further correlation analysis between experiment- and DAL-derived ADI values is discussed in Supplementary Fig. 9. Notably, the ADI value provides a statistical perspective for electrolyte chemistry, such as the literature-reported correlation of DME-LiFSI (ADI: 96)^55,57^ and the newly identified correlation of EC-DOL (ADI: 114), offering further insights into electrolyte design (Fig. 4c).

In fact, due to DAL’s rich representation of the entire electrolyte space, as we now show (Fig. 4c), the correlation quantification identified a broad range of knowledge. Using the two-dimensional interaction as an example, with the TSC-quantified data we assessed the impact of a specific pair of components on the cell lifespan. Figure 4d depicts the quantitative comparison of pairwise ADIs for various pairs of components, wherein each pixel represents a comparison between two pairs sharing a common element. A colored pixel indicates that the ADI value of the pair on the horizontal axis exceeds that of the vertical, with the color intensity reflecting the magnitude of this difference. We further analyzed the statistical significance of each pairwise comparison via hypothesis testing based on all available experimental data (quantified as *p*, Fig. 4d). This diagram therefore offers valuable insights for rational design if one aims to pair a specific component with another. Furthermore, ADI not only identifies prior knowledge (i.e., known correlation patterns found from prior reports; marked as red circles in Fig. 4d), but also uncovers new knowledge about previously unknown correlation patterns (detailed in Supplementary Note 6 and Supplementary Data 2). In these maps, the red circles in the colored pixels indicate agreement with established knowledge, while those outside indicate potential discrepancies. Our analysis shows that approximately 85% of previously reported correlations align with our DAL-derived knowledge, validating DAL’s ability to generate meaningful insights for electrolyte design, while remaining discrepancies reflect limited literature statistics and the DAL framework’s focus on interactions that enhance lifespan. The validity of this knowledge is shown by the zero-shot optimization as discussed later. Therefore, DAL-TSC demonstrates a much deeper understanding of the design principles than current literature reports, and we can follow these rules for rational design in a large parameter space. We also globally quantified the correlations among three components (detailed in Supplementary Fig. 11), which is beyond the capacity of conventional manual-based quantification methods. In fact, we speculate that the DAL approach can in principle quantify even higher-dimensional intercomponent correlations (Supplementary Fig. 12). This significantly expands the ability for in-depth exploration and autonomous design of more diverse electrolyte systems.

**Fig. 5:**
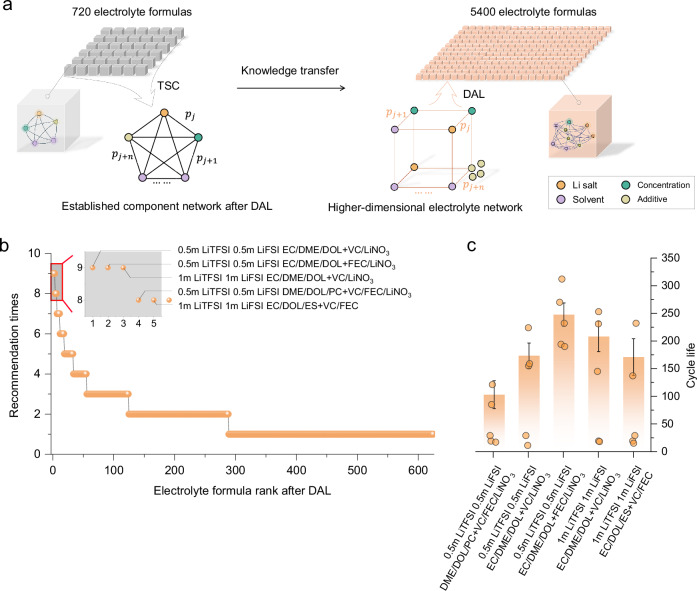
Exploration in a larger parameter space. **a** Illustration of the targeted expansion of the electrolyte parameter space from 720 to 5400 by releasing some parameter constraints, yielding more complex formulas. During the generalization, TSC encodes the electrolyte space network using the correlation knowledge captured from the original parameter space and then transfers the DAL-learned knowledge into a higher-dimensional space and achieves representation in the expanded space. **b** The results of a zero-shot DAL optimization showing the times of recommendation during the DAL optimization for ranking electrolytes, in which five electrolyte formulas were recommended more than eight times. **c** The statistics on the battery cycle life for the validation of these five electrolytes; the length of each bar represents the mean cycle life from repeated measurements with standard deviation; note that the PC-contained electrolyte shows a particularly short cycle life due to the large uncertainty associated with the PC component during optimization (detailed in Supplementary Fig. 14). *n* = 5 independent cells per electrolyte formulation.

Commercial electrolytes typically contain multiple more than two solvents and additives at varying concentration ratios, creating a parameter space that is prohibitively complex and time-consuming to explore through exhaustive experimental testing. Using the DAL approach, which naturally captures the knowledge of electrolyte design, including the reported prior knowledge, we can design electrolytes of higher orders of complexities in a notably more efficient manner by transferring the knowledge. Here, using the same chemical components as originally used and releasing some parameter constraints, we expanded the targeted parameter space from 720 to 5400 (Fig. 5a).

We now use the developed TSC method to integrate experimental data from the original parameter space into this expanded parameter space. TSC captures and encodes key knowledge of component correlations that can be shared between the original and the current electrolyte exploration tasks (Fig. 5a and “Methods”). The knowledge gained on component correlations discussed above was then integrated into the DAL model, enabling it to learn the similarities and differences between these two tasks. The model can thus efficiently transfer the knowledge and generalize its inferences to this new electrolyte design problem (discussed in Supplementary Fig. 13). To demonstrate the success of electrolyte exploration within an expanded parameter space, we utilized the DAL approach in a zero-shot manner (i.e., a non-iterative training without performing any experimental testing). We repeatedly ran ten DAL optimizations, each recommending 32 high-performance electrolyte formulas through TS The times of recommendation for each recommended electrolyte formula were recorded (shown in Fig. 5b), from which we can identify electrolytes that are more likely to engender better Li^0^ reversibility.

Experimental validation was performed on the top five electrolytes recommended by DAL. The experimental results show that among these, four electrolytes achieved stable cell cycling for over 1800 h (Supplementary Fig. 15). The cells using the four electrolytes reaching an average cycle life of 200.6 cycles (Fig. 5c), which is 1.6 times longer than the average lifespan of the original parameter space after iteration (Fig. 3a). The ability of DAL to rapidly optimize a large parameter space of 5400 in a short time and with fewer resources confirms its high efficiency in exploring complex electrolyte systems through capturing the component correlations, which will facilitate autonomous exploration of complex electrolyte systems and accelerated discovery of materials.

**Fig. 6:**
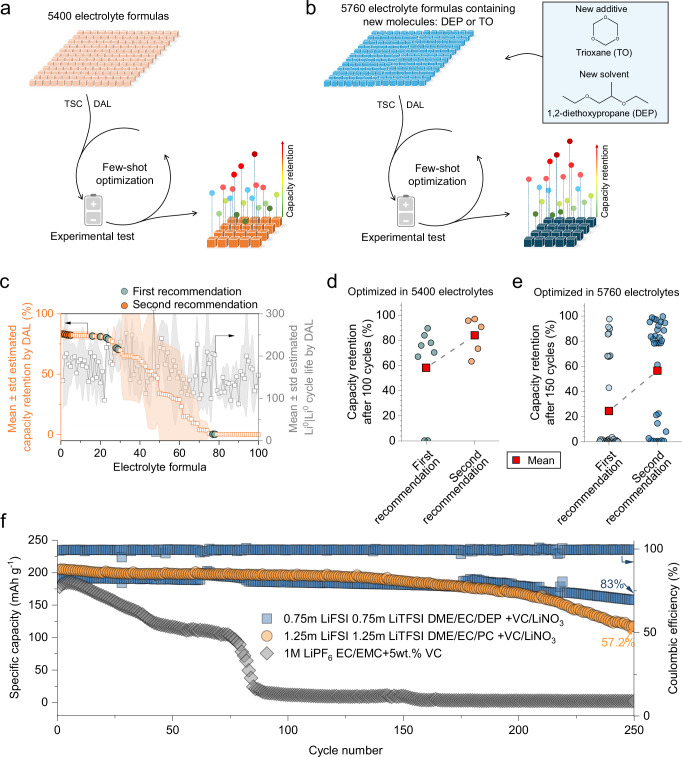
Electrolyte exploration for Li^0^||LMO full cell. **a**, **b** The few-shot DAL optimization with knowledge transfer to exploration of electrolytes for Li^0^||LMO full cells in (**a**) the existing parameter space and (**b**) the parameter space with new molecules. For the TSC coding, the values obtained from the both sets of experimental data (Li^0^||Li^0^ and Li^0^||LMO full cells) are multiplied to generate the input codes for representing the parameter space of the Li^0^||LMO full cell optimization. **c** Mean and standard deviation (mean ± std) of estimated Li^0^||NCM811 capacity retention (yellow squares) in relation to the Li^0^||Li^0^ cell cycle life (gray squares) for the down-selected 100 electrolytes, which are ranked by capacity retention. The top recommended electrolytes in the two rounds are marked as grey and yellow circles. **d** The statistical representation of 100-cycle Li^0^||NCM811 cell capacity retention during the first and second recommendations for optimization in the existing space of 5400. **e** Statistical representation of 150-cycle Li^0^||NCM811 cell capacity retention for optimization in the new space of 5760, including new molecules. **f** The cycling performance for validation of the recommended electrolyte formulas in comparison to the benchmark electrolyte.

Practically, an advanced electrolyte must withstand both Li^0^ negative electrode and high voltage positive electrode simultaneously. The positive electrodes, particularly the aggressive high nickel-content oxides, often impose different, if not conflicting, requirements on the electrolytes and interphases, which means the design of a full cell envelops more interphasial reactions and thus may appear more challenging. Furthermore, the dynamically advancing discovery of new molecules in the field may continue to benefit electrolyte formula for the industry; integrating these new molecules into existing parameter space is important but may cause combinatorial explosion of the space. Here, the framework integrating DAL and TSC is expected to handle such challenges by actively learning the hidden component interactions and recommending high-performance electrolytes with only a small number of additional experimental tests, as illustrated in Fig. 6a, b.

Here, given that electrolytes’ stability with Li^0^ negative electrodes (evidenced by Li^0^||Li^0^ cell performance) is a prerequisite for achieving good cycling performance in full cells (detailed in Supplementary Fig. 16), we leveraged the knowledge captured by DAL for optimizing electrolytes for Li^0^||LMO full cells through few-shot optimization. The down-selected group of top 100 electrolytes (ranked during the Li^0^||Li^0^ cell optimization above) serves as the parameter space for Li^0^||LMO full cell optimization (Fig. 6a, detailed in “Methods”). The top 8 recommended electrolytes were prioritized for experimental testing in full cells employing a 50 μm-thick Li^0^ negative electrode paired with a LiNi_0.8_Co_0.1_Mn_0.1_O_2_ (NCM811) positive electrode (areal capacity: 2.03 mAh cm^−^^2^) using 1/3C charge/discharge protocol. The experimental data (100-cycle capacity retention, raw data shown in Supplementary Fig. 17) served as training inputs for subsequent TSC encoding (i.e., the expected target value is capacity retention) and DAL few-shot optimization, with the results shown in Fig. 6c. As a validation, the second-round recommended top 5 electrolytes delivered an average 100-cycle capacity retention of 84.0% (Fig. 6d, voltage profiles in Supplementary Figs. 17 and 18), substantially outperforming the 58.2% retention of the initial 8 electrolytes—a significant enhancement achieved with only one DAL iteration. This demonstrates the effectiveness of the DAL-TSC learning framework in knowledge transfer to optimization of the full-cell configuration.

Furthermore, to address the practical challenge of incorporating newly discovered molecules into existing parameter space, which causes combinatorial explosion, we developed a few-shot optimization workflow under the DAL-TSC framework. As a proof of the concept, we included two recently reported molecules—1,2-diethoxypropane (DEP) as a solvent molecule and trioxane (TO) as an additive (*19, 20*)—and constructed a parameter space encompassing 5760 new electrolyte formulas. For TSC coding the initial ADIs of DEP-or TO-related interactions were assigned as the prior $p$, which corresponds to the mean values of existing interactions. One batch (32 electrolytes) of experimental cell testing was performed to serve as the input for the few-shot DAL optimization, and the top 32 recommended electrolytes were tested for validation (Fig. 6b, see “Methods” for details). Specifically, with a single-round experimental test of 32 DEP- and TO-containing electrolytes, the mean value of the cell capacity retention increases by 131% from 24.4% (first recommendation) to 56.5% (second recommendation) (Fig. 6e and Supplementary Tables 5 and 6). Note that here, 150-cycle capacity retention was used in light of the generally improved performance with the new molecules. Specifically, the electrolyte formulated as 0.75 m LiTFSI 0.75 m LiFSI DME/EC/DEP + VC/LiNO_3_ delivers 83% capacity over 250 cycles, which is greatly improved over the previous optimized electrolytes (i.e., 57.2% for 1.25 m LiTFSI + 1.25 m LiFSI DME/EC/PC + FEC/LiNO_3_; Fig. 6f). This demonstrates that our DAL framework can effectively transfer previously acquired knowledge to guide electrolyte design involving newly introduced molecular components. By initializing unknown interactions with statistically derived priors and incorporating only a small number of experimental measurements, the framework dynamically expands the searchable space, enabling efficient optimization as new molecules are discovered without restarting the process.

## Discussion

Designing stable electrolytes for LMBs is time- and resource-intensive, due to complex chemical interactions that are difficult to predict. We addressed this challenge by developing a two-stage DAL framework that leverages ML to rapidly explore vast and highly discrete parameter spaces. The deep kernel learning method can quickly and effectively capture complicated correlations among electrolyte components. By integrating it with DAL, we rapidly discovered electrolytes that greatly improve the reversibility of Li^0^||Li^0^ cells after only three iterations, consuming just 4.4% of the resources required for exhaustive testing. With the captured knowledge expressed as correlations among components, we can then efficiently explore a higher-dimensional and much larger parameter space via target statistics coding, and further identified additional electrolytes that enable cells to cycle over 1800 h. The two-stage DAL framework is further verified by identifying electrolytes with improved performances in Li metal full batteries and with newly added molecules through few-shot optimizations. This work demonstrates how integrating ML with battery science can accelerate discovery in highly complex and discontinuous chemical systems, providing a framework accessible to both materials scientists and ML researchers.

In this work, we show that a DAL framework, built around a DKL surrogate and experimentally derived interaction descriptors, can accelerate the discovery of long-lifespan electrolyte formulations by steering experiments toward promising regions of a combinatorial formulation space. This implementation, however, is currently restricted to a fixed, experimentally accessible component library and to the specific cell chemistry and cycling protocol studied here; it does not perform de novo, zero-shot molecular design, but instead enables data-efficient optimization and few-shot expansion, for which newly introduced components still require targeted experiments. Chemical information is encoded in a formulation-centric manner through interaction statistics (such as ADIs), and TSC/ADI act as post hoc abstractions rather than learned molecular embeddings, which is pragmatic for small, noisy datasets but limits the resolution of structure-property relationships and generalization across chemically distant components. In addition, the current framework models predictive uncertainty under a homoscedastic noise assumption, such that uncertainty estimates primarily reflect epistemic uncertainty arising from sparse sampling and limited coverage of the formulation space. As a result, input-dependent aleatoric uncertainty is not explicitly quantified, which constrains the assessment of experimental variability and reproducibility across heterogeneous conditions. Looking ahead, as larger, task-aligned electrolyte datasets emerge, molecular foundation models and pretrained mixture-level encoders could be integrated with these interaction-based features to improve generalization, while heteroscedastic uncertainty modeling could further enhance the characterization of experimental variability and reproducibility, all while preserving data efficiency and enabling a gradual transition from formulation-level optimization toward more genuine molecular design.

## Methods

### Experimental

The electrolytes in this work were prepared with rigorous control of the content of water. All lithium salts (LiPF_6_ (99.99%, Aladdin), LiTFSI (99.99%, Aladdin), LiFSI, LiDFOB, LiNO_3_, 99.99%, Aladdin) were dried in a vacuum glass oven for 72 h. The solvents (EC (99.99%, battery-grade, Aladdin), DMC (99.99%, battery-grade, Aladdin), DME (99.99%, battery-grade, Aladdin), DOL (99.99%, battery-grade, Aladdin), PC (99.99%, battery-grade, Aladdin)) and additives (ethylene sulfite (ES, 99.99%, battery-grade, Aladdin), VC (99.99%, battery-grade, Aladdin) and FEC (99.99%, battery-grade, Aladdin)) were treated with molecular sieves for over 96 h to remove trace water. The electrolytes were then prepared based on the targeted formulas in an Ar glove box (< 0.01 ppm H_2_O, <0.01 ppm O_2_). To prevent overheating, the salts and additives were added slowly at ambient temperature. After stirring and then standing respectively for 24 h, the electrolytes are ready for assembling of cells. The algorithm-recommended electrolytes were used for cell measurements regardless of the color, states of dissolving, and any possible between-component reactions, as we deem the study as a non-expert process. To assemble the Li metal symmetric cells, two lithium metal disks of 18 mm in diameter and of 600 µm in thickness were used as the electrodes with a polyolefin separator (Celgard 2500 with Al_2_O_3_ coating, thickness: 25 µm, porosity: 55%, Gurley value: 200 s) in 2032-coin cell cases. For all cells, 50 µL of the electrolyte was used. The Li striping/plating cycling test was conducted with a current of 0.5 mA cm^−2^ for 4 h discharge and 4 h of charge at 25 ± 0.05 °C in environmental chamber, with a voltage cut-off of 0.5 V.

For the fabrication of the full cells, positive electrodes coated with LiNi_0.8_Co_0.1_Mn_0.1_O_2_ (NCM811), purchased from Guangdong Canrd New Energy Technology Co., Ltd, China. (Model: SY31XX), were cut into 10 mm diameter pieces in an Ar glove box, and dried in a vacuum oven at 100 °C for 24 h. The current collector was aluminium foil with a thickness of 12 μm and an areal density of 3.5 mg cm^−2^; the mass fraction of active material was 94.5%. The areal loading for the NCM811 positive electrodes is 10.8 mg cm^−2^. For the lithium metal negative electrode, a fresh lithium foil without surface treatment (10 mm in diameter and 50 µm thick, Tianjin Tianneng Co., Ltd, China) was pressed onto a hydrogen-reduced copper foil (16 mm in diameter) to ensure good contact between the lithium and copper foils. To assemble the Li^0^||LMO full cells, the prepared positive electrodes, a polyolefin separator (Celgard 2500 with Al_2_O_3_ coating), and the Li/Cu negative electrode were assembled into 2032-coin cell cases, each containing 50 µL of electrolyte. The assembled full cells were tested at 25 ± 0.05 °C using 1/3 C charge/discharge protocol with a voltage window of 3.0–4.3 V.

### Computational

#### Deep active learning

For electrolyte optimization, a deep kernel learning (DKL)-based AL approach is proposed. AL is a Bayesian optimization-based ML approach for the global optimization of black-box objective functions that are noisy and expensive to evaluate^49^; this is the characteristics of the objective function to be optimized in the electrolyte design problem for LMBs in this study. Generally, AL comprises of a probabilistic surrogate model and an acquisition function, in which the surrogate model, such as Gaussian process is used for approximating the expensive and noisy objective function, and the acquisition function is to sample-efficiently probe the parameter space by balancing exploration and exploitation^50^.

For probabilistic surrogate modelling, Gaussian process is commonly used and can be described by the mean and covariance function^58^. For the objective function *f*(***x***), its GP model can be described as

2 $$f\left({{{\boldsymbol{x}}}}\right) \sim {GP}\left(m\left({{{\boldsymbol{x}}}}\right),\kappa \left({{{\boldsymbol{x}}}},{{{{\boldsymbol{x}}}}}^{{{{\prime} }}}\right)\right)$$

where $m({{{\boldsymbol{x}}}})$ denotes the mean of the Gaussian process, and $\kappa ({{{\boldsymbol{x}}}},{{{{\boldsymbol{x}}}}}^{{{{\prime} }}})$ denotes the covariance function. In this work, the variable ${{{\boldsymbol{x}}}}$ is an electrolyte formula with certain solvents, lithium salts, salt concentration and additives, and $f({{{\boldsymbol{x}}}})$ models the mapping from an electrolyte formula ${{{\boldsymbol{x}}}}$ to the corresponding cell lifespan.

When the observation of the objective function is coupled with noise, that is

3 $$y=f\left({{{\boldsymbol{x}}}}\right)+\epsilon$$

where $f\left({{{\boldsymbol{x}}}}\right)$ denotes the unknown true cycle life of the cell for a given electrolyte formula ${{{\boldsymbol{x}}}}$, while the observed cycle life $y$ is experimentally measured and contains uncertainty due to experiment noise $\epsilon$, which we assume follows a normal distribution ${{{\mathcal{N}}}}(0,\,{\sigma }^{2})$. The posterior distribution predicted based on the GP, given a dataset ${{{\mathcal{D}}}}={\left\{\left({{{{\boldsymbol{x}}}}}^{\left(i\right)},{y}^{(i)}\right)\right\}}_{i=1}^{N}$, can be obtained as

4 $$p\left(y|{{{\mathcal{D}}}}\right)={{{\mathcal{N}}}}\left(\mu \left({{{\boldsymbol{x}}}};{{{\mathcal{D}}}}\right),{\sigma }^{2}\left({{{\boldsymbol{x}}}};{{{\mathcal{D}}}}\right)\right)$$

where

5 $$\mu \left({{{\boldsymbol{x}}}}; {{{\mathcal{D}}}}\right)={{{{\boldsymbol{K}}}}}^{T}\left({{{\boldsymbol{x}}}}\right){\left({{{\mathbf{\Sigma }}}}+{{{\mathbf{\Sigma }}}}^{2}{{{\boldsymbol{I}}}}\right)}^{-1}y$$

6 $${\sigma }^{2}\left({{{\boldsymbol{x}}}}; {{{\mathcal{D}}}}\right)={{{\boldsymbol{K}}}}\left({{{\boldsymbol{x}}}},{{{\boldsymbol{x}}}}\right)-{{{{\boldsymbol{K}}}}}^{T}\left({{{\boldsymbol{x}}}}\right){\left({{{\mathbf{\Sigma }}}}+{\sigma }^{2}{{{\boldsymbol{I}}}}\right)}^{-1}{{{\boldsymbol{K}}}}$$

with $${{{\boldsymbol{K}}}}\left({{{\boldsymbol{x}}}}\right){=\left[\kappa \left({{{\boldsymbol{x}}}},{{{{\boldsymbol{x}}}}}^{\left(1\right)}\right),\ldots,\kappa \left({{{\boldsymbol{x}}}},{{{{\boldsymbol{x}}}}}^{\left(N\right)}\right)\right]}^{T}$$, and $${\left[\Sigma \right]}_{i,j}=\kappa \left({{{{\boldsymbol{x}}}}}^{\left(i\right)},{{{{\boldsymbol{x}}}}}^{\left(j\right)}\right).$$

The selection of covariance function $\kappa ({{\cdot }},{{\cdot }})$ is important for the GP model, which is to capture the similarity of any two data samples, and thereby specifies the smoothness or oscillation degree of the black-box objective function^59^. Selecting a suitable covariance function can largely enhance the modeling performance of GP. However, standard GP kernel functions, such as RBF kernel, are found difficult to capture the non-stationary structure in a complex system such as the electrochemical operation of LMBs, and thereby deteriorate the performance of AL for the design of LMB electrolytes. To address this issue, GP based on DKL was utilized in our work, which combines the representational power of deep neural network with the reliable uncertainty estimates of GP. The deep kernel^7,49^ is defined as Eq. (1) in the main text, and a variational inference algorithm was used to maximize the marginal likelihood function of DKL-GP to obtain its parameters.

For the acquisition function, a TS strategy^60^ was employed to determine the next electrolyte formula ${{{{\boldsymbol{x}}}}}^{(t)}$. In each iteration, TS draws a sample from the posterior predictive distribution $p({y|}{{{{\mathcal{D}}}}}_{t})$ and selects the formula that maximizes the outcome:

7 $${{{{\boldsymbol{x}}}}}^{\left(t\right)}={{\arg }}{\max }_{{{{\boldsymbol{x}}}}}h\left({{{\boldsymbol{x}}}}\right),\,h\left({{{\boldsymbol{x}}}}\right) \sim p({y|}{{{{\mathcal{D}}}}}_{t})$$

where ${{{{\mathcal{D}}}}}_{t}={\left\{\left({{{{\boldsymbol{x}}}}}^{\left(i\right)},{y}^{\left(i\right)}\right)\right\}}_{i=1}^{t-1}$ represents the history of evaluations. The selected electrolyte formula ${{{{\boldsymbol{x}}}}}^{(t)}$ is then experimentally evaluated, yielding an observed cycle life ${y}^{(t)}$, and the dataset is updated as ${{{{\mathcal{D}}}}}_{t+1}={{{{\mathcal{D}}}}}_{t}\cup \{\left({{{{\boldsymbol{x}}}}}^{(t)},\,{y}^{(t)}\right)\}$. By iteratively sampling from the posterior predictive distribution, TS efficiently explores the electrolyte design space while balancing exploration and exploitation to accelerate the identification of optimal electrolyte compositions for extended cycle life.

#### Target statistics coding

Quantifying correlation information among electrolyte components requires transforming discrete categorical features (e.g., the co-presence of DME and DOL) into numerical representations. This transformation is essential for knowledge transfer between original and target electrolyte exploration tasks while facilitating efficient optimization. Target statistics coding (TSC) is an efficient method that converts categorical features into numerical features with minimal information loss. Specifically, TSC addresses categorical features by substituting the category of the *k*-th electrolyte sample with a numeric feature equal to a target statistic. Specifically, we encode the relational feature ${{{{\boldsymbol{X}}}}}_{i}$ of the electrolyte in the $k$-th sample as its expected component correlation value, which can be estimated as^56^:

8 $$\phi \left({{{{\boldsymbol{X}}}}}_{i}^{\left(k\right)}\right){\mathbb{=}}{\mathbb{E}}\left(y|{{{{\boldsymbol{X}}}}}_{i}={{{{\boldsymbol{X}}}}}_{i}^{\left(k\right)}\right)=\frac{{\sum }_{j=1}^{N}{{\mathbb{1}}}_{\left\{{{{{\boldsymbol{X}}}}}_{i}^{\left(j\right)}={{{{\boldsymbol{X}}}}}_{i}^{\left(k\right)}\right\}}\cdot {y}_{j}}{{\sum }_{j=1}^{N}{{\mathbb{1}}}_{\left\{{{{{\boldsymbol{X}}}}}_{i}^{\left(j\right)}={{{{\boldsymbol{X}}}}}_{i}^{\left(k\right)}\right\}}}$$

where ${{{{\boldsymbol{X}}}}}_{i}^{\left(j\right)}$ represents the relational feature $i$ in the $j$-th sample, ${y}_{j}$ is the target value (i.e., cell lifespan or capacity retention in this work), and ${\mathbb{1}}_{\{ \}}$ is the indicator function.

Equation (8) shows that the expected correlation value for relational feature ${{{{\boldsymbol{X}}}}}_{i}$ is approximated as the ADI across all electrolyte samples sharing the same relational feature as ${{{{\boldsymbol{X}}}}}_{i}^{\left(k\right)}$. Since this estimation may be unreliable for low-frequency features, a prior $p$ is introduced for smoothing, formulated as:

9 $$\phi ({{{{\boldsymbol{X}}}}}_{i}^{\left(k\right)})={\lambda }_{i}\,{{\cdot }}\,{\mathbb{E}}\left(y|{{{{\boldsymbol{X}}}}}_{i}={{{{\boldsymbol{X}}}}}_{i}^{\left(k\right)}\right)+\left(1-{\lambda }_{i}\right)\cdot p$$

where $p$ represents the average target value in the electrolyte dataset, and ${\lambda }_{i}$ is a weighting factor defined as

10 $${\lambda }_{i}=\frac{1}{1+\exp \left(-\frac{{\sum }_{j=1}^{N}{{\mathbb{1}}}_{\left\{{{{{\boldsymbol{X}}}}}_{i}^{\left(j\right)}={{{{\boldsymbol{X}}}}}_{i}\right\}}-C}{d}\right)}$$

Here, $C$ and $d$ are hyperparameters that control confidence and smoothing, respectively. Specifically, $C$ represents the threshold number of samples required for a reliable estimation of the conditional mean, while $d$ determines the degree of smoothing. This formula effectively balances empirical observations with prior knowledge, providing reliable estimates even for electrolyte relational features with limited experimental data points.

ADI and knowledge transfer: To effectively transfer knowledge across electrolyte optimization tasks, we utilize TSC to quantify key component correlations from the high-performing electrolyte formulas recommended by DAL in the first stage. These quantified relational features encompass pairwise, triplet, and higher-order correlation values, capturing the underlying interactions among the components. Specifically, for the $k$-th sample’s electrolyte formula composed of $L$ distinct components, we represent its feature vector as:

11 $${{{{\boldsymbol{X}}}}}^{\left(k\right)}=\left[\,\left\langle {x}_{l}^{\left(k\right)},{x}_{s}^{\left(k\right)}\right\rangle,\,\ldots,\left\langle {x}_{1}^{\left(k\right)},{x}_{2}^{\left(k\right)},\ldots,\,{x}_{L}^{\left(k\right)}\right\rangle \right],\,1\le l,s\le L$$

where ${x}_{l}^{\left(k\right)}$ represents the individual electrolyte formula feature $l$ (e.g., lithium salt, solvent, additive, concentration) of the $k$-th sample; $\langle {x}_{1}^{(k)},{x}_{2}^{(k)},\ldots,\,{x}_{L}^{\left(k\right)}\rangle$ represents the ($L$-1)-th order relational features (i.e., the interactions among $L$ components). These features are estimated by the expected target values (e.g., cell lifespan) within their respective categories, enabling us to quantify specific component interactions, defined as:

12 $$\phi \left({{{{\boldsymbol{X}}}}}^{\left(k\right)}\right)=\left[\phi \left(\left\langle {x}_{l}^{\left(k\right)},{x}_{s}^{\left(k\right)}\right\rangle \right),\,\ldots,\phi \left(\left\langle {x}_{1}^{\left(k\right)},{x}_{2}^{\left(k\right)},\ldots,\,{x}_{L}^{\left(k\right)}\right\rangle \right)\right],\,1\le l,s\le L$$

For example, the encoding for first-order relationships (i.e., $\phi (\left\langle {x}_{l}^{\left(k\right)},{x}_{s}^{\left(k\right)}\right\rangle )$) can be estimated using Eq. (7), which corresponds to the average cycle life across all electrolyte samples containing the corresponding relational feature $\langle {x}_{l}^{\left(k\right)},{x}_{s}^{\left(k\right)}\rangle$, or estimated by incorporating additional prior information as specified in Eq. (9). This encoding quantifies the interaction between specific components, a metric we refer to as ADI. For instance, the ADI between the DOL and EC components yields an encoding value of approximately 113.7 cycles (i.e., ${{{\rm{ADI}}}}\left(\left\langle {{{\rm{DOL}}}},{{{\rm{EC}}}}\right\rangle \right)=\phi \left(\left\langle {{{\rm{DOL}}}},{{{\rm{EC}}}}\right\rangle \right)=113.7$).

Once the feature vector is obtained, it serves as a comprehensive representation of electrolyte formulas, embedding key component correlation knowledge. In the second stage, this encoded numerical representation is used as input to the DAL framework over an expanded design space, enabling efficient knowledge transfer through two approaches: (1) To generalize the DAL framework in symmetric cells across a larger chemical space, the ADI features derived from experimental data within the initial parameter space were applied to the DAL framework in a zero-shot manner, enabling the recommendation of electrolyte formulas. (2) For generalization in full cells across a larger chemical space—and given that symmetric cell performance is a prerequisite for reliable full-cell cycling—we defined the Li^0^||LMO full-cell optimization space as the top 100 electrolyte formulas recommended by the Li^0^||Li^0^ cell optimization. This served as the starting point for the optimization process. Subsequently, TSC was employed to quantify the influence of relational features on capacity retention, and this quantification was used as input for the next iteration of DAL, facilitating few-shot optimization. (3) For the newly discovered molecules, their associated interactions (here, DEP- or TO-based interactions) were assigned a prior value $p$ (i.e., the mean interaction value in the electrolytes), and DAL was applied in a zero-shot manner to recommend 32 electrolytes for experimental validation in full cells. The measured data obtained from these experiments was then used for the second iteration of DAL.

## Supplementary information

Supplementary Information

Peer Review File

Description of Additional Supplementary Files

Supplementary Data 1

Supplementary Data 2

## Source data

Source Data

## Data Availability

The datasets analysed and generated in this study are included in the supplementary information and are available at Source Data and 10.5281/zenodo.18731009. Source data are provided with this paper.

## Code availability

The code used in this study is released in a publicly available Zenodo repository at 10.5281/zenodo.18731009. The repository includes an Open Source Initiative–approved license. The code can be accessed by downloading the archived release from Zenodo; no additional restrictions apply beyond the terms of the license.
