# Supplementary material for: Deep active learning and knowledge transfer for rapid discovery of lithium metal battery electrolytes
Source: Nat Commun. 2026 Mar 27;17:5146. doi: 10.1038/s41467-026-70973-4 (PMC13250146; doi:10.1038/s41467-026-70973-4)
Supplement: Supplementary file 1 — Supplementary Information [file 41467_2026_70973_MOESM1_ESM.pdf]

Supplementary information

Deep active learning and knowledge transfer for rapid discovery of lithium metal battery electrolytes

*Xufeng Hong[*1], Xizhe Wang[*2], Stephen J. Harris[3], Hongbo Zhao[4], Jiashen Meng[1], Qingshan Jia[2], Qianchuan Zhao[2], Kang Xu[5, †], Quanquan Pang[1, †], Benben Jiang[2, †]*

[1] Beijing Key Laboratory of Theory and Technology for Advanced Batteries Materials, School of Materials Science and Engineering, Peking University, Beijing 100871, China
[2] CFINS, Department of Automation, Beijing National Research Center for Information Science and Technology, Tsinghua University, Beijing 100084, China
[3] Lawrence Berkeley National Laboratory, Berkeley, California 94720, USA
[4] Department of Chemical and Biological Engineering, Princeton University, Princeton, NJ 08544, USA
[5] SES AI Corp., Woburn, MA 01801, USA
** These authors contributed equally to this work*
† Corresponding authors

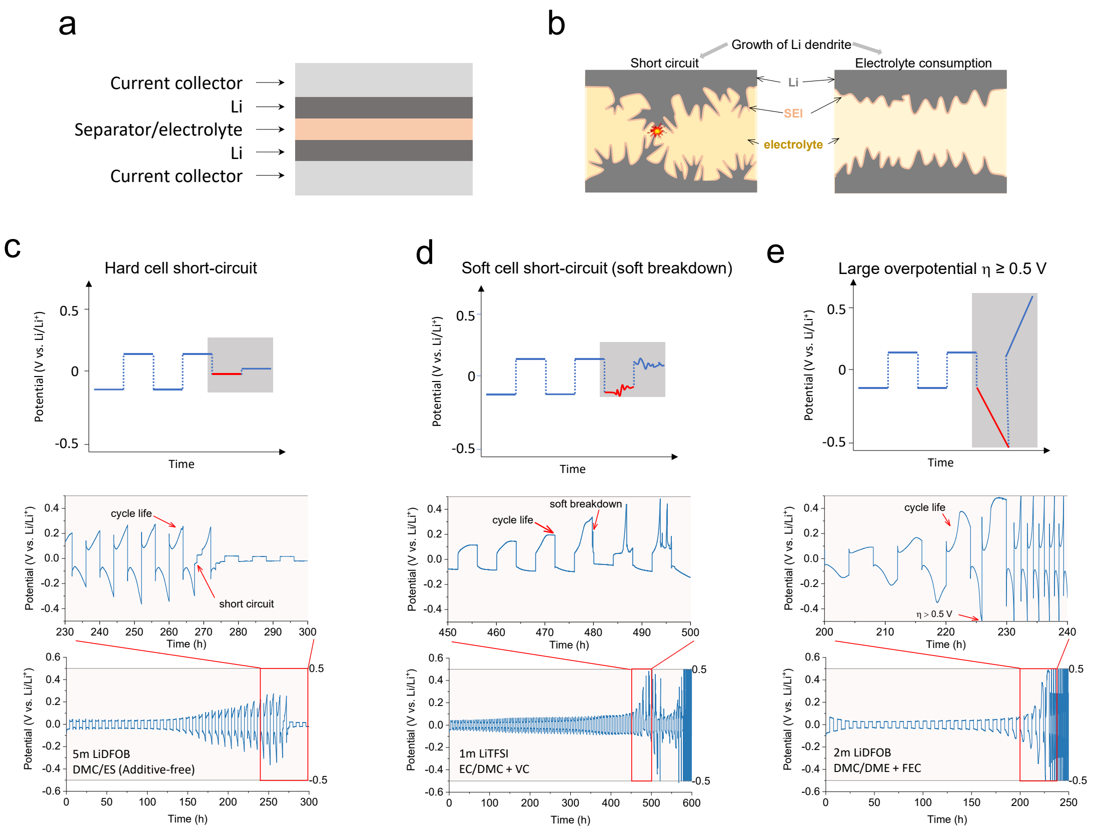

Supplementary Fig. 1 | Electrochemical measurement of Li|Li symmetric cells and the determination of cell cycle life (lifespan). a, The illustrative cell configuration of the Li|Li symmetric cell used in the study. **b**, the schematic diagrams of two major cell failure modes: the hard cell short-circuit, and the continuous electrolyte consumption accompanied by increase of overpotential. **c-e**, Schematic voltage profiles indicating the hard cell short-circuit (**c**), the soft cell short-circuit (soft cell break down) (**d**), and the overly high overpotential during Li plating/stripping (**e**); and one experimental example showing such failure modes. We recognized the importance of accounting for all failure modes of the Li symmetric cells, and thus the cell's lifespan is selected as the measure of cycling stability in our study. Specifically, the cell's lifespan (i.e., the number of Li plating/stripping cycles) is defined as the number of cycles when the cell ends with a hard short circuit, a soft circuit, or an overpotential greater than 0.5 V. This work uses an areal capacity of 2.0 mA h cm^{-2} with a current density of 0.5 mA cm^{-2}, in line with practical requirements for lithium metal anodes (LMAs).

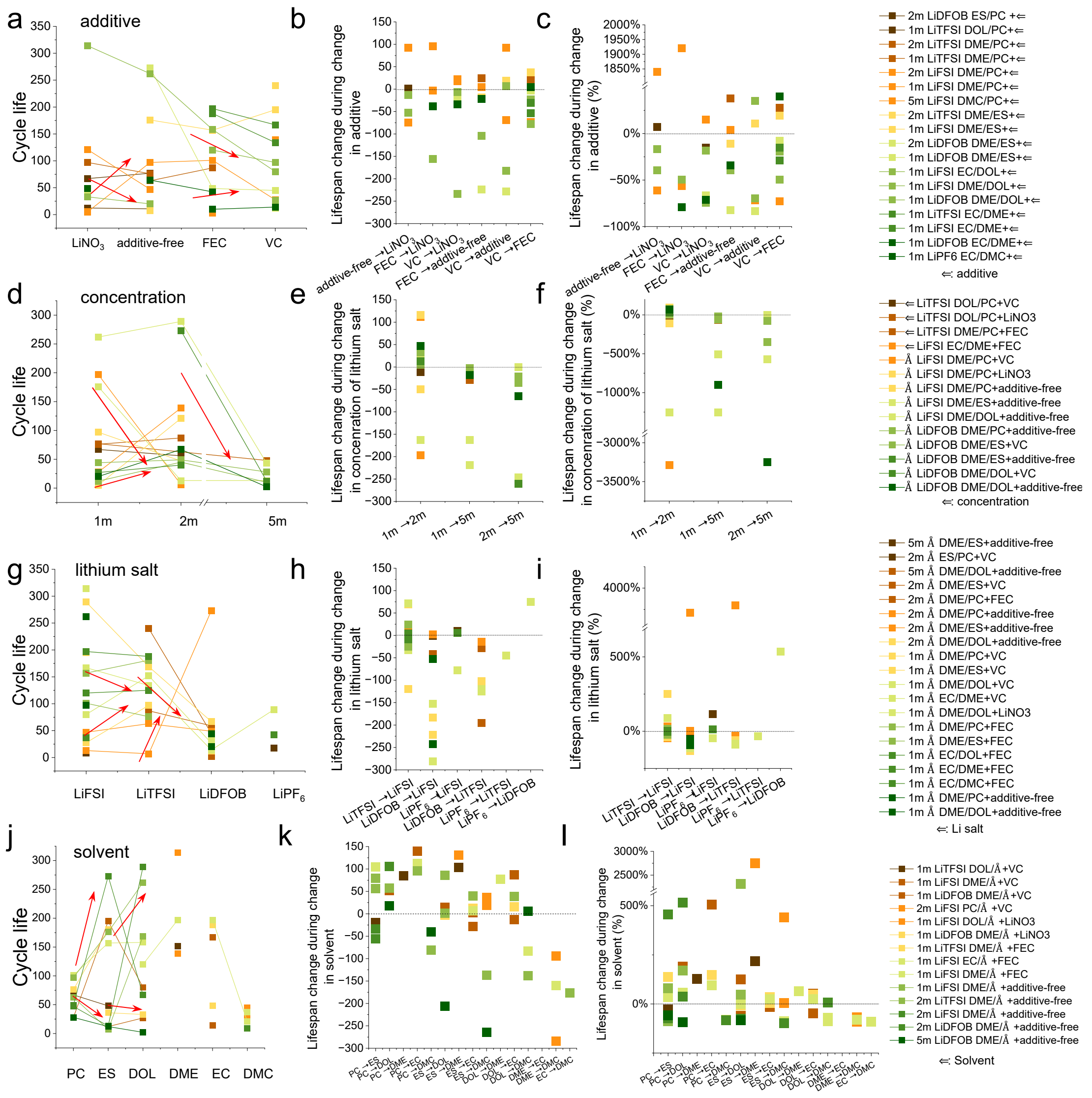

Supplementary Fig. 2 | Experimental verification of the large parameter-performance discontinuity (leading to the discreteness of the parameter space) in relating the electrolyte components with the cell cycle life. A variable-control statistical method was here used to analyze the change of cells' cycle life as one single component is changed in the electrolyte: **a-c**: change of only the additive, **d-f**: change of only the lithium salt concentration, **g-i**: change of only the lithium salt, **j-l**: change of only one solvent. All the data points analyzed here are from the experimentally measured electrolyte formulas during the three DAL iterations. To illustrate the large parameter-performance discontinuity, for each scenario of component change (that is, each row), we plot the raw data of absolute values of cells' cycle life for each electrolyte (left column, **a, d, g, j**), the change of absolute values of cells' cycle life as the component changes from one to another (central column, **b, e, h, k**) and the corresponding relative ratio of change of cells' cycle life (%, right column, **c, f, i, l**). The color-coding legend of each group of data points is shown on the very right, where the square symbols represent the components to be changed. For the figures in the central and right column, the x-axis shows the specific component that is changed, and each change contains multiple data points with each point representing a specific electrolyte (shown as colored squares).

These statistical analyses provide evidence for the high discontinuity between electrolyte components and cell cycle life and thus confirm the nature of high discreteness of the parameter space for the electrolyte optimization problem in this study. Apparently, the Li metal cells' cycle life is highly sensitive to the components of the electrolyte, highlighting the high uncertainty of the impact of modifying a single component on the cells' cycle life (indicated by the red arrows). This is evident by the largely scattered distribution of cells' cycle life changes as shown in **Supplementary Figs. 2b**, **h**, and **k**, and by the wide range of relative changes are shown in **Supplementary Figs. 2c**, **i**, and **l**. This indicates that changing only one of the electrolyte's components may result in a large change of cells' cycle life, but even further, may also lead to largely varied magnitude of cycle life increase or decrease, depending on the type of component to be changed. Notably, a large increase in the lithium salt concentration adversely affects the cycling stability of the cell (**Supplementary Figs. 2e** and **f**).

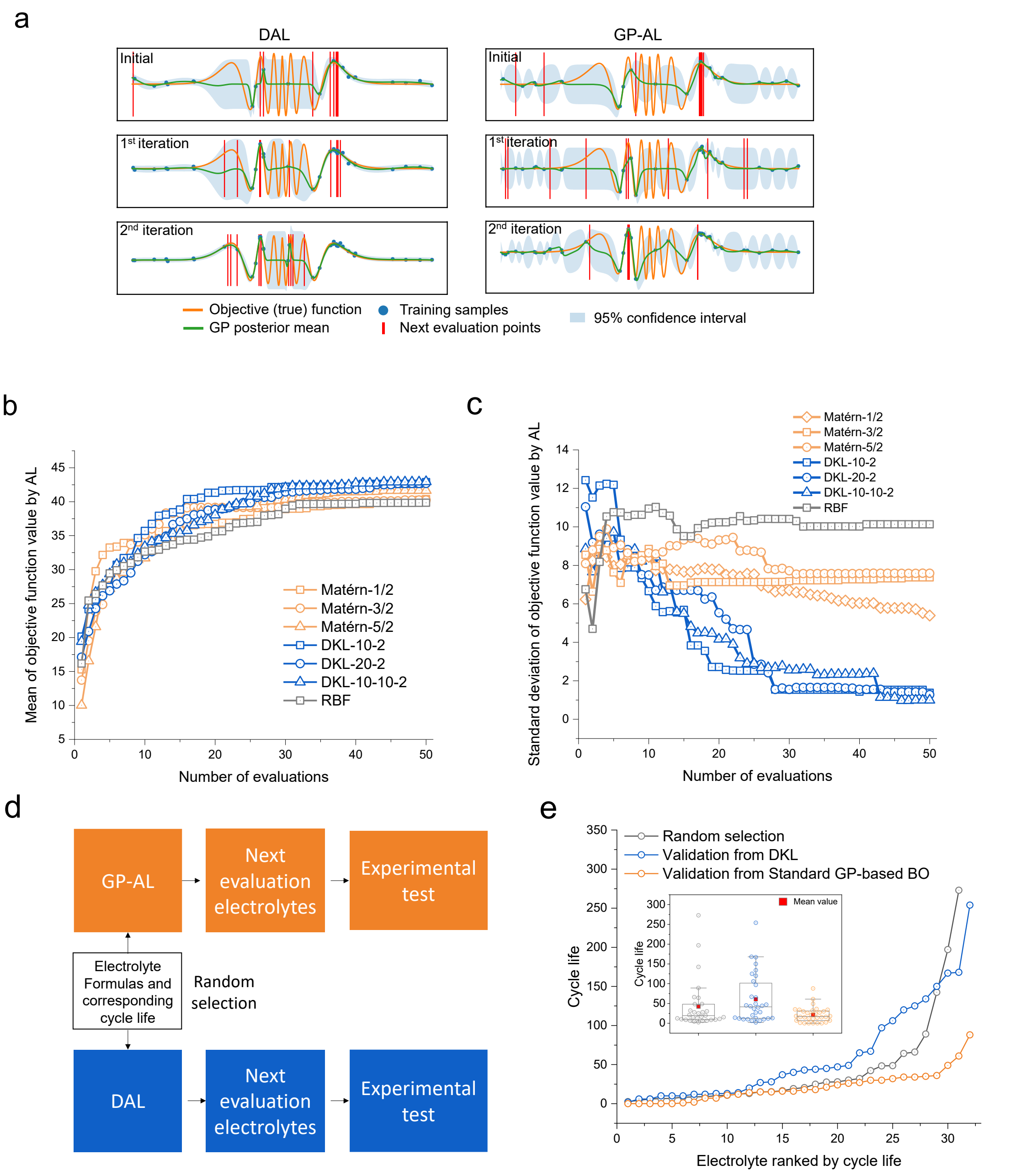

Supplementary Fig. 3 | The advantages of DAL over classical GP-AL for the optimization of an objective function with rapid oscillations. **a**, The comparison of the exploration processes using respectively the proposed DAL and classical GP-AL on a function of $\boldsymbol{f} = (\mathbf{1} - |\boldsymbol{x}|)\,\mathbf{sin}(\mathbf{20}(\mathbf{1} - |\boldsymbol{x}|)^{\mathbf{6}})$ with added noise $\boldsymbol{\epsilon} \sim \boldsymbol{N}(\mathbf{0}, (\mathbf{5\%} \times \mathbf{max}|\boldsymbol{f}|\,)^{\mathbf{2}})$. The standard GP-AL maintained the same optimization settings but was trapped into a local optimal solution during the iteration, resulting in low sample efficiency. In contrast, our DAL approach well harnessed the potent representational capacity of neural networks and autonomously acquired an adaptive kernel function from data for the rapidly oscillating objective function. This adaptive deep kernel, also known as the data-informed kernel, enhances data representation capabilities by combining the structural properties of deep learning algorithms with the flexibility of kernel methods. This results in a more accurate surrogate model and yields superior optimization outcomes in comparison to the GP-AL method which relies on fixed kernel functions (detailed in **Supplementary Note 7**). **b-c**, The mean (**b**) and standard deviation (**c**) of optimization performance provided by DAL and GP-AL methods under different covariance functions in complex numerical scenarios ($\boldsymbol{f} = \mathbf{20} + \sum_{i=1}^{2}[\boldsymbol{x}_i^2 - \mathbf{10}\,\mathbf{cos}(\mathbf{4}\boldsymbol{x}_i^4)]$, $\boldsymbol{i} = \mathbf{1}, \mathbf{2}$, which similarly combines the components with smooth changes and rapidly oscillating behavior, designed to reflect the characteristics of our experimental electrolyte data.) with a 5% noise level. DAL employs fully connected neural networks with three configurations: 10-2, 20-2, and 10-10-2, with 10, 20, and 10 neurons per layer, respectively. The network structure of 10-2 or 20-2 is a fully connected neural network with one hidden layer, and the 10-10-2 network has two hidden layers (each layer has 10 neurons). The results show that the GP-AL optimizations with a Matérn kernel function or a radial basis function (RBF) kernel function exhibit slower convergence, as evidenced by the mean after 50 optimization iterations being significantly lower than that of the DAL approach (**Supplementary Fig. 3b**). Moreover, the GP-AL demonstrates higher standard deviations than the DAL does across multiple experiments, indicating an unstable optimization effect when different initial samples are used (**Supplementary Fig. 3c**). Further details can be found in **Supplementary Note 7**. **d**, An illustrative framework showing how DAL and GP-AL optimizations evolve in experimental validations. **e**, The experimental validation to compare the DAL and GP-AL methods. Randomly selected data were used as training data for both methods, and the recommended electrolyte formulas from each method were tested. The experimental electrolytes recommended by the DAL approach (blue) exhibited a higher lifespan compared to those from the GP-AL approach (orange), demonstrating the superior efficiency of DAL in electrolyte exploration. Inset: Box plots show the median (center line) and the 25th–75th percentiles (box); whiskers extend to the most extreme data points within 1.5× the interquartile range, and points represent individual measurements. n=32 electrolyte formulars for experimental test in each batch.

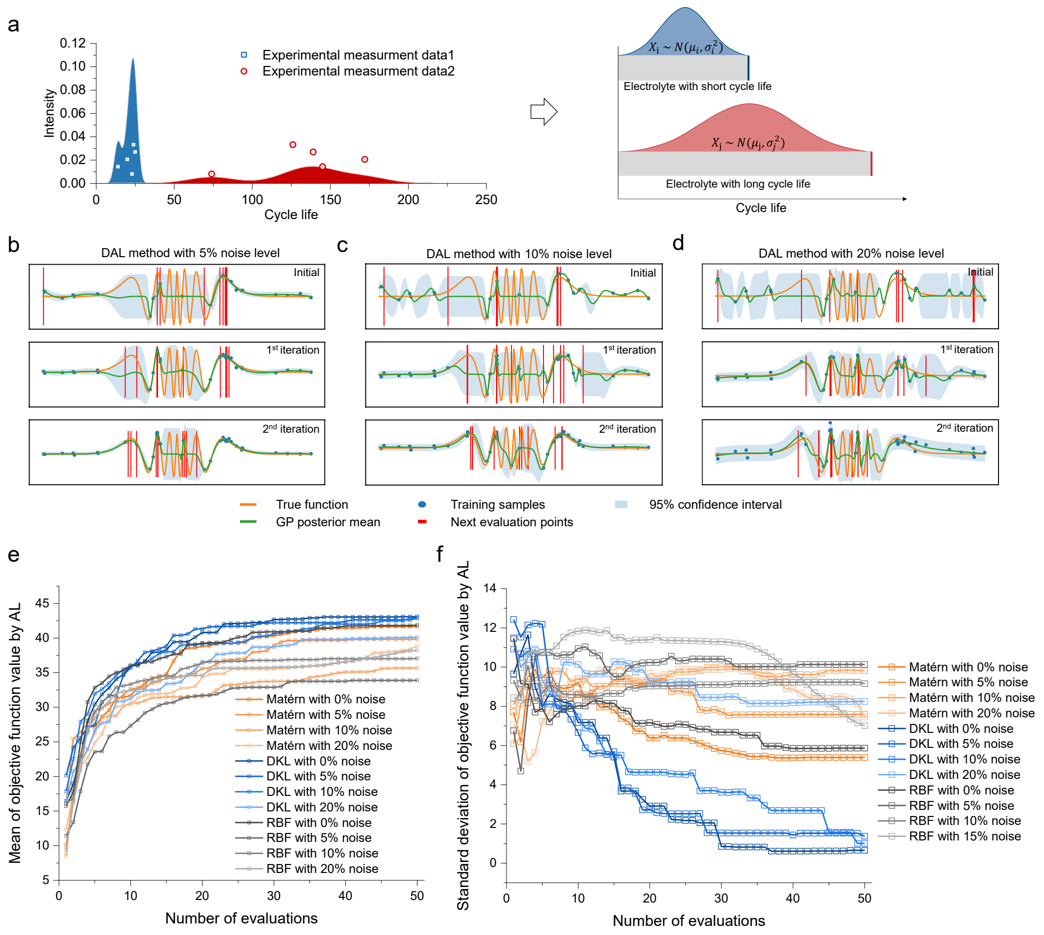

Supplementary Fig. 4 | Comparison of DAL and GP-AL for the optimization of rapidly oscillating objective function applied with various noise levels. a, Experimental measurement data showing that the experimental noise level differs for electrolytes of different ranges of cell cycle life; the left figure shows the distribution of experimentally measured cell cycle life, from which we can see that electrolytes with longer cell cycle life smear across a wide range (red, 50~200 cycles), and in contrast, electrolytes with shorter cell cycle life exhibit a more concentrated distribution (blue, 10~30 cycles). This is mainly owed to experimental variability, which is illustrated by the Gaussian distribution diagrams shown on the right. **b-d**, Numerical simulation analyses of the DAL approach with different sample noise levels: (**b**), 5%; (**c**), 10%; (**d**), 20%. Large noise levels in the samples can mislead the initial surrogate model, leading to inappropriate sampling behavior, particularly in the first iteration with a 20% noise level. However, the DAL approach exhibits strong adaptability to noise interference, as it achieves the optimal solution under 5%, 10%, and 20% noise levels quickly after only three iterations. **e-f**, The mean (**e**) and standard deviation (**f**) of optimized objective functions by DAL and GP-AL approaches under different noise levels. The results indicate that the DAL approach exhibits superior resistance to noise interference than the GP-AL method, highlighting the robustness and adaptability of the proposed DAL approach. A more detailed discussion can be found in **Supplementary Note 2**.

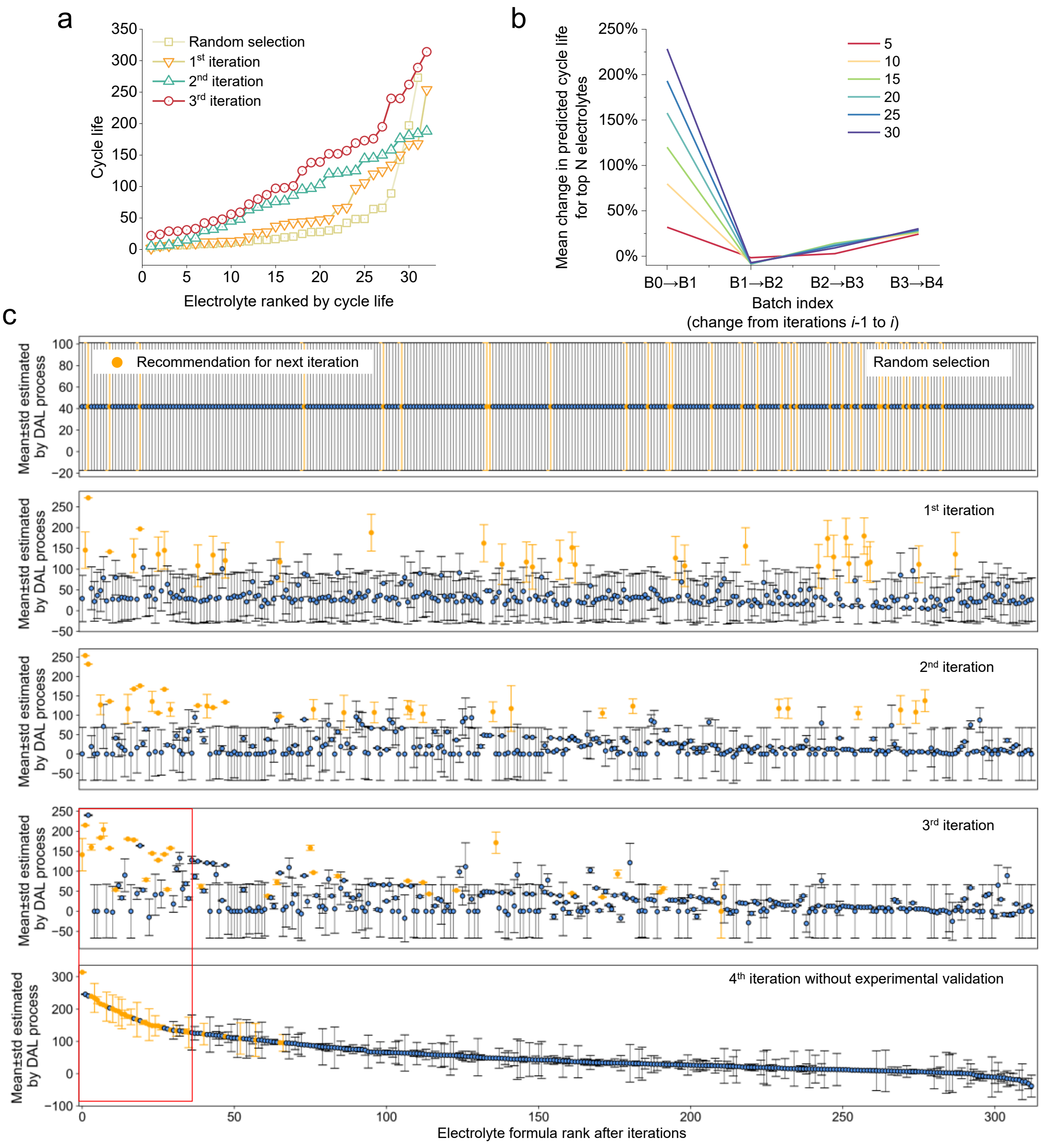

Supplementary Fig. 5 | The results of iterations and termination analysis for the DAL optimization process performed in this study. a, The experimental results of cells' cycle life of measured electrolytes in the initial random selection round and the three iterations, ranked by the cells' cycle life. The data points shown here are the same as those shown in **Figs. 3a, b** (which are plotted differently). We can clearly observe that overall, the cells' cycle life exhibits a continuous improvement over the three iterations, particularly for the range with cell cycle life of over 100 cycles. The illustration here corroborates the results shown in **Figs. 3a, b**. **b**, The relative change (%) of the mean of estimated cells' cycle life (including top *N* data points) between consecutive iterations, wherein the differently colored lines are calculated based on the data of the top *N* longest cell cycle life (*N*= 5, 10, 15, 20, 25, 30). Clearly, as the iteration advances, the change of cells' cycle life between iterations progressively diminishes, highlighting the gradually optimized performance by DAL. **c**, The mean and standard deviation (mean ± std) of the predicted cells' cycle life for each electrolyte after each iteration, ranked by average of the cycle life (not by the numbering of electrolytes). Note that no experimental validation was performed for the 4th iteration. The electrolyte formula rank after each iteration displays predicted cell cycle life of each electrolyte (electrolytes with longer life are listed on the left side of the figure and those with shorter life on the right). The blue points represent predicted electrolytes and their performances in the current iteration, and the orang points represent the electrolytes that are to be validated for the next iteration. As the iteration advances, we can see that the points of evaluation for next iteration (yellow points) in general shift towards the left, highlighting the DAL's ability to identify parameter space regions of electrolytes with long cell cycle life. Further, there is a large overlap between evaluation points of the fourth and third iterations (orang points highlighted in the red boxed area with relatively longer predicted cell cycle life). In contrast, for the blue points highlighted in the red box area, the cell cycle life exhibits a higher uncertainty or relatively higher mean values in the third iteration, indicating a call for balance between exploration and exploitation.

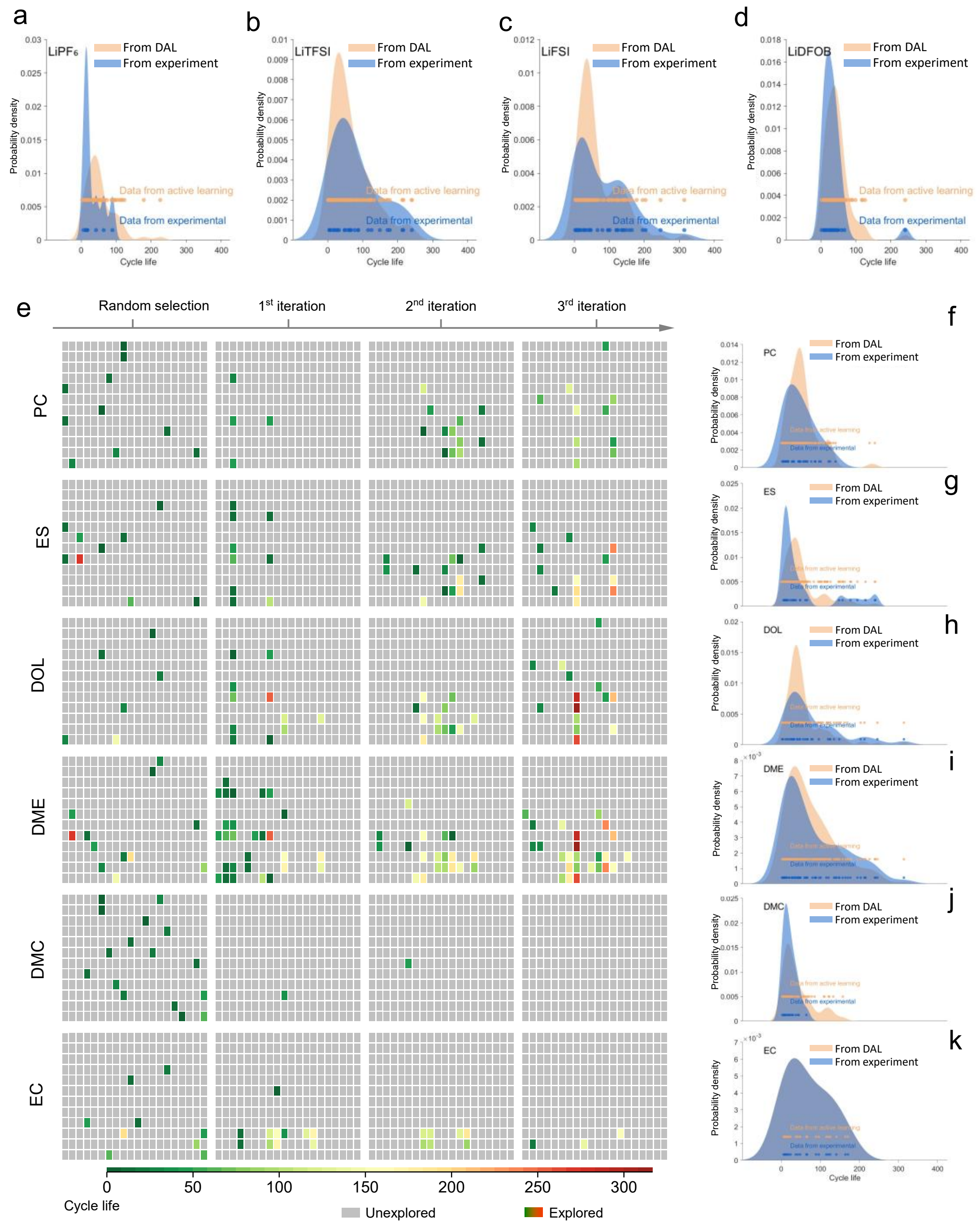

Supplementary Fig. 6 | Kernel density estimation results on exploration of the lithium salt and solvent respectively over iterations. a-d, Kernel density estimation of experimental and DAL-predicted results for exploration of a specific component, herein, the lithium salt. The plots describe the probability density (shaded areas) for all relevant data as a function of the cell cycle life (the colored solid dots represent the exact data points). In general, the distribution of cell cycle life is relatively narrow at <50 cycles indicating low uncertainty for low-cycle-life data. In contrast, the region with cell cycle life of >100 cycles shows a successful matching between the DAL-predicted and experimental results. The experimental and predicted results demonstrate good agreement for all four lithium salts. It is further very clear that LiTFSI and LiFSI show a higher potential for composing long-cycle-life electrolyte formulas compared to $LiPF_6$ and LiDFOB, if for example we are to choose a particular lithium salt. **e**, The visualization of the DAL iterations as shown by the evolution of distribution of tested electrolytes and the cells' cycle life (color coded as shown in the legend) as iteration advances, which is herein representatively grouped by the type of solvents for a clear visualization. **f-k**, Kernel density estimation of experimental and DAL-predicted results for exploration of a specific component, herein, the solvent. The plots describe the probability density (shaded areas) for all relevant data as a function of the cell cycle life (the colored solid dots represent the exact data points). We can see that as DMC was gradually excluded from experimental verification during the iterations (shown in **e**), resulting in very low distribution over the region of cycle life of >100 cycles. In contrast, DME and DOL, continuously verified experimentally during iterations, exhibit a high probability density over the region of cycle life of >100 cycles. Also, EC exhibits a higher probability density over the region of cycle life of >100 cycles. Similar analyses were conducted in terms of the salt concentration and additive to demonstrate the efficiency of DAL for the optimization of LMB electrolytes (see **Supplementary Note 3** for details).

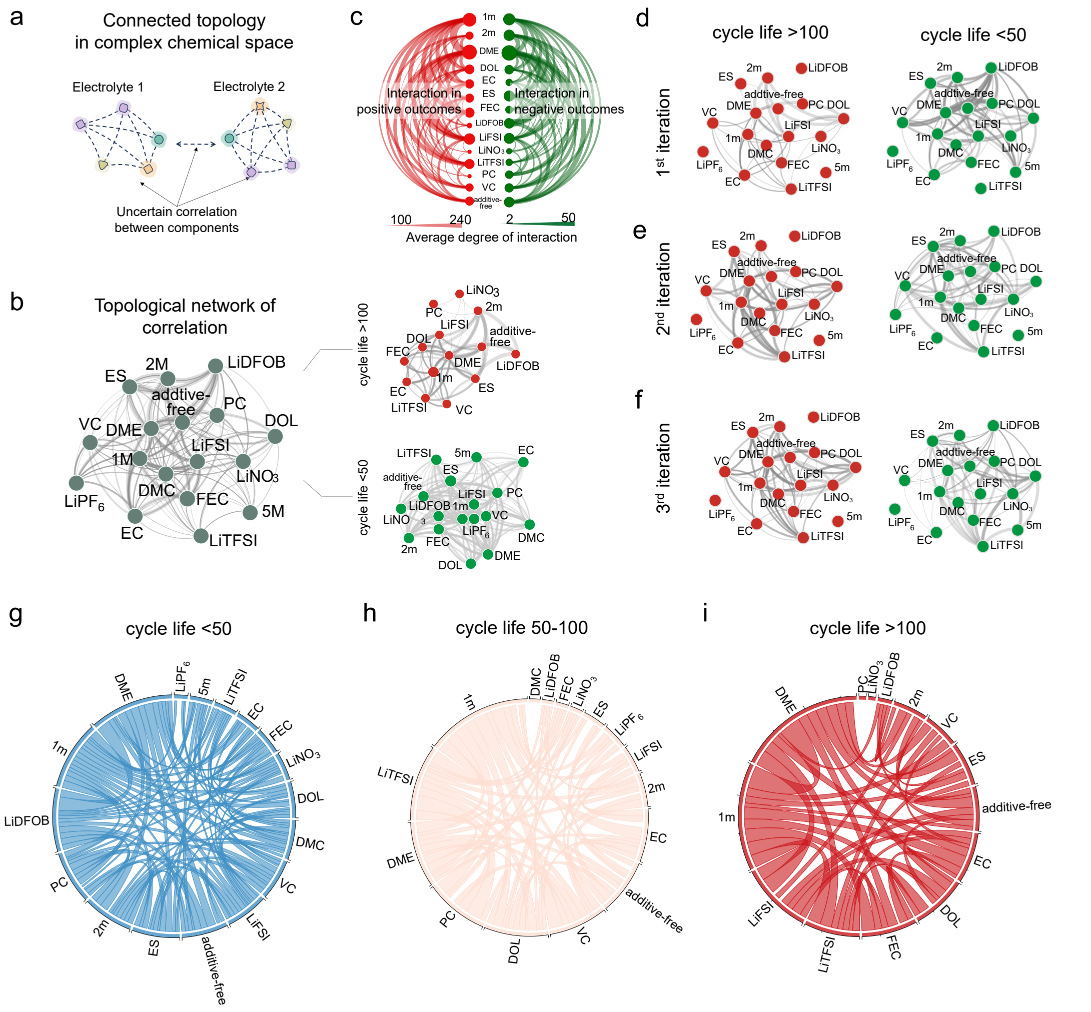

Supplementary Fig. 7 | The conventional manual-based quantification of two-dimensional topological network of components for the LMB electrolytes. a, Schematic showing of the complex topological network of the LMB electrolyte components. The complex systems with multiple parameters can contain substantial unknown inter-component correlations. The lack of fundamental understanding of the complex system of LMB electrolytes hinders effective design of new electrolytes using conventional variable-control experimental methods that is essentially manual-based. **b**, Topological network expressions showing the connection between two components of electrolyte space regarding the battery cycle life (two-dimensional correlations), which are plotted in two parts, one with negative outcomes (green, cycle life <50 cycles) or positive outcomes (red, cycle life >100 cycles) based on experimental data in the random selection. The thickness of the lines between two components represents the degree of interaction (thickness coded shown below), as calculated by averaging the battery cycle life with all electrolytes that contain the two components. **c**, Arc diagram of two-dimensional correlations (pairwise, between two components) that were calculated and present in both the negative outcome and positive outcome. When analyzing the topological network of two-dimensional pairwise correlations, there can be substantial disagreement between the positive and negative messages on one specific correlation (that is, disagreement between the green and red lines for one specific pair of components), making it hard to extract valuable information. **d-f**, The two-dimensional topological network expressions during iterations showing the connection between two components regarding the cell cycle life (i.e. two-dimensional correlations), which are plotted in two separate parts, one with negative outcomes (green, cell cycle life <50 cycles) and one with positive outcomes (red, cell cycle life >100 cycles). The average degree of interaction (ADI) between two components were calculated by averaging the cell cycle life with all electrolytes that contain the two components, and are represented by the thickness of the lines between two components (thickness coding scheme shown at the bottom of each network). **g-i**, Chord diagrams (specific gravity distribution) showing the two-dimensional component correlations using the cell cycle life data over the three DAL iterations; the electrolytes are divided into three groups: <50 cycles, 50-100 cycles, and >100 cycles based on the measured cell cycle life. Manual-based extraction of correlations between two components can lead to contradictions; for example, there is strong DME-ES pairwise correlation simultaneously observed in the group of <50 cycles (negative outcome) and group of >100 cycles (positive outcome). This occurs primarily because other components could make this DME-ES couple perform either better or worse. In this case, it would be impossible to holistically assess this pairwise correlation using manual methods. Apparently, by comparing the network of positive outcome with the negative of the same iteration, we can see that they reveal fundamentally very similar topological patterns, which makes it practically impossible to estimate the truth of correlations between two components (also shown in **Supplementary Fig. 7c**).

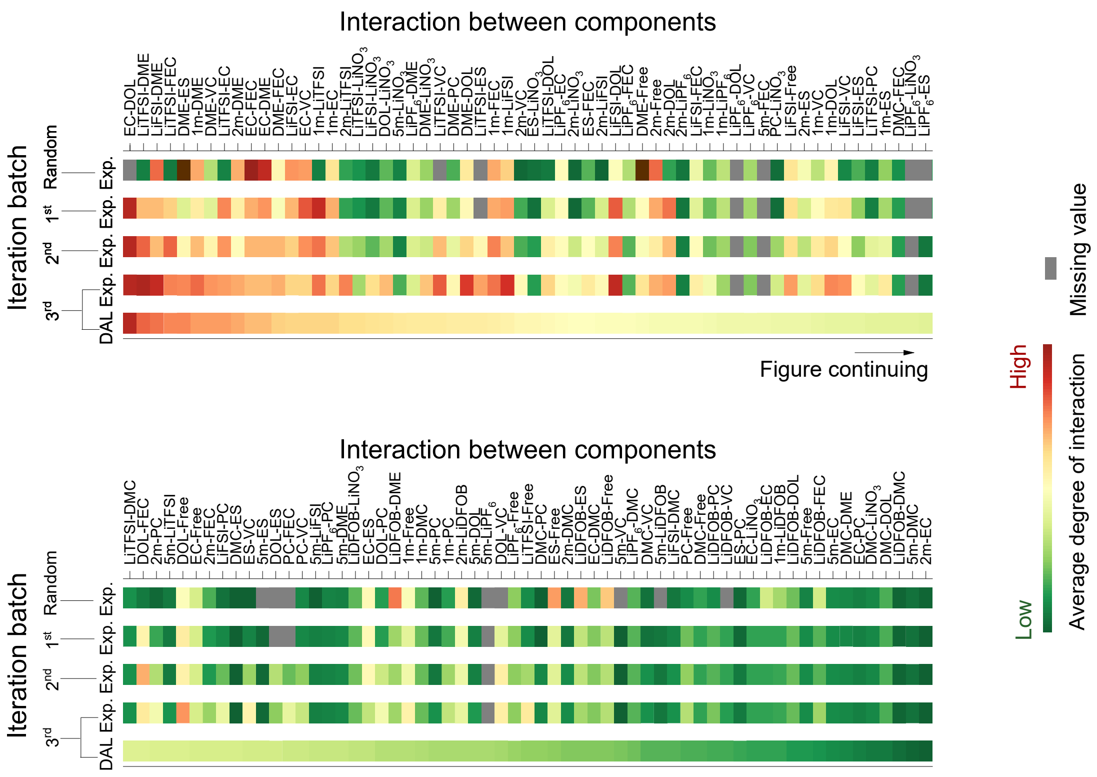

Supplementary Fig. 8 | The evolution of pairwise correlation between components (average degree of interaction, color coded, quantified by TSC) during iterative optimization.

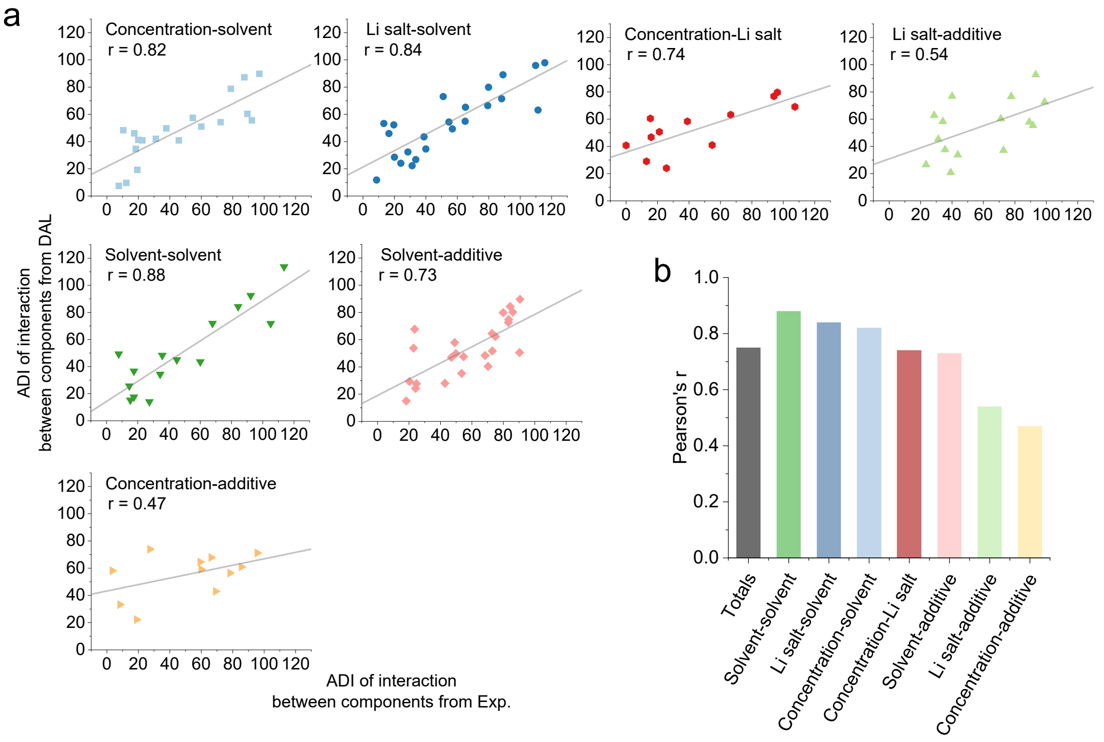

Supplementary Fig. 9 | Pearson correlation analysis between experiment-derived ADI values (horizontal axis) and the DAL-derived ADI values (vertical axis) after the 3rd iteration. a, The detailed Pearson correlation analysis by different interaction type. *r* value is Pearson correlation coefficient. **b**, The Pearson correlation coefficient *r* in different interaction type. The solvent-based correlation values (concentration-solvent, Li salt-solvent, solvent-solvent) are obviously higher than the additive-based correlation value (solvent-additive, Li salt-additive, concentration-additive) (**Supplementary Fig. 9a** top panels vs. bottom panels, and **Supplementary Fig. 9b**). Because the nature of our DAL optimization is to actively hunt for intercomponent correlations that lead to high performance (i.e. long cell lifespan) and to neglect correlations that leads to poor performance, at the end of DAL learning, the model would have a much stronger learning of “better” correlations than “worse” correlations. Therefore, the higher correlation ecoefficiency for solvent-based interaction means the solvent is more significant than additive in terms of coupling with other components for longer LMB lifespan.

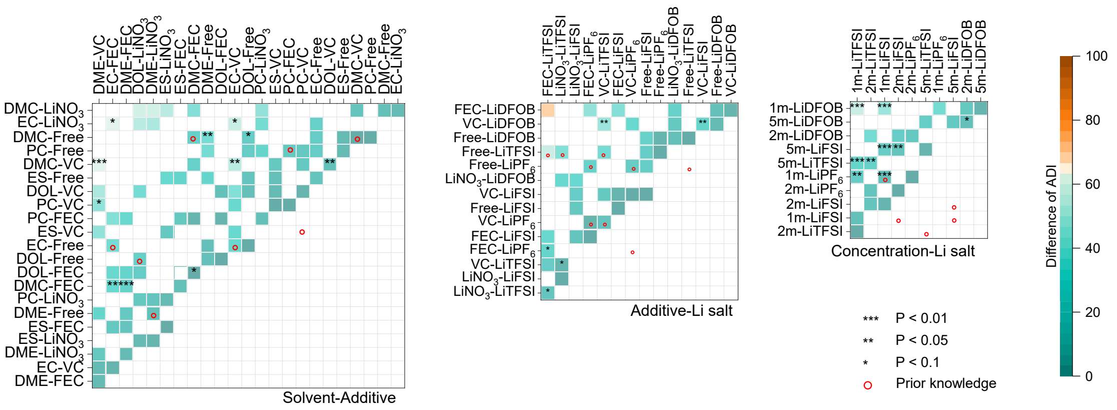

Supplementary Fig. 10 | The detailed mapping of pairwise interactions between two components grouped in solvent-additive, additive-Li salt and concentration-Li salt. Here in these maps, the * shown at each pixel means the significance level of each pair of comparison in hypothesis testing based on the experimental dataset: ***: p<0.01; **: p<0.05; *: p<0.1. The red circles in the mapping represent prior knowledge reported in the literature.

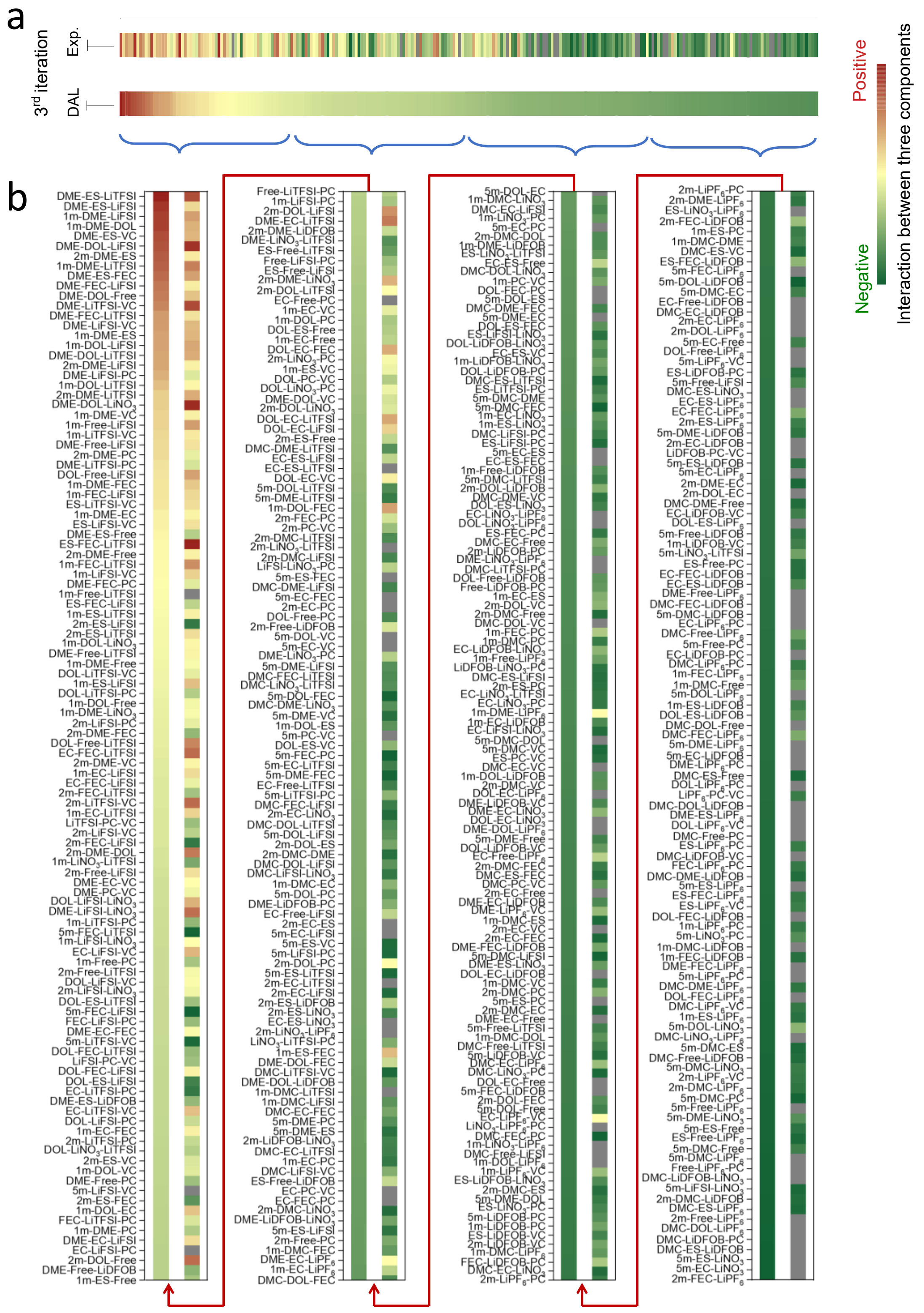

Supplementary Fig. 11 | High-dimensional interaction among three components. a, The quantification based on experimental validation data (denoted as “Exp.” in the diagram), which is compared with that based on DAL’s predictions after 3 iterations. **b**, A detailed segmented illustration of the three-dimensional ADI. The values were calculated by averaging all predicted results involving three components, illustrating the impact of interactions among three components captured by DAL on the battery cycle life.

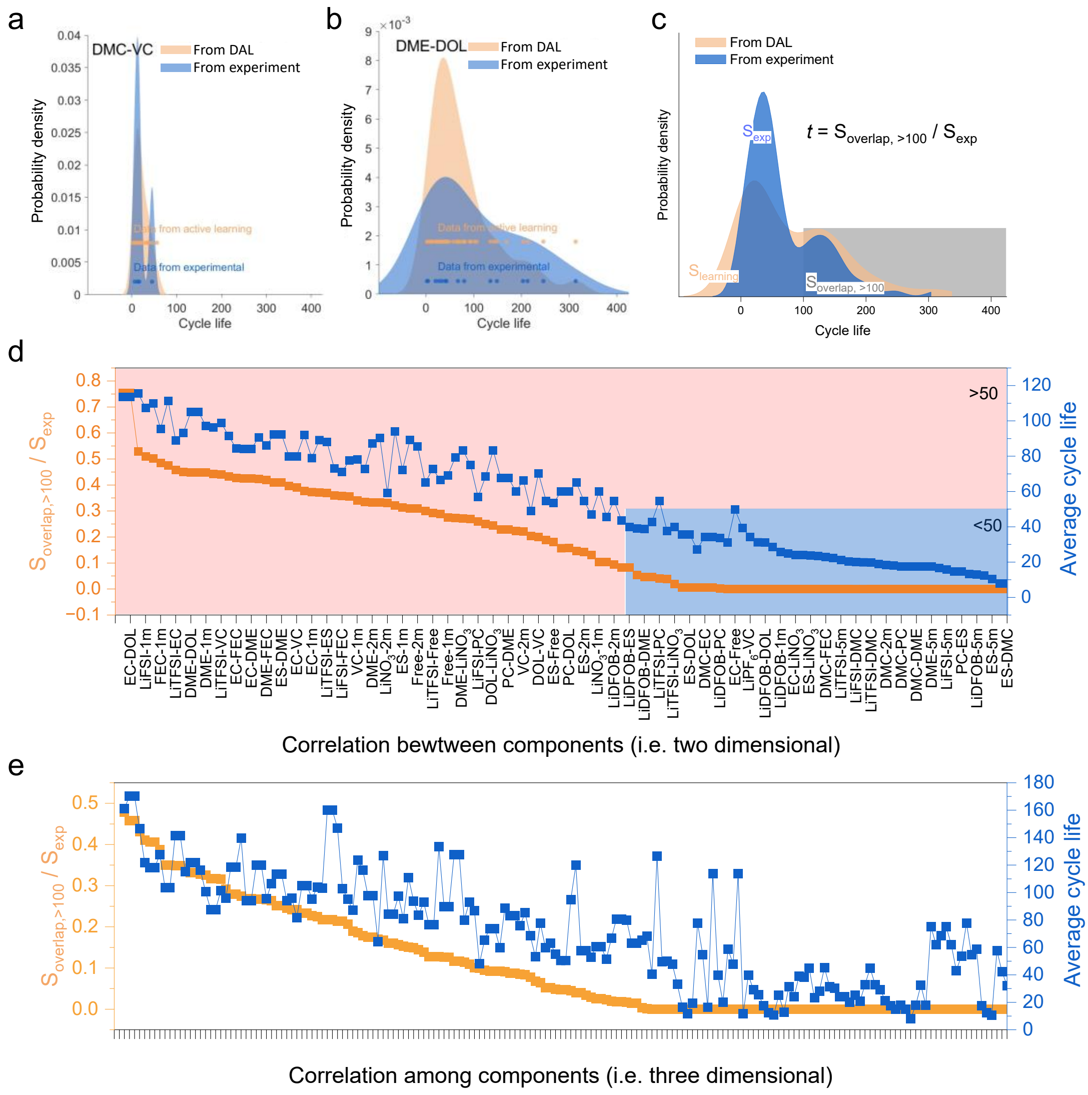

Supplementary Fig. 12 | Electrolyte component correlations captured by the DAL approach. a-c, Kernel density estimation of experimental and DAL-predicted results for exploration of the two-dimensional correlation between two solvents. The plots describe the probability density for all relevant data as a function of the cell cycle life (the colored solid dots represent the exact data points). To assess the accuracy of the DAL approach in capturing two-dimensional component correlations, we employed kernel density estimation (see **Supplementary Fig. 19** for details) for all two-dimensional samples, and two representative pairwise correlations are shown in (**a**) and **(b)**. DMC-VC tend to exhibit a short cell cycle life, while DME-DOL tends to exhibit a long cell cycle life. In order to validate the accuracy of capturing correlation across the entire domain, we introduce the ratio *t*, which is calculated as the overlapping area of the DAL-predicted data distribution relative to the experimental data distribution for the region with cell cycle life greater than 100 cycles (**c**). **d**, The plot of the two-dimensional component correlation ratio *t* along with the average experimental cell cycle life (scales respectively shown on the left and right axes). By observing the trend of *t* and average cell cycle life, we observe that the DAL results are aligned well with the experimental data, indicating that DAL can effectively capture and predict two-dimensional component correlations that contribute to a higher cell cycle life. **e**, The plot of the three-dimensional component correlation ratio *t* along with the average experimental cell cycle life (scales respectively shown on the left and right axes). For clarity, the x-axis labelling of the exact three-components examined is omitted. By observing the trend of *t* and average cell cycle life, we observe that the DAL results are aligned well with the experimental data, indicating the DAL's ability to capture three-dimensional correlations. We therefore have confidence in projecting that the DAL approach can perhaps capture even higher-dimensional correlations among electrolyte components.

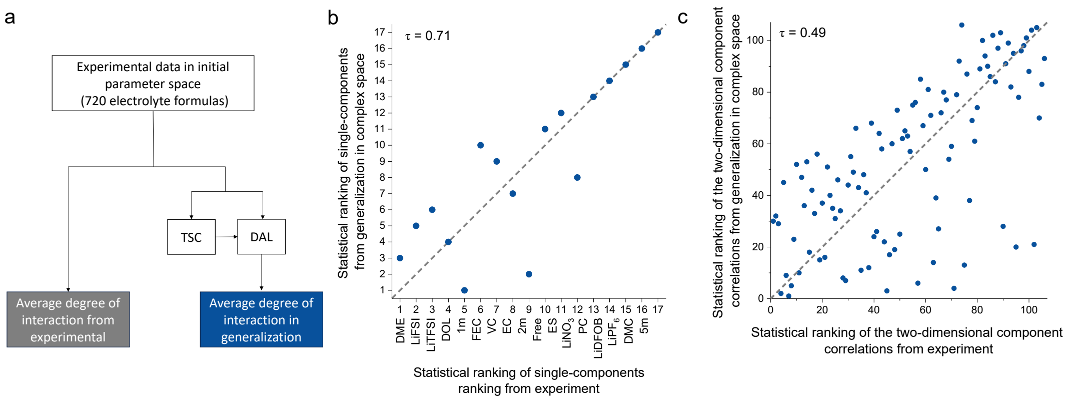

Supplementary Fig. 13 | The information of quantified component correlations generalized for a larger electrolyte space. a, A flow chart illustrating the two methods that we can use to quantify the component correlations from the experimental data from the original parameter space: by a conventional statistical analysis, or by a TSC-DAL analysis. For the latter, TSC and DAL were applied to experimental data from the original parameter space to statistically analyze component correlations. **b**, Comparison of statistical ranking of single-components derived from DAL inference for a larger electrolyte space with the ranking derived from the experimental data. The numbers on the axis represent the ranking for the respective components, and only the top 17 components are shown here. Specifically, the component with a higher ranking corresponds to statistically more likely to result in a long lifespan for an electrolyte containing this component. Comparing the experimental values and the predicted values of specific components allows for the assessment of prediction accuracy against experimental outcomes. The Kendall ranking correlation coefficient between the experimental and the DAL inference is 0.71, indicating that the transformation of electrolyte component correlation information into an expanded parameter space has been successfully learned by TSC and DAL. **c**, Comparison of statistical ranking of the two-dimensional component correlations derived from DAL inference for a larger electrolyte space and those derived from the experimental data. The Kendall ranking correlation coefficient is 0.49, indicating a rather high accuracy. Nevertheless, this is lower than the coefficient for single components (0.71), which is due to the increased complexity as the correlation information expands from one-dimensional to two-dimensional.

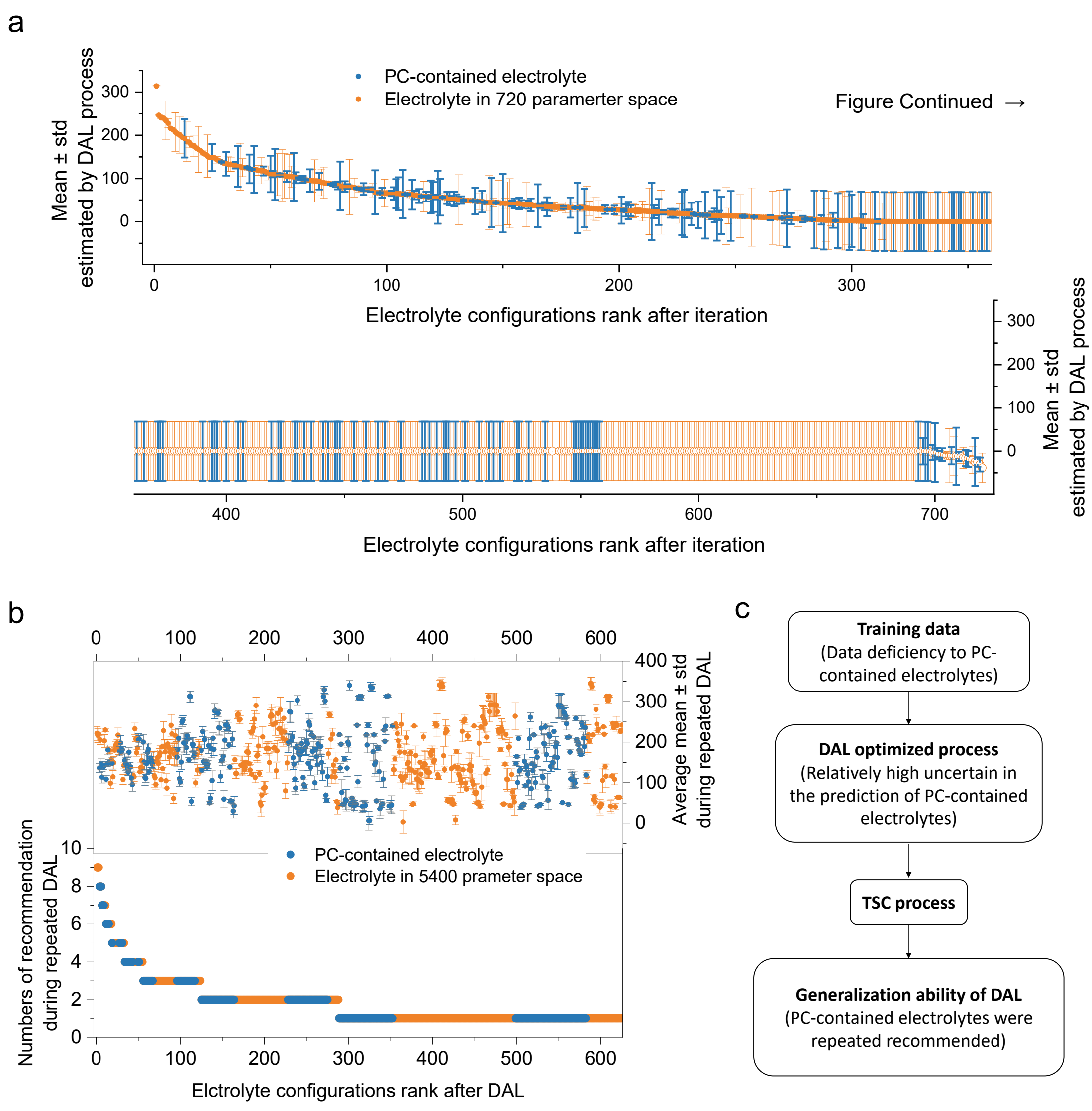

Supplementary Fig. 14 | Validation of PC-containing electrolytes in DAL generalization in a higher-dimensional parameter space. a, Average mean value and standard deviation (mean ± std) of cell cycle life predicted by the DAL in the original parameter space (size of 720); the predicted cell cycle life for PC-contained electrolytes is shown in blue, from which we can clearly see high uncertainty as indicated by the high standard deviation. **b**, Average value and standard deviation (mean ± std) of cell cycle life for electrolytes repeatedly recommended by Thompson sampling of DAL during the optimization in the higher-dimensional parameter space of size 5400 (top of the figure), along with the corresponding number of recommendations (bottom of the figure). We can see that the PC-containing electrolytes were persistently recommended for experimental validation in the next batch. **c**, The flow chart illustrating the mechanism for repeated recommendation of PC-containing electrolytes for validation to increase prediction accuracy. The repeated recommendation is owed to high experimental errors displayed by the PC-contained electrolytes which show high uncertainty.

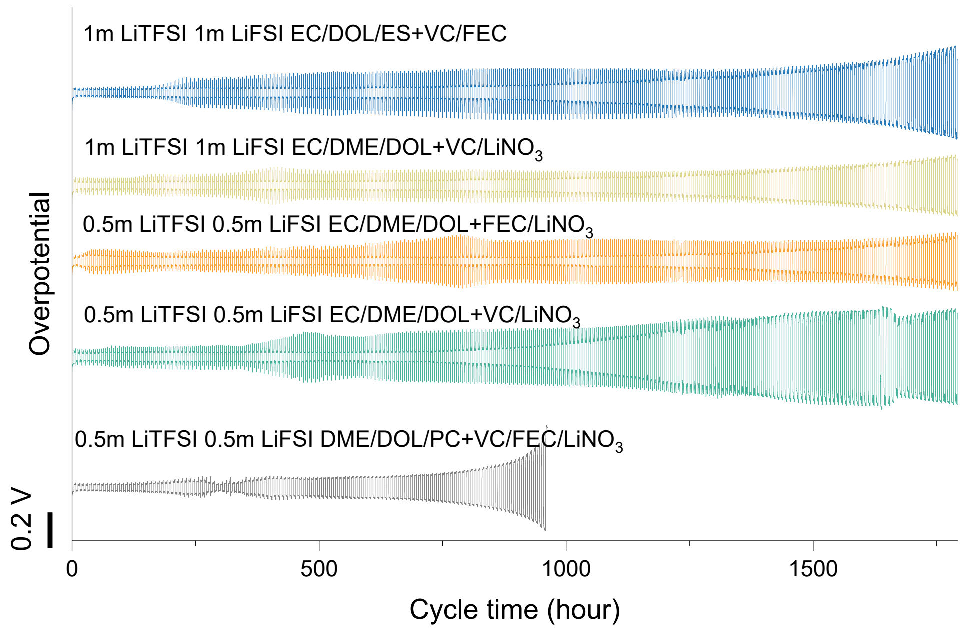

Supplementary Fig. 15 | The Li plating/striping voltage curves for validation of the mostly recommended five electrolyte formulas during the zero-shot optimization in the expanded parameter space.

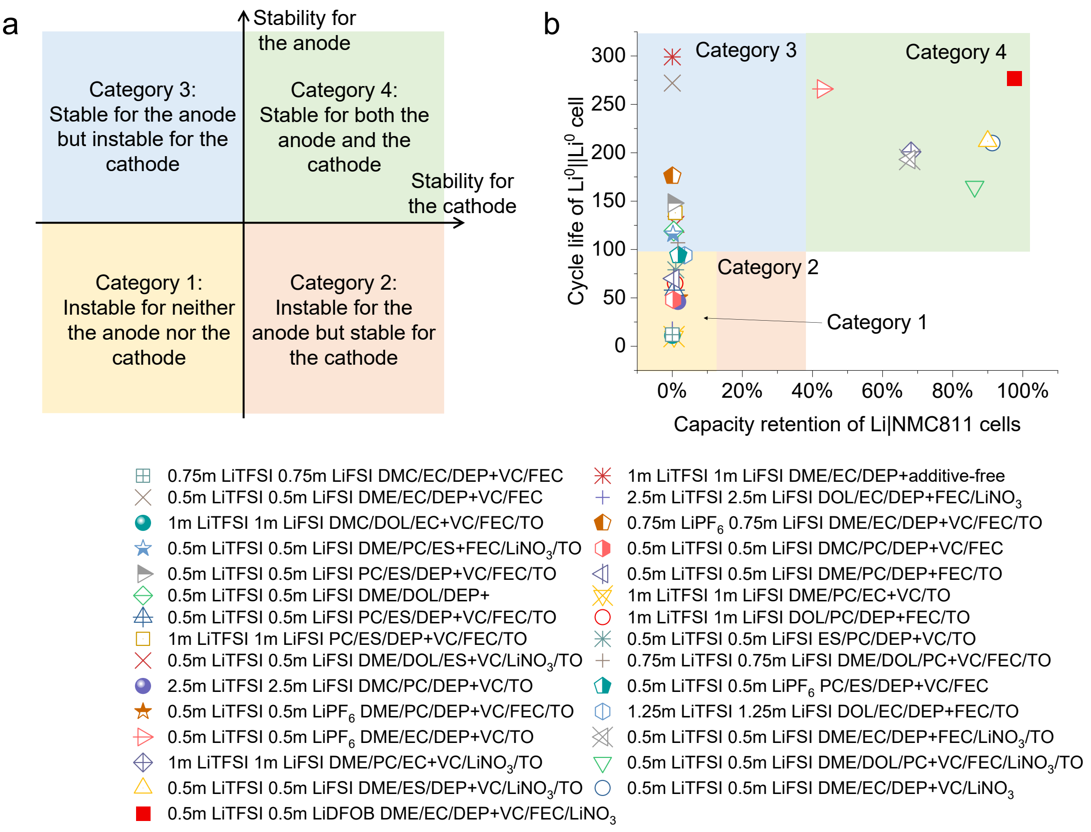

Supplementary Fig. 16 | Experimental validation of the relation between anode stability and full cell stability. a, Conceptual subcategories of electrolytes based on the different stability towards the anode and cathode. **b**, Experimental data for validation of the relation. The stability of anode is represented by the $Li^0|Li^0$ cell cycle life, where long cycle life (qualitatively defined as >100 cycles) is used to indicate anode stability. The color-coded categories in (**b**) correspond to the illustration in (**a**), serving as a qualitative guide without implying quantitative division.

We can categorize all electrolytes as follows: 1) instable for neither the anode nor the cathode, 2) instable for the anode but stable for the cathode, 3) stable for the anode but instable for the cathode, 4) stable for both the anode and the cathode, which is illustrated in **Supplementary Fig. 16a**. The rationale for optimizing Li^0|LMO full cell electrolytes is to find the electrolytes in category 4 (stable for both electrodes) by incorporating the knowledge learned from the two DAL optimizations (the first three $Li^0|Li^0$ cell iterations, and the one Li^0|LMO iteration).

To visualize this, in **Supplementary Fig. 16b** we plotted the performance data ($Li^0|Li^0$ cell cycle life and Li^0|LMO capacity retention) of the experimentally tested electrolytes (in the newly constructed parameter space that incorporates two new molecules, 1,2-diethoxypropane (DEP) and trioxane (TO)). We can see that the electrolytes that support high full-cell stability also exhibits long $Li^0|Li^0$ cell cycle life (Category 4). Conversely, electrolytes with short $Li^0|Li^0$ cell cycle life often leads to low Li^0|LMO full-cell capacity retention (Category 1). There are a few electrolytes that support long $Li^0|Li^0$ cell cycle life but rather low Li^0|LMO full-cell capacity retention (Category 3). Therefore, LMA stability is crucial for achieving long cycle life in full cells.

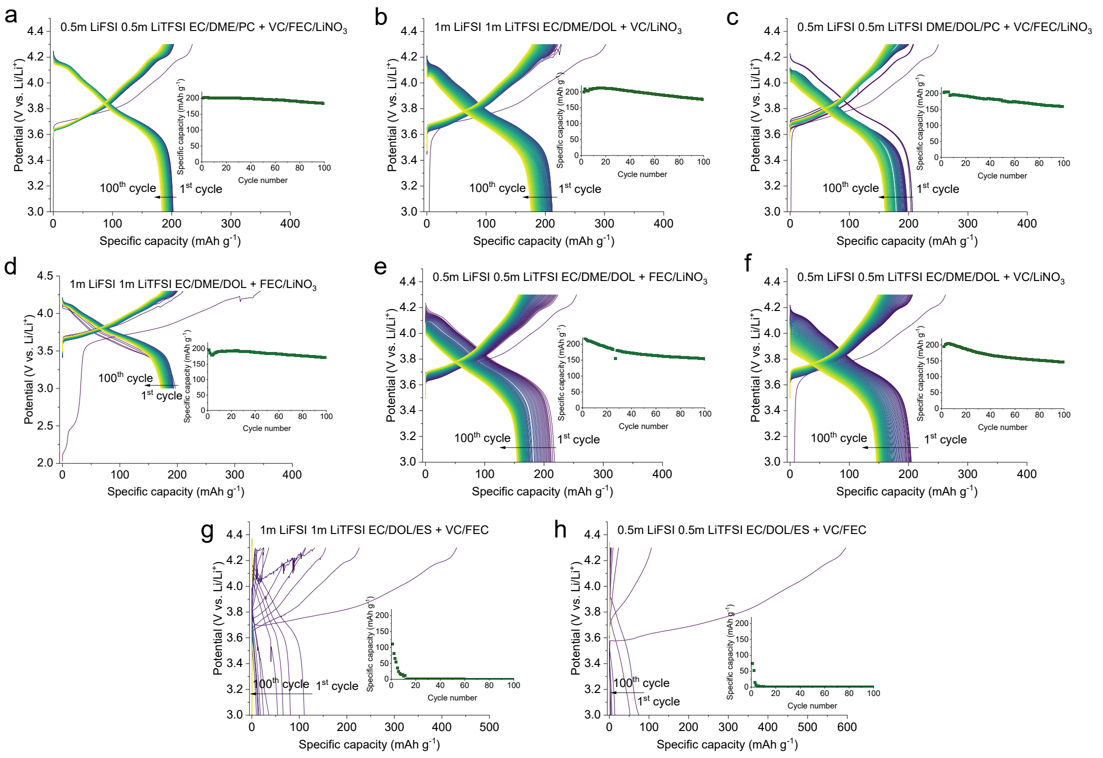

Supplementary Fig. 17 | Detailed voltage profiles for Li^0|NCM811 full cells using the top 8 electrolytes: (**a**) 0.5m LiFSI 0.5m LiTFSI EC/DME/PC+VC/FEC/$LiNO_3$; (**b**) 1m LiFSI 1m LiTFSI EC/DME/DOL+VC/$LiNO_3$; (**c**) 0.5m LiFSI 0.5m LiTFSI DME/DOL/PC+VC/FEC/$LiNO_3$; (**d**) 1m LiFSI 1m LiTFSI EC/DME/DOL+FEC/$LiNO_3$; (**e**) 0.5m LiFSI 0.5m LiTFSI EC/DME/DOL+FEC/$LiNO_3$; (**f**) 0.5m LiFSI 0.5m LiTFSI EC/DME/DOL+VC/$LiNO_3$; (**g**) 1m LiFSI 1m LiTFSI EC/DOL/ES+VC/FEC; (**h**) 0.5m LiFSI 0.5m LiTFSI EC/DOL/ES+VC/FEC. Inset: corresponding capacity during cycling for each electrolyte.

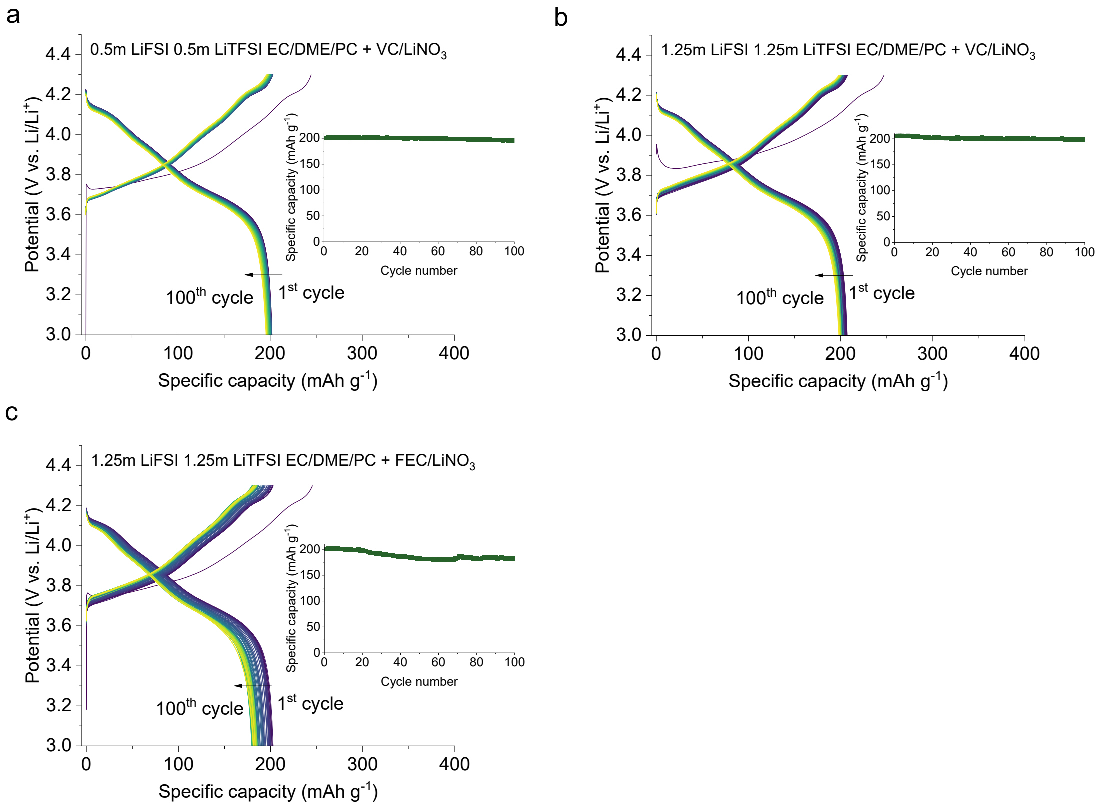

Supplementary Fig. 18 | Detailed voltage profiles for Li^0|NCM811 full cells using the DAL optimized electrolytes: (**a**) 0.5m LiFSI 0.5m LiTFSI EC/DME/PC+VC/$LiNO_3$; (**b**) 1.25m LiFSI 1.25m LiTFSI EC/DME/PC+VC/$LiNO_3$; (**c**) 1.25m LiFSI 1.25m LiTFSI EC/DME/PC+FEC/$LiNO_3$; Inset: corresponding capacity during cycling for each electrolyte.

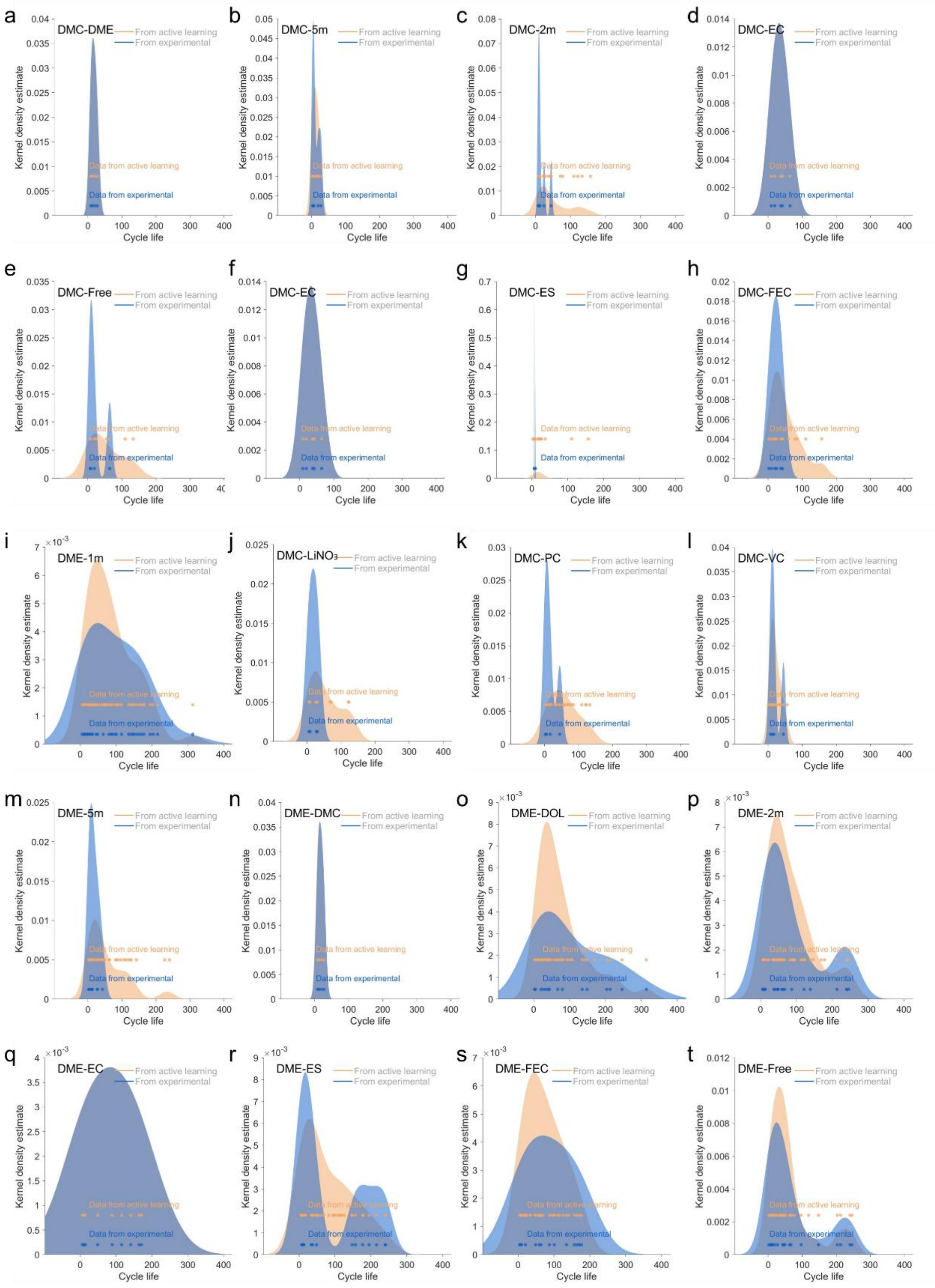

Figure continued →

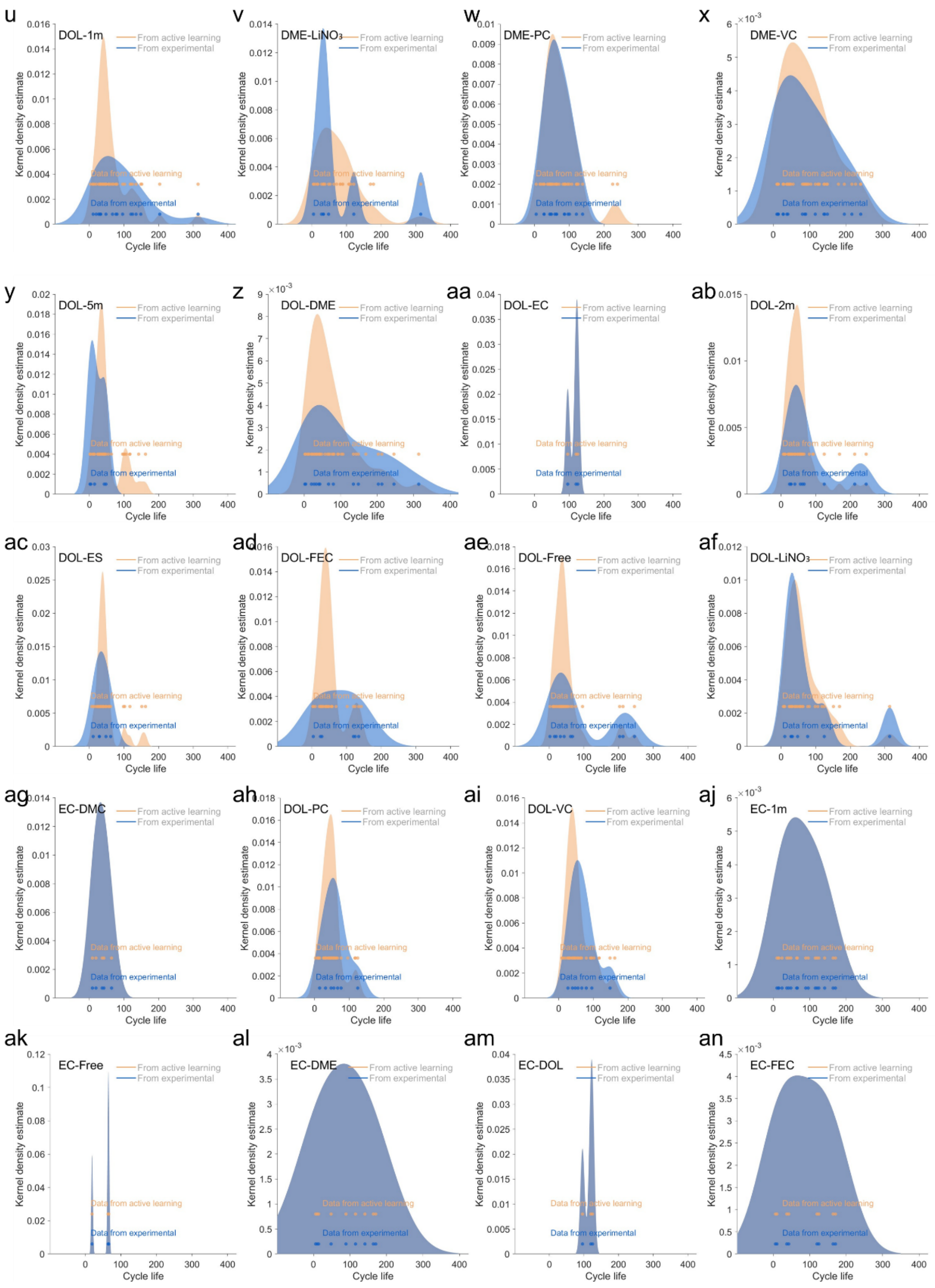

Figure continued →

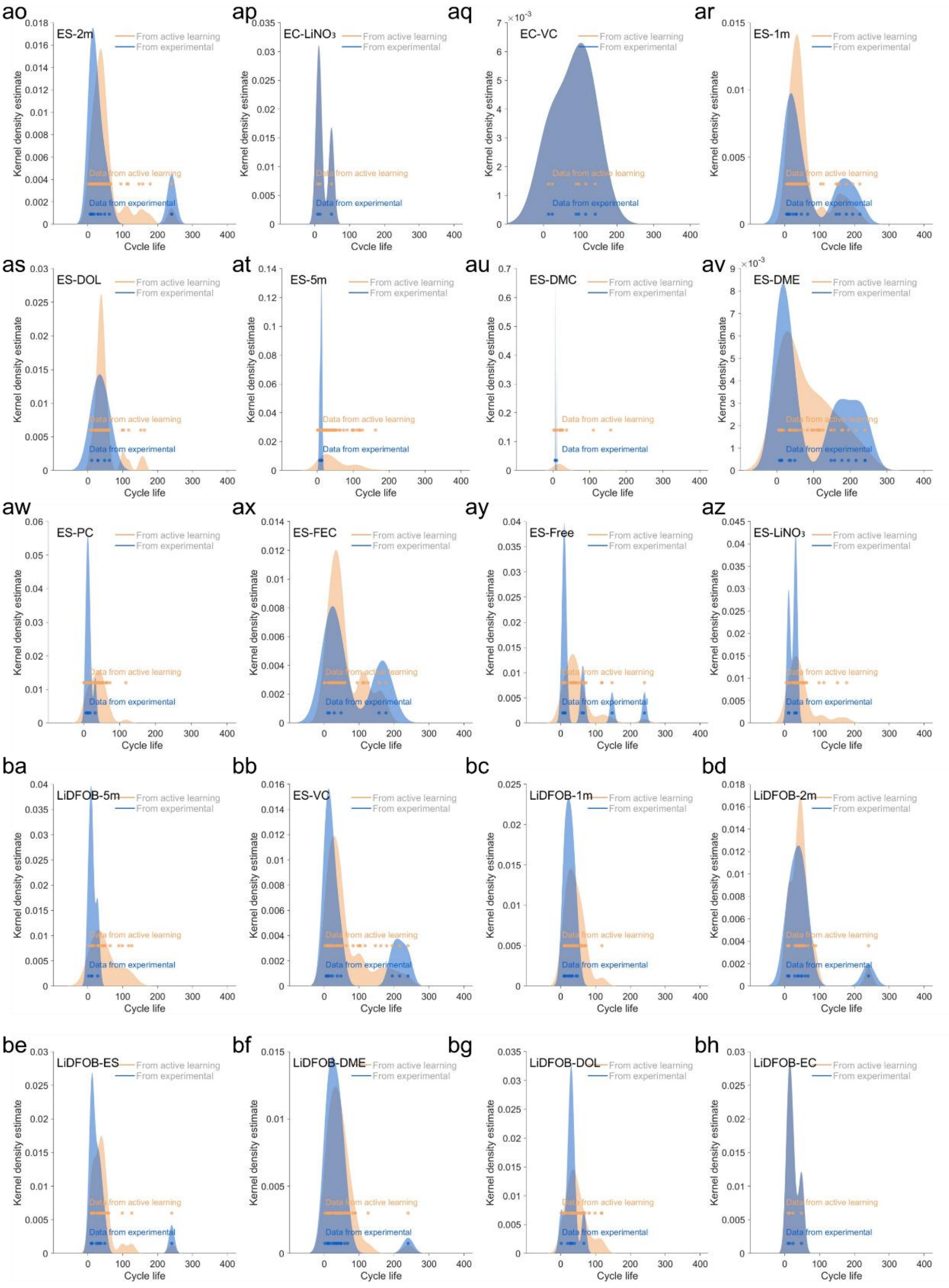

Figure continued →

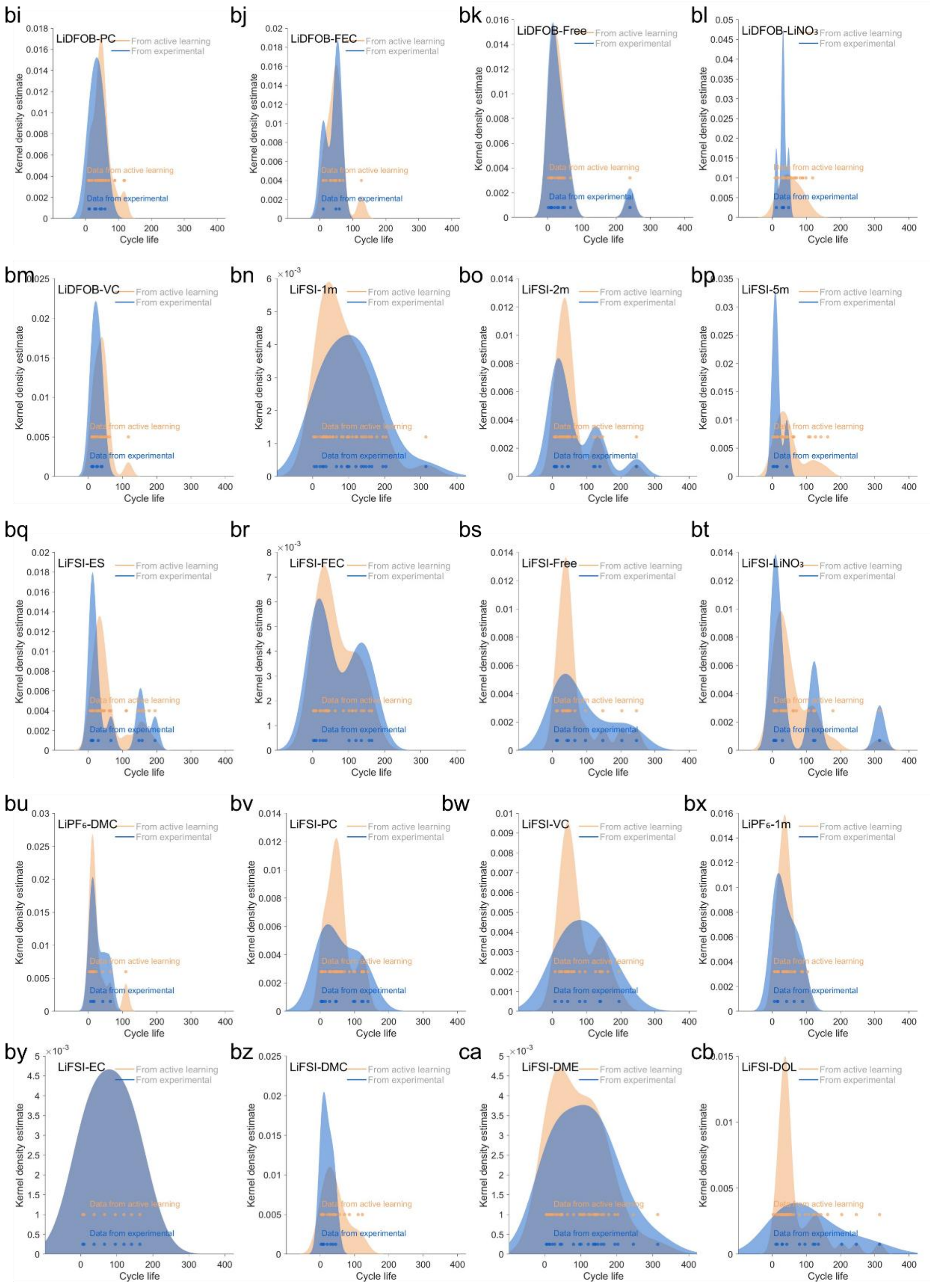

Figure continued →

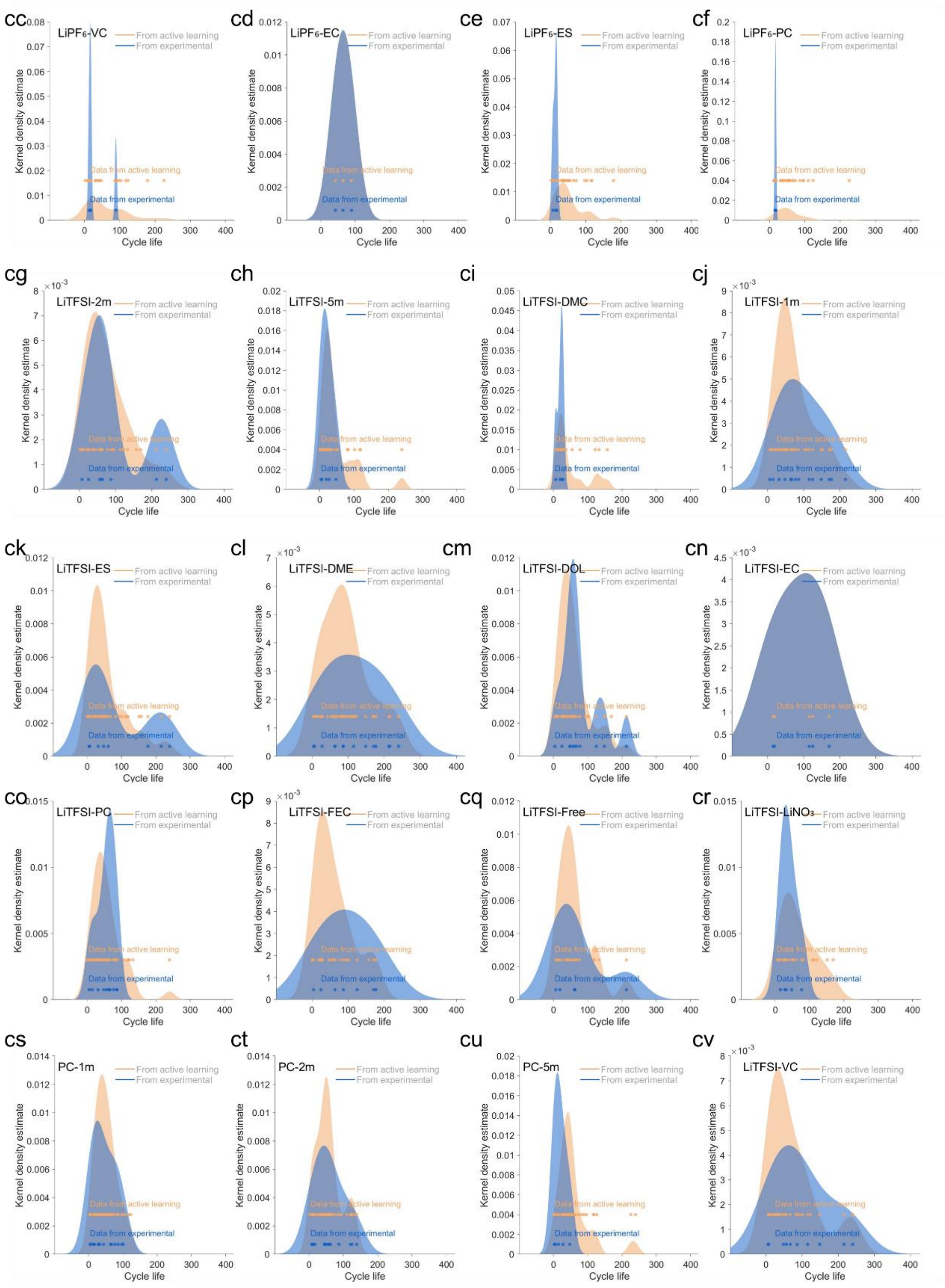

Figure continued →

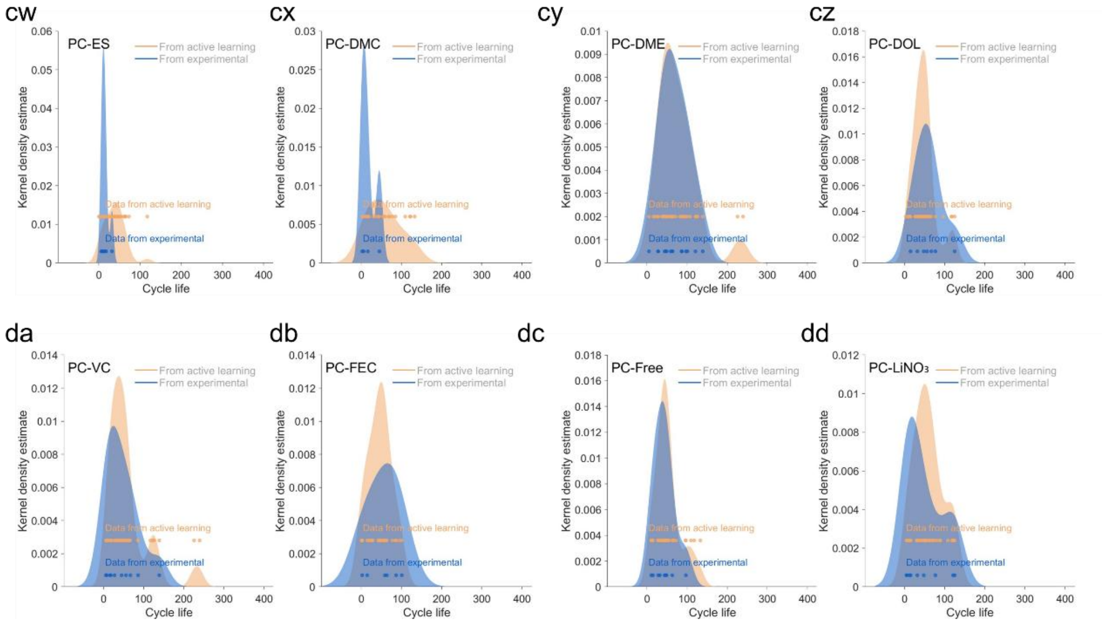

Supplementary Fig. 19 | Kernel density estimation of experimental and DAL training results for interaction between components: (**a**) DMC–DME; (**b**) DMC–5 m; (**c**) DMC–2 m; (**d**) DMC–EC; (**e**) DMC–Free; (**f**) DMC–EC; (**g**) DMC–ES; (**h**) DMC–FEC; (**i**) DME–1 m; (**j**) DMC–LiNO$_3$; (**k**) DMC–PC; (**l**) DMC–VC; (**m**) DMC–DOL; (**n**) DME–DMC; (**o**) DME–DOL; (**p**) DME–2 m; (**q**) DME–EC; (**r**) DME–ES; (**s**) DME–FEC; (**t**) DME–Free; (**u**) DOL–1 m; (**v**) DME–LiNO$_3$; (**w**) DME–PC; (**x**) DME–VC; (**y**) DOL–5 m; (**z**) DOL–DME; (**aa**) DOL–EC; (**ab**) DOL–2 m; (**ac**) DOL–ES; (**ad**) DOL–FEC; (**ae**) DOL–Free; (**af**) DOL–LiNO$_3$; (**ag**) EC–DMC; (**ah**) DOL–PC; (**ai**) DOL–VC; (**aj**) EC–1 m; (**ak**) EC–Free; (**al**) EC–DME; (**am**) EC–DOL; (**an**) EC–FEC; (**ao**) ES–2 m; (**ap**) EC–LiNO$_3$; (**aq**) EC–VC; (**ar**) ES–1 m; (**as**) ES–DOL; (**at**) ES–5 m; (**au**) ES–DMC; (**av**) ES–DME; (**aw**) ES–PC; (**ax**) ES–FEC; (**ay**) ES–Free; (**az**) ES–LiNO$_3$; (**ba**) LiDFOB–5 m; (**bb**) ES–VC; (**bc**) LiDFOB–1 m; (**bd**) LiDFOB–2 m; (**be**) LiDFOB–ES; (**bf**) LiDFOB–DME; (**bg**) LiDFOB–DOL; (**bh**) LiDFOB–EC; (**bi**) LiDFOB–PC; (**bj**) LiDFOB–FEC; (**bk**) LiDFOB–Free; (**bl**) LiDFOB–LiNO$_3$; (**bm**) LiDFOB–VC; (**bn**) LiFSI–1 m; (**bo**) LiFSI–2 m; (**bp**) LiFSI–5 m; (**bq**) LiFSI–ES; (**br**) LiFSI–FEC; (**bs**) LiFSI–Free; (**bt**) LiFSI–LiNO$_3$; (**bu**) LiPF6–DMC; (**bv**) LiFSI–PC; (**bw**) LiFSI–VC; (**bx**) LiPF$_6$–1 m; (**by**) LiFSI–EC; (**bz**) LiFSI–DMC; (**ca**) LiFSI–DME; (**cb**) LiFSI–DOL; (**cc**) LiPF$_6$–VC; (**cd**) LiPF$_6$–EC; (**ce**) LiPF$_6$–ES; (**cf**) LiPF$_6$–PC; (**cg**) LiTFSI–2 m; (**ch**) LiTFSI–5 m; (**ci**) LiTFSI–DMC; (**cj**) LiTFSI–1 m; (**ck**) LiTFSI–ES; (**cl**) LiTFSI–DME; (**cm**) LiTFSI–DOL; (**cn**) LiTFSI–EC; (**co**) LiTFSI–PC;

(**cp**) LiTFSI–FEC; (**cq**) LiTFSI–Free; (**cr**) LiTFSI–$LiNO_3$; (**cs**) PC–1 m; (**ct**) PC–2 m; (**cu**) PC–5 m; (**cv**) LiTFSI–VC; (**cw**) PC–ES; (**cx**) PC–DMC; (**cy**) PC–DME; (**cz**) PC–DOL; (**da**) PC–VC; (**db**) PC–FEC; (**dc**) PC–Free; (**dd**) PC–$LiNO_3$.

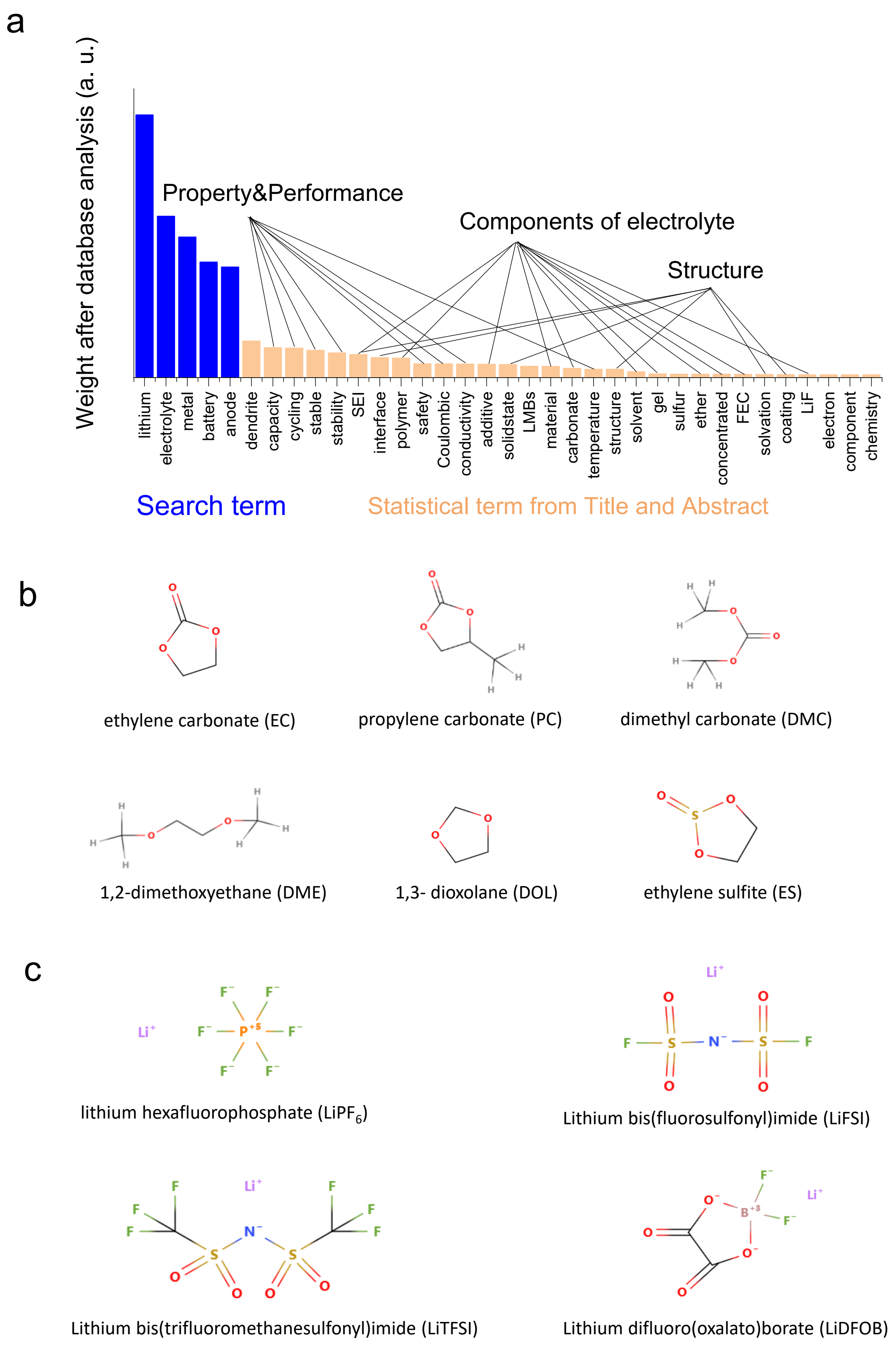

Supplementary Fig. 20 | The selection of components for composing the electrolyte chemical space. a, The results of statistical analysis of the vocabulary in the database. **b**, Solvents and their chemical structures in the electrolyte parameter space. **c**, Lithium salts and their chemical structures in the electrolyte parameter space.

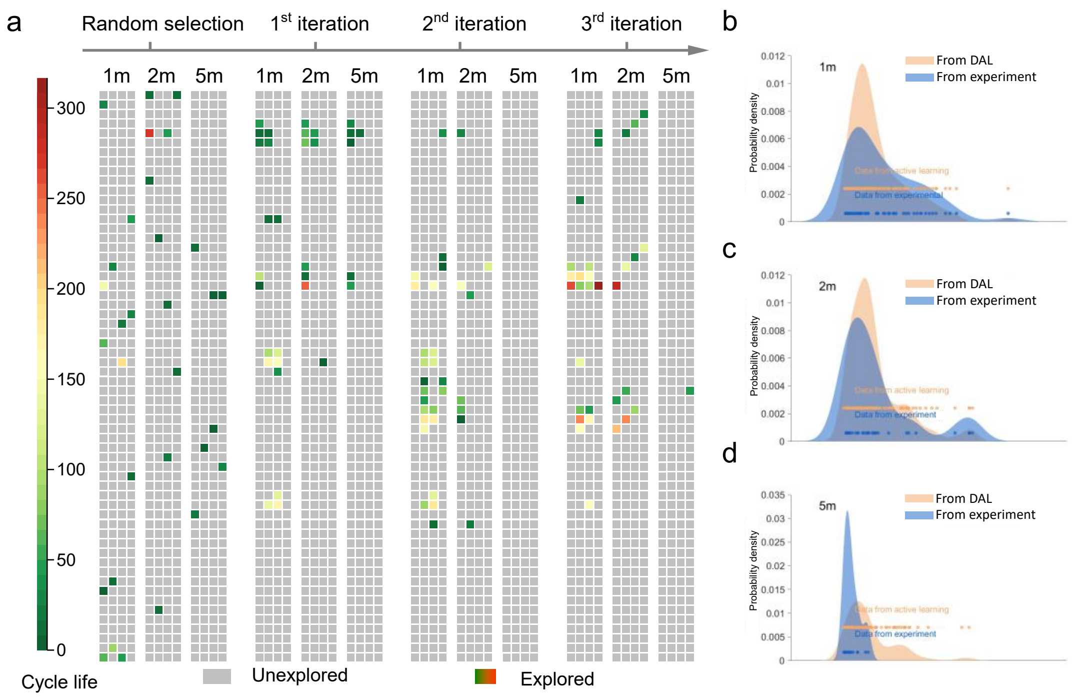

Supplementary Fig. 21 | a, The visualization of the DAL iterations as shown by the evolution of distribution of tested electrolytes and the cells' cycle life (color coded as shown in the legend) as iteration advances, which is herein representatively grouped by the type of salt concentrations for a clear visualization. **b-d**, Kernel density estimation of experimental and DAL results for the cycle life based on salt concentration.

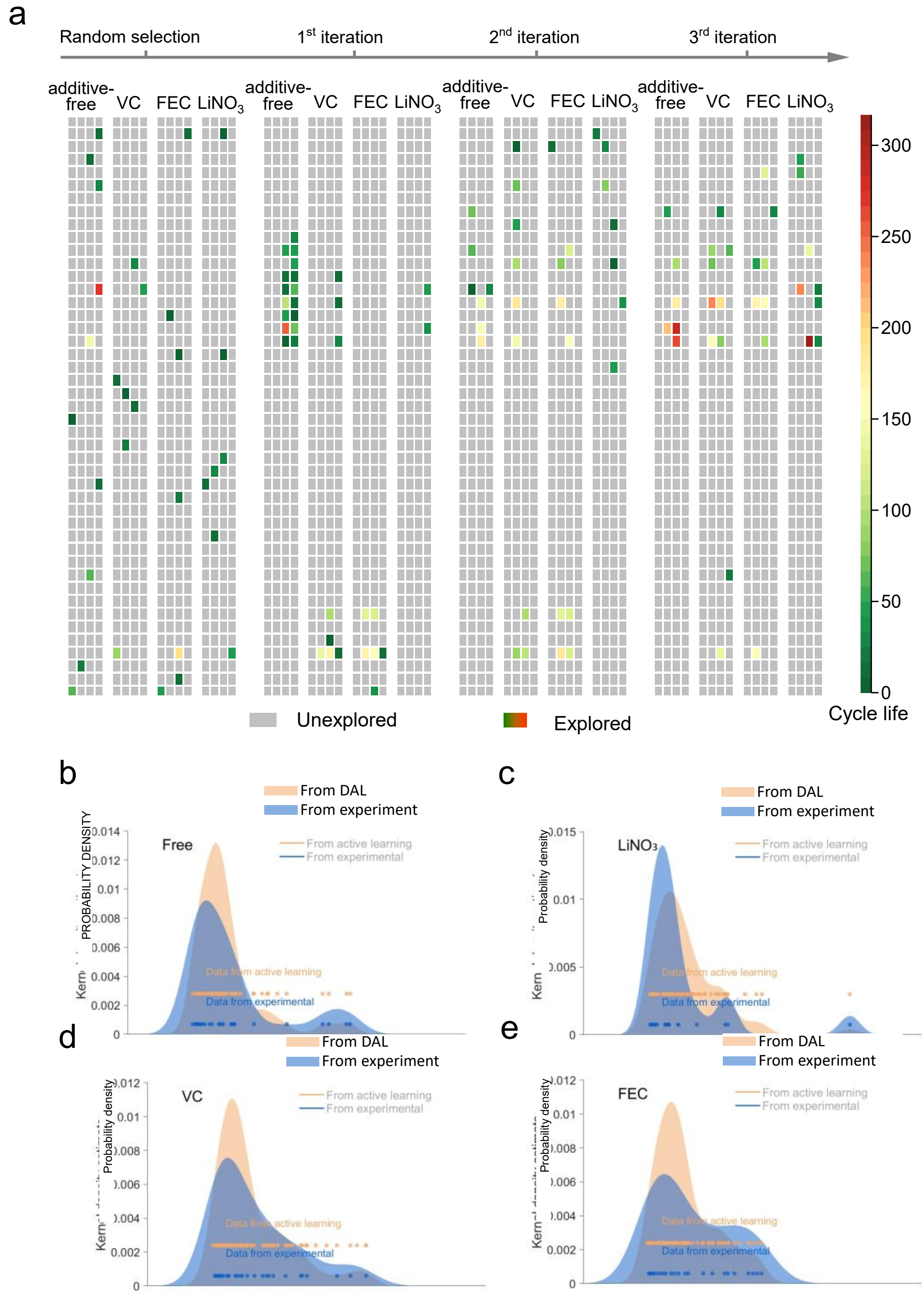

Supplementary Fig. 22 | a, The visualization of the DAL iterations as shown by the evolution of distribution of tested electrolytes and the cells' cycle life (color coded as shown in the legend) as iteration advances, which is herein representatively grouped by the type of additve for a clear visualization. **b-e**, Kernel density estimation of experimental and DAL results for the cycle life based on additives.

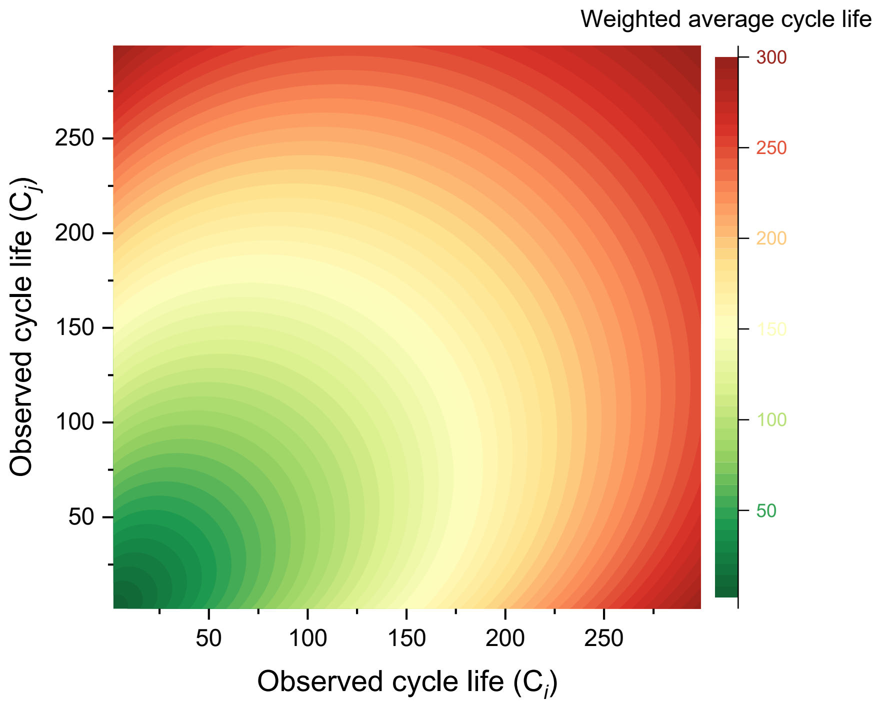

Supplementary Fig. 23 | The weighted average cycle life for the repetitive testing outcomes of cells.

Supplementary Note 1: Defining components within the parameter space

The selection of electrolyte components in this work deliberately avoids expert screening and relies on the lexicon method to identify relevant contents from the Web of Science database (**Supplementary Fig. 20a**). This led to the identification of lithium salts, solvents, and additives. We also selected the concentrations commonly used in the realm of LIBs, extending to those specific to LMBs — such as concentrations of 1m, 2m, 5m, as the representatives. It is worth noting that our search method is tailored to pinpoint and prioritize the electrolyte components most frequently used in current scientific literature. Yet, it does not incorporate expert insights into the correlation among the selected components.

To further diversify the components of electrolytes, DMC which is of a chain ester and ES with sulfur were selected (**Supplementary Fig. 20b**). For the same reason, we incorporated LiDFOB as a lithium salt (**Supplementary Fig. 20c**).

Supplementary Note 2: The robustness of DAL against noise

To address the frequently encountered high levels of noise interference in experiments, we conducted additional investigations by varying the noise levels, namely, $\epsilon \sim N(0, (5\% \times \max|f|)^2)$ (**Supplementary Fig. 4b**), $\epsilon \sim N(0, (10\% \times \max|f|)^2)$ (**Supplementary Fig. 4c**) and $\epsilon \sim N(0, (20\% \times \max|f|)^2)$ (**Supplementary Fig. 4d**), where f represents the standard deviation. These noise levels were chosen to explore the impact of noise on the performance of our method, and to evaluate its adaptability to different levels of noise. Large noise in the samples can initially misguide the surrogate model, leading to inappropriate sampling behavior, especially evident in the first iteration with a 20% noise level (**Supplementary Fig. 4d**). However, active learning can effectively mitigate this issue through its resampling process. By frequently sampling in the regions of high uncertainty, the surrogate model is fine-tuned to discern the system's noise level accurately, thus superior to a standard optimization. After three iterations, the DAL approach managed to achieve an optimal value of approximately 0.94 at a 5% noise level. Impressively, similar optimal values were observed even for noise levels of 10% and 20%. The results show that our proposed DAL approach possesses good adaptability to various levels of noise interference.

To further validate the effectiveness of DAL, we employed a more complex numerical function as a test case:

$$f = 20 + \sum_{i=1}^{2} [x_i^2 - 10\cos(4x_i^4)] \qquad (10)$$

where $\boldsymbol{x} = [x_1, x_2]$ is the input with 2 dimensions. The function has a global maximum value of 45.4 at (1.71, -1.71), but also contains many local maxima. The search space was defined as $[[0,2], [-2,0]]$.

We varied the noise level according to the normal distribution $\epsilon \sim N(0, (\{0, 5\%, 10\%, 20\%\} \times max\ |f|)^2)$ to evaluate the performance of the DAL approach in comparison with the GP-AL method (**Supplementary Figs. 4e** and **f**). When the noise level was set at 10%, the mean value of the objective function, after 50 iterations using the Matern-5/2 kernel function, was 37.2. This is in comparison to 41.7 in a noise-free scenario, showing a decrease in optimization performance of roughly 11%. In contrast, the performance of the DAL approach experienced a mere 0.4% decrease under identical conditions. This shows that the proposed DAL method has superior resilience to noise interference.

Supplementary Note 3: Exploring the impact of concentrations and additives on the electrolyte using DAL

The DAL method iteratively optimizes the initial electrolyte formulas and assesses its impact on cell cycle life. Although the method operates as a black box, insights into the impact of specific components can be obtained from the experimental data. For example, regarding the concentration of lithium salt in the electrolytes, we observe that the DAL exploration noticeably decreases in the region of 5m concentration as iterations progress. This implies it is generally hard to obtain long cell cycle life for high-concentration electrolytes (**Supplementary Fig. 21a**). In contrast, electrolyte formulas for the 1m and 2m regions consistently received high levels of exploration. Therefore, statistical analysis indicates that electrolytes with lithium salt concentrations of 1m and 2m are more likely to achieve a longer cell cycle life (**Supplementary Fig. 21a**).

After completing three iterations, we applied DAL to analyze all the experimental data. The results show that electrolyte formulas with 1m and 2m concentrations exhibit a broad distribution in the region associated with longer cell cycle life (**Supplementary Figs. 21b** and **c**). However, there are also a few predictions suggesting a more favorable distribution of cycle life in the 5m region. The predicting accuracy for electrolytes with concentrations of 5m may be lower than those with 1m and 2m due to the limited experimental data. Alternatively, it is plausible that an electrolyte at a 5m concentration could provide a relatively longer cycle life if used in a different electrolyte formula (**Supplementary Fig. 21d**). However, due to limited testing resources, the DAL method does not allocate extra resources to further explore the 5m concentration region.

Similarly, we also examined the impact of additives on the electrolyte's performance. In contrast to the impact of concentration, lithium salt, and solvent, the DAL method did not reveal significant disparities in evaluating the effects of additives (**Supplementary Fig. 22a**). This implies that the inclusion of specific type of additives appears to have less impact on the cycling stability of LMAs than other components do, in the studied parameter space. Nevertheless, enhanced performance was observed in the electrolyte formulas containing FEC (**Supplementary Figs. 22b** and **e**). Therefore, we conclude that while additives do contribute to some degree in optimizing electrolyte longevity, they are concluded here not the crucial factor in achieving stable LMAs.

<u>Supplementary Note 4: The weighting method for repetitive testing outcomes of electrolytes</u>

Experimental variability or noise can lead to inconsistencies in the experimental data. It typically leverages repetitive experimental measures to mitigate the impact of such errors. However, the cell cycle life of LMBs is subjected to multiple influencing factors during experimentation, often resulting in underreported test values. These factors such as environmental fluctuations consistently impede the acquisition of the best experimental value. Given these considerations, battery researchers often use a set of experimental values that indicate the best performance as the performance metric for the battery. Therefore, in our study we assigned higher weighting to outcomes that show long cell cycle life, as these are deemed to be more reflecting of the cell's inherent longevity. The weight for each repetitive test outcome is calculated as $c_{\mathrm{mean}} = \sum\left(c_i \times \frac{c_i}{\sum c_i}\right)$, where c_i represents the experimental cycle life as depicted in **Supplementary Fig. 23**. We believe this method yields a value that theoretically provides a more accurate approximation of the cell's true lifespan.

Supplementary Note 5: The criterion for the selection of the literature-inspired electrolyte

The criteria for literature-inspired electrolytes include: 1) They must contain the same components that compose the parameter space, ensuring a direct validation of the DAL optimization, as our DAL optimization focuses on the complex interactions between components. These literature-inspired electrolytes may or may not be in the initial parameter space. 2) They must have been previously verified and published and are known with good properties for the LMAs, so that we can best eliminate bias introduced by our own experimental results.

Supplementary Note 6: The collection and consideration of prior knowledge

Quantifying the impact of interactions among electrolyte components on the cells' cycle life is crucial, given the high discreteness as shown in our study. However, due to the substantial variations in the exact electrolyte formulas and the cell testing conditions among reported studies in the literature, it is nearly impossible to statistically encode the published data. To obtain and assess the information of related electrolytes in the literature, and to generate the prior knowledge, we adopted the following approach: (1) Using electrolyte components as the search keywords, we manually examined all related publications in the Web of Science database, and evaluated the components' impact on the LMB performance based on experimental data; (2) With that, we consider the cycle life of the $Li^0|Li^0$ symmetric cells, the Coulombic efficiency of Li-Cu cell, and cycle life of full LMB cell as the prior knowledge; (3) We further incorporate all data in the supporting information/materials from the publications.

As the electrolyte formulas in the literature is not always the same with the parameter space defined in our study in terms of the exact chemicals and their ratios, it is hard to rigorously summarize and align the data to our study. To solve the problem, we processed the prior knowledge with the following screening criteria: (1) The electrolyte components need to match with those in the defined parameter space in our study, or to exhibit similar chemical structures; (2) The proportions of solvents are not strictly constrained to be the same with those in our study; (3) The notations of volumetric and weight molar concentrations of lithium salts are both included and considered to be the same values; (4) if the reported concentrations are different from those in our study, the "performance" at the concentration of our study (1m, 2m , 5m) will be inferred based on assumption of electrolyte concentration impact can be continuous to some extent (for example, one reported electrolyte with a 4m or 6m concentration may very well show comparable performance with one electrolyte of 5m, and thus its impact on the cell cycle life is considered as a prior knowledge for 5m), (5) If multiple studies present consistent or similar outcomes, only one of them is included as prior knowledge; (6) In case of multiple reports showing inconsistent results, the outcome is excluded as prior knowledge due to uncertainty. The full list of generated prior knowledge (and new knowledge) is shown in **Data S2.**

Supplementary Note 7: The performance of DAL in the numerical simulations

Figure 2d depicts the performance of the proposed DAL approach on the function $f = (1-|x|)\sin(20(1-|x|)^6)$ with noise $\epsilon \sim N(0, (5\% \times \max|f|)^2)$. In this numerical example, we initialized the initial round by randomly sampling 20 data points. Subsequent first and second iterations were guided by parallel Thompson sampling with a budget for 10 evaluations. The true objective function is represented by the orange curve, which exhibits rapid oscillations proximate to the origin and levels out further away, posing a challenge for accurate modeling. Relying on a Gaussian process (GP) with stationary covariance without substantial samples, there has to sacrifice accuracy on the segments of the function that exhibit more subtle variations. Contrasting this with our DAL approach (**Fig. 2d**), the GP-AL method maintained the same optimization settings but trapped into a local optimal solution in the iteration, resulting in low sample efficiency (**Supplementary Fig. 3a**). After three iterations, the mean square error obtained by the DAL approach was 42.2% lower than that by GP-AL, and the optimal solution obtained by DAL outperforms GP-AL by 9.9%.

We used the same function setup as described in **Supplementary Note 2** to evaluate the performance of the DAL approach in numerical scenarios (**Supplementary Figs. 3b** and **c**). We compared the optimization results of DAL, which adopted varied network structures, against those of GP-AL that utilized diverse kernel functions (**Supplementary Figs. 3b** and **c**). For these comparisons, we introduced a 5% noise level to the experiment. For GP-AL, Matérn covariance functions are commonly adopted as kernel functions. These functions are controlled by a smoothing parameter $\nu > 0$ with specific expression, which is described as

$$\kappa_{M-\frac{1}{2}} = \sigma_c^2 \exp\left(-\frac{r}{l}\right) \tag{11}$$

$$\kappa_{M-\frac{3}{2}} = \sigma_c^2 \left(1 + \frac{\sqrt{3}r}{l}\right) \exp\left(-\frac{\sqrt{3}r}{l}\right) \tag{12}$$

$$\kappa_{M-\frac{5}{2}} = \sigma_c^2 \left(1 + \frac{\sqrt{5}r}{l} + \frac{5r^2}{3l^2}\right) \exp\left(-\frac{\sqrt{5}r}{l}\right) \tag{13}$$

$$\kappa_{M-\infty} = \sigma_c^2 \exp\left(-\frac{r^2}{2l^2}\right) = \kappa_{RBF} \tag{14}$$

where $r = ||x - x'||$, and l are scale parameters. When the smoothing parameter ν is equal to 1/2, the Matérn kernel becomes an exponential kernel, leading to large fluctuations in the associated parts of the process. When ν approaches infinity, the Matérn kernel evolves into a squared exponential kernel, also referred to as the Radial Basis Function (RBF) kernel. The associated process then shows infinite mean square differentiability, resulting in an extremely smooth regression model.

For DAL, we implemented fully connected neural networks with structures 10-2, 20-2, and 10-10-2, corresponding to networks with 10, 20, and 10 neurons per layer, respectively. As shown in **Supplementary Fig. 3b**, making the networks deeper or wider did not significantly improve optimization performance after 50 iterations. The mean optimal values for 10-2, 20-2, and 10-10-2 were 42.88, 42.69, and 42.94, respectively, demonstrating the robustness of DAL across network configurations. In contrast, GP-AL exhibited slower convergence. After 50 optimization iterations, its mean optimal values for Matérn-1/2, Matérn-3/2, Matérn-5/2, and RBF kernels were 40.38, 40.25, 39.85, and 33.89, respectively, all significantly lower than those achieved by DAL. Additionally, GP-AL suffered from high variance across multiple runs, highlighting sensitivity to initial sample selection (**Supplementary Fig. 3c**). For

example, while DAL with the 10-2 network structure had a standard deviation of only 1.4, GP-AL with the Matérn-5/2 kernel exhibited a much higher deviation of 7.6.

These results demonstrate that DAL consistently outperforms GP-AL in terms of accuracy, stability, and sample efficiency, making it a more reliable approach for optimization under limited data conditions.

Supplementary Table 1. The experimental cycle life for the electrolyte used in the batch of random selection.

Electrolyte formulas	cycle life*
1m $LiPF_6$ EC/DMC+additive-free	64.25714
1m $LiPF_6$ EC/DMC+5wt.% FEC	42.26027
1m $LiPF_6$ EC/DME+5wt.% VC	89.10112
2m $LiPF_6$ DMC/DME+5wt.% VC	13
1m $LiPF_6$ DMC/ES+additive-free	6.846154
1m $LiPF_6$ DMC/PC+5wt.% VC	16.63636
5m LiTFSI EC/DMC+additive-free	19.23077
1m LiTFSI EC/PC+5wt.% $LiNO_3$	15.26667
5m LiTFSI DMC/DME+5wt.% $LiNO_3$	27.92593
2m LiTFSI DMC/DOL+5wt.% FEC	24.85714
5m LiTFSI DMC/ES+5wt.% VC	6.538462
5m LiTFSI DME/DOL+5wt.% FEC	4.25
2m LiFSI EC/DMC+5wt.% $LiNO_3$	9
1m LiFSI EC/DME+5wt.% FEC	197.1101
1m LiFSI EC/ES+additive-free	66.02941
1m LiFSI DMC/DME+5wt.% FEC	20.5122
1m LiFSI DMC/DOL+5wt.% $LiNO_3$	30.13333
2m LiFSI DMC/ES+5wt.% FEC	10.1
5m LiFSI DMC/PC+5wt.% FEC	2.6
5m LiFSI DMC/PC+5wt.% $LiNO_3$	6
1m LiFSI DME/DOL+additive-free	142.4675
1m LiFSI DME/PC+5wt.% VC	27.58182
5m LiFSI DOL/PC+additive-free	15.06667
2m LiFSI ES/PC+5wt.% VC	8.333333
1m LiDFOB EC/DME+5wt.% $LiNO_3$	48.33333
2m LiDFOB DMC/DME+additive-free	8.666667
2m LiDFOB DME/ES+additive-free	272.9853
2m LiDFOB DME/ES+5wt.% FEC	48.60656
1m LiDFOB DOL/PC+additive-free	32.16129
2m LiDFOB ES/PC+additive-free	10.78947
2m LiDFOB ES/PC+5wt.% $LiNO_3$	12.33333

*The cycle life in the batch of random selection is calculated according to the method discussed in **Supplementary Note 4**.

Supplementary Table 2: The experimental cycle life for the electrolyte used in the batch of 1st iteration.

Electrolyte formulas	cycle life
2m LiDFOB DME/ES+additive-free	65
1m LiFSI EC/DME+5wt.% FEC	150
2m LiDFOB DME/DOL+additive-free	67
1m LiDFOB DME/ES+additive-free	10
5m LiDFOB DME/ES+additive-free	12
2m LiFSI DME/ES+additive-free	13
2m LiDFOB DME/PC+additive-free	49
1m LiDFOB DME/DOL+additive-free	20
2m LiDFOB DME/ES+5wt.% VC	45
2m LiFSI DME/DOL+additive-free	254
1m LiFSI EC/DME+5wt.% VC	167
1m LiFSI DME/DOL+additive-free	6
5m LiDFOB DME/DOL+additive-free	2
1m LiFSI DME/ES+additive-free	106
1m LiFSI EC/DOL+5wt.% FEC	120
1m LiTFSI EC/DME+5wt.% FEC	168
5m LiFSI DME/ES+additive-free	13
5m LiDFOB DME/PC+additive-free	28
2m LiDFOB DME/DOL+5wt.% VC	40
1m LiTFSI EC/DME+5wt.% VC	134
1m LiFSI EC/DOL+5wt.% VC	97
1m LiDFOB DME/PC+additive-free	44
5m LiDFOB DME/ES+5wt.% VC	10
1m LiDFOB DME/ES+5wt.% VC	12
1m LiDFOB EC/DME+5wt.% FEC	10
2m LiFSI DME/PC+additive-free	47
2m LiFSI EC/DME+5wt.% FEC	6
1m LiFSI EC/DMC+5wt.% FEC	37
1m LiTFSI EC/DOL+5wt.% FEC	125
1m LiDFOB DME/DOL+5wt.% VC	27
1m LiDFOB EC/DME+5wt.% VC	14
5m LiFSI DME/DOL+additive-free	43

Supplementary Table 3. The experimental cycle life for the electrolyte used in the batch of 2nd iteration.

Electrolyte formulas	cycle life
2m LiFSI DME/DOL+additive-free	150
2m LiDFOB DME/ES+additive-free	30
1m LiFSI EC/DME+5wt.% FEC	123
1m LiTFSI EC/DME+5wt.% FEC	188
1m LiFSI EC/DME+5wt.% VC	103
1m LiFSI DME/PC+5wt.% $LiNO_3$	5
1m LiFSI DME/DOL+additive-free	176
1m LiTFSI DME/DOL+5wt.% VC	145
1m LiTFSI EC/DME+5wt.% VC	86
1m LiTFSI DME/ES+5wt.% VC	184
1m LiTFSI EC/DOL+5wt.% FEC	125
2m LiFSI DME/PC+5wt.% $LiNO_3$	121
1m LiTFSI ES/PC+5wt.% $LiNO_3$	32
1m LiTFSI DME/PC+5wt.% FEC	76
1m LiFSI EC/DOL+5wt.% FEC	120
1m LiPF6 ES/PC+5wt.% FEC	15
2m LiFSI DMC/PC+5wt.% VC	45
2m LiPF6 ES/PC+5wt.% VC	18
1m LiTFSI DME/ES+5wt.% FEC	181
1m LiTFSI DME/PC+5wt.% VC	97
1m LiFSI DME/DOL+5wt.% FEC	158
2m LiTFSI DME/ES+additive-free	7
2m LiTFSI DME/PC+additive-free	63
1m LiTFSI DOL/ES+5wt.% VC	48
1m LiTFSI ES/PC+5wt.% VC	6
1m LiTFSI DOL/PC+5wt.% VC	67
1m LiTFSI DOL/PC+5wt.% $LiNO_3$	77
1m LiFSI DME/ES+additive-free	144
1m LiFSI DOL/ES+5wt.% $LiNO_3$	11
1m LiDFOB DME/ES+5wt.% $LiNO_3$	36
2m LiTFSI DOL/ES+additive-free	72
1m LiFSI EC/DOL+5wt.% VC	95

Supplementary Table 4. The experimental cycle life for the electrolyte used in the batch of 3rd iteration.

Electrolyte formulas	cycle life
1m LiTFSI EC/DME+5wt.% FEC	152
2m LiTFSI DME/DOL+additive-free	213
1m LiTFSI DME/DOL+5wt.% VC	152
1m LiTFSI DME/ES+5wt.% VC	240
2m LiTFSI DME/ES+5wt.% VC	240
1m LiTFSI DME/ES+5wt.% FEC	173
1m LiTFSI DME/PC+5wt.% VC	72
1m LiTFSI DME/PC+5wt.% FEC	42
2m LiTFSI DME/PC+5wt.% FEC	87
2m LiTFSI DOL/ES+additive-free	45
2m LiTFSI DOL/PC+5wt.% VC	56
5m LiTFSI DOL/PC+5wt.% $LiNO_3$	48
1m LiFSI EC/DME+5wt.% VC	138
1m LiFSI DME/DOL+additive-free	262
2m LiFSI DME/DOL+additive-free	289
1m LiFSI DME/DOL+5wt.% VC	80
1m LiFSI DME/DOL+5wt.% FEC	98
1m LiFSI DME/DOL+5wt.% $LiNO_3$	314
1m LiFSI DME/ES+additive-free	176
1m LiFSI DME/ES+5wt.% VC	195
1m LiFSI DME/ES+5wt.% FEC	157

1m LiFSI DME/PC+additive-free	97
2m LiFSI DME/PC+5wt.% VC	139
1m LiFSI DME/PC+5wt.% FEC	101
2m LiFSI DOL/ES+5wt.% FEC	29
2m LiFSI DOL/PC+5wt.% $LiNO_3$	125
1m LiDFOB EC/ES+5wt.% VC	24
1m LiDFOB DME/DOL+5wt.% $LiNO_3$	33
2m LiDFOB DME/ES+5wt.% VC	22
1m LiDFOB DME/ES+5wt.% $LiNO_3$	31
2m LiDFOB DME/PC+5wt.% FEC	59
2m LiDFOB DOL/ES+5wt.% $LiNO_3$	29

Supplementary Table 5. The experimental capacity retention of Li^0|LMO full cell for the electrolyte firstly recommended by DAL after knowledge transfer to new parameter space with new molecules.

Electrolyte formulas	Capacity retention after 150 cycles (100%)
0.5m LiTFSI 0.5m LiDFOB DME/EC/DEP+VC/FEC/$LiNO_3$	97.55
0.5m LiTFSI 0.5m LiFSI DME/EC/DEP+VC/$LiNO_3$	91.3
0.5m LiTFSI 0.5m LiFSI DME/ES/DEP+VC/$LiNO_3$/TO	89.99
0.5m LiTFSI 0.5m LiDFOB PC/ES/DEP+FEC/$LiNO_3$	86.27
0.5m LiPF6 0.5m LiFSI DME/PC/EC+FEC/$LiNO_3$/TO	85.52
0.5m LiTFSI 0.5m LiFSI DME/DOL/PC+VC/FEC/$LiNO_3$/TO	68.37
0.75m LiTFSI 0.75m LiFSI DME/DMC/EC+FEC/$LiNO_3$/TO	68.12
1m LiTFSI 1m LiFSI DME/PC/EC+VC/$LiNO_3$/TO	67.88
0.5m LiTFSI 0.5m LiFSI DME/EC/DEP+FEC/LiNO3/TO	67.73
0.5m LiTFSI 0.5m $LiPF_6$ DME/EC/DEP+VC/TO	42.91
1.25m LiTFSI 1.25m LiFSI DOL/EC/DEP+FEC/TO	3.51
0.5m LiTFSI 0.5m LiPF6 DME/PC/DEP+VC/FEC/TO	1.95
0.5m LiTFSI 0.5m $LiPF_6$ PC/ES/DEP+VC/FEC	1.74
2.5m LiTFSI 2.5m LiFSI DMC/PC/DEP+VC/TO	1.61
0.75m LiTFSI 0.75m LiFSI DME/DOL/PC+VC/FEC/TO	1.58
0.5m LiTFSI 0.5m LiFSI DME/DOL/ES+VC/$LiNO_3$/TO	1.01
0.5m LiTFSI 0.5m LiFSI ES/PC/DEP+VC/TO	0.98
1m LiTFSI 1m LiFSI PC/ES/DEP+VC/FEC/TO	0.86
1m LiTFSI 1m LiFSI DOL/PC/DEP+FEC/TO	0.84
0.5m LiTFSI 0.5m LiFSI PC/ES/DEP+VC/FEC/TO	0.57
1m LiTFSI 1m LiFSI DME/PC/EC+VC/TO	0.51
0.5m LiTFSI 0.5m LiFSI DME/DOL/DEP+	0.43
0.5m LiTFSI 0.5m LiFSI DME/PC/DEP+FEC/TO	0.38
0.5m LiTFSI 0.5m LiFSI PC/ES/DEP+VC/FEC/TO	0.31
0.5m LiTFSI 0.5m LiFSI DMC/PC/DEP+VC/FEC	0.26
0.5m LiTFSI 0.5m LiFSI DME/PC/ES+FEC/$LiNO_3$/TO	0.21
0.75m LiPF6 0.75m LiFSI DME/EC/DEP+VC/FEC/TO	0.03
1m LiTFSI 1m LiFSI DMC/DOL/EC+VC/FEC/TO	0.03
2.5m LiTFSI 2.5m LiFSI DOL/EC/DEP+FEC/$LiNO_3$	0
0.5m LiTFSI 0.5m LiFSI DME/EC/DEP+VC/FEC	0
1m LiTFSI 1m LiFSI DME/EC/DEP+additive-free	0
0.75m LiTFSI 0.75m LiFSI DMC/EC/DEP+VC/FEC	0

Supplementary Table 6. The experimental capacity retention of Li^0|LMO full cell for the electrolyte secondly recommended by DAL after 1st iteration in new parameter space with new molecules.

Electrolyte formulas	Capacity retention after 150 cycles (100%)
1m LiTFSI 1m LiFSI PC/EC/DEP+FEC/$LiNO_3$	99.59
0.5m LiTFSI 0.5m LiFSI DME/PC/EC+FEC/$LiNO_3$/TO	98.72
0.5m LiTFSI 0.5m LiFSI DME/PC/EC+FEC/$LiNO_3$/TO	97.7
0.5m LiTFSI 0.5m LiFSI DME/PC/DEP+FEC/$LiNO_3$/TO	97.02
0.75m LiTFSI 0.75m LiFSI DME/PC/DEP+VC/FEC/$LiNO_3$/TO	95.23
0.75m LiTFSI 0.75m LiFSI DME/PC/DEP+VC/$LiNO_3$/TO	95.22
0.5m LiTFSI 0.5m LiFSI DME/PC/DEP+VC/FEC/TO	94.54
0.5m LiTFSI 0.5m LiFSI DME/PC/DEP+VC/$LiNO_3$/TO	92.41
0.75m LiTFSI 0.75m LiDFOB DME/ES/EC+VC/$LiNO_3$/TO	89.1
0.5m LiTFSI 0.5m LiFSI DME/ES/EC+VC/FEC/TO	88.53
0.5m LiPF6 0.5m LiFSI DME/ES/EC+FEC/$LiNO_3$/TO	84.1
0.75m LiTFSI 0.75m LiDFOB DME/ES/EC+VC/FEC/TO	83.58
1m LiTFSI 1m LiDFOB DME/ES/EC+VC/FEC/$LiNO_3$/TO	82.85
0.5m LiTFSI 0.5m LiDFOB DME/ES/EC+VC/$LiNO_3$/TO	81.45
0.75m LiTFSI 0.75m LiDFOB DME/ES/EC+FEC/$LiNO_3$/TO	80.44

1m LiTFSI 1m LiDFOB DME/ES/EC+VC/$LiNO_3$/TO	78.47
0.5m LiTFSI 0.5m LiFSI DME/ES/DEP+VC/$LiNO_3$/TO	78.4
0.75m LiTFSI 0.75m LiFSI DME/ES/DEP+$LiNO_3$/TO	78.39
0.5m LiTFSI 0.5m LiDFOB DME/ES/DEP+VC/$LiNO_3$/TO	78.2
0.75m LiTFSI 0.75m LiFSI DME/ES/DEP+FEC/$LiNO_3$/TO	61.16
0.75m LiTFSI 0.75m LiDFOB DME/ES/DEP+$LiNO_3$/TO	22.33
0.75m LiTFSI 0.75m LiDFOB DME/ES/DEP+$LiNO_3$/TO	21.2
1m LiTFSI 1m LiDFOB DME/ES/DEP+FEC/$LiNO_3$/TO	14.61
0.5m LiTFSI 0.5m LiDFOB DME/EC/DEP+VC/FEC/$LiNO_3$	8
0.75m LiTFSI 0.75m LiFSI DME/EC/DEP+VC/$LiNO_3$	2.69
0.75m LiTFSI 0.75m LiFSI DME/EC/DEP+VC/FEC/$LiNO_3$	0.88
1m LiTFSI 1m LiFSI DME/EC/DEP+VC/FEC/$LiNO_3$	0.71
1m LiTFSI 1m LiFSI DME/EC/DEP+FEC/$LiNO_3$/TO	0.64
1m LiTFSI 1m LiFSI DMC/DOL/PC+VC/FEC/TO	0.46
1m LiTFSI 1m LiFSI DMC/DOL/PC+VC/FEC/TO	0.45
1m LiTFSI 1m LiFSI DMC/DOL/PC+VC/FEC/TO	0.13
1m LiTFSI 1m LiFSI DMC/DOL/ES+VC/$LiNO_3$/TO	0.07
