# Supplementary material for: Deep active learning and knowledge transfer for rapid discovery of lithium metal battery electrolytes
Source: Nat Commun. 2026 Mar 27;17:5146. doi: 10.1038/s41467-026-70973-4 (PMC13250146; doi:10.1038/s41467-026-70973-4)
Supplement: Supplementary file 3 — Description of Additional Supplementary Files [file 41467_2026_70973_MOESM3_ESM.pdf]

Description of Additional Supplementary Files

Supplementary Data 1: The detailed electrolyte formulas in initial constructed parameter space.

Supplementary Data 2: The prior knowledge of interaction between components reported in the literature.
