# Supplementary material for: Deep active learning and knowledge transfer for rapid discovery of lithium metal battery electrolytes
Source: Nat Commun. 2026 Mar 27;17:5146. doi: 10.1038/s41467-026-70973-4 (PMC13250146; doi:10.1038/s41467-026-70973-4)
Supplement: Supplementary file 2 — Peer Review File [file 41467_2026_70973_MOESM2_ESM.pdf]

nature portfolio

Peer Review File

Deep active learning and knowledge transfer for rapid discovery of lithium metal battery electrolytes

Corresponding Author: Professor Benben Jiang

This file contains all reviewer reports in order by version, followed by all author rebuttals in order by version.

Parts of this Peer Review File have been redacted as indicated to remove third-party material.

Version 0:

Reviewer comments:

Reviewer #1

(Remarks to the Author)
The manuscript by Hong et al., presents a sophisticated deep active learning framework to accelerate materials discovery. By integrating deep kernel learning with TSC, the authors have developed a two-stage AI framework that addresses a key challenge in the field by effectively exploring the discontinuous property landscapes of electrolytes. Critically, this framework can also dynamically incorporate new, advanced molecules into the optimization process without requiring a full restart. This AI framework is shown to substantially accelerate the identification of high-performance candidates within complex, multicomponent electrolyte systems.

This study is both timely and significant. It demonstrates a meaningful integration of experimental investigation with advanced machine learning methodologies, highlighting the importance of aligning algorithmic development with realistic experimental scenarios. The authors provide convincing experimental evidence that their framework mitigates key challenges arising from high chemical discontinuities and large experimental noise, thereby enhancing the reliability and efficiency of the electrolyte discovery process.

Overall, this work represents a valuable contribution to the field of energy storage and will be of broad interest to researchers focused on electrolyte design and data-driven materials discovery. I endorse its publication in Nature Communications and provide the following comments to further strengthen the manuscript.

1. During the knowledge transfer stage, ADI values were calculated to guide further electrolyte design via DAL. Could the authors elaborate on whether these values could also serve as practical indicators for electrolyte chemists, thereby enabling more rational and efficient electrolyte formulation?
2. The electrolyte for lithium metal batteries needs to balance the stability of both the lithium metal anode and the cathode. The authors first evaluated lifespan in Li||Li symmetric cells and then extended the exploration to full-cell systems. Would direct application to full-cell configurations be feasible and potentially more efficient?
3. The comparison between TSC-derived knowledge and reported results shows an 85% overlap, but also a 15% discrepancy. Will this discrepancy may influence the effectiveness of subsequent optimization strategies?
4. While BO has been applied and discussed, there exist other promising optimization methods (e.g., PHOENICS or other global optimization algorithms). A brief discussion of alternative strategies may broaden the applicability and impact of the proposed framework.
5. In repeated experimental results, such as those presented in Fig. 3D and Fig. 5C, the standard deviation of the measured lifetimes appears to be relatively large. The author should provide the reasons for such large deviation.
6. In exploring a larger parameter space, the authors employed a broader range of components, including two types of salts, three types of solvents, and multiple additives, resulting in 5,400 different electrolyte combinations. What were the practical considerations behind this configuration?

(Remarks on code availability)
The code runs successfully, allowing the main results to be reproduced.

Reviewer #2

(Remarks to the Author)
The study by Hong et al. presents a deep active learning (DAL) approach for optimization of electrolyte formulations for lithium metal battery (LMB) full cells using deep kernel learning (DKL). The approach was successfully used by the authors to find efficient electrolytes compared to initial electrolytes tested. The authors also developed a target statistic encoding (TSC) framework to quantify learned knowledge and then leverage it to find newer electrolytes with few-shot learning.
The present study provides certain advancements in the data-driven battery materials discovery field and carries out >100 real-world battery testing. However, there are significant limitations and loopholes in the developed approach that should be addressed to make it truly generalizable. Here are detailed comments:
1. The manuscript revolves around the central idea that their approach can be utilized to perform optimization of electrolyte properties in the discrete electrolyte chemical space as they mentioned it multiple times in the introduction and elsewhere: "… these approaches have been confined to scenarios featuring a numerically continuous space." However, some of the recent works have already been developed utilizing AL framework to carry out optimization of electrolytes in the discrete space, e.g., Noh et al., Nat Commun 15, 2757 (2024); Zhang et al., Mater. Today Energy 51, 101909 (2025). It should be clearly stated along with proper citations of these references. I have further reservations on their "optimization in discrete space" claim, which I will point out below.
2. The authors also mention "large experimental variability" in reporting battery testing metrics such as cycle life, which is certainly true and is also apparent from Figures 3 and 5. So, I commend the authors for reporting such true and large variations in the manuscript, which is seldom reported in the literature. But my question is how to predict such large measurement noise, i.e., aleatoric uncertainties? A Bayesian technique such as DKL can be used to predict such aleatoric uncertainties, so the authors should check if their DKL model can indeed reproduce the noise in their measurements. It will be highly beneficial to the community.
2. The authors did not open source their codes, which is one of the requirements of the journal. While they did provide access to their partial codebase, I would also need access to data, model checkpoints and remaining codes in order to ensure it is fully reproducible. The provided codebase is also not well-documented. So, I recommend that the authors upload all their codes, datasets, and model checkpoints on GitHub and/or Zenodo with detailed documentation on how to use it including the python libraries involved.
3. Why choose Thompson sampling as acquisition function over standard and more common acquisition functions such as the expected improvement? There is no justification provided in the draft.
4. The optimization is carried over only 13 molecular components (6 solvents, 4 salts, 3 additives) and later two more molecular components were added in the search space. While we know that molecular chemical space is enormous, estimated to be on the order of 1060. So, is their approach truly carrying out optimization over discrete electrolyte space then? The search space is also very small – 720, 5400 and 5726. Do we really need AL for such small spaces? AL is usually carried over large search spaces on the order of thousands, if not millions, of input samples. A frequentist ML model can well be utilized to find optimal candidates in such small search spaces.
5. In Figure 2e, I believe the comparison between GP-AL and DAL is made using the RBF covariance kernel in GPs. While it is true that RBF is suited for smooth and continuous functions, there are also other standard covariance kernels that can be used when there is roughness in the functions, e.g., Matern, rational quadratic, pairwise, etc. Multiple covariance kernels can also be combined together to achieve the desired effect. So, their choice of using DKL over GP in the present study is not well justified. They should compare GPs with aforementioned covariance kernels to truly establish the superiority of DKL over GPs in such scenarios.
6. Along the same lines, the authors mention that "…the black-box functions with rapid oscillations, which are characteristic of the objective function in the electrolyte optimization problem…" But it has not been explicitly shown that the objective function for electrolyte optimization problem is indeed "rapidly oscillating".
7. The authors also make claims about DAL framework being able to balance between exploration and exploitation. This can be explicitly shown on a reduced latent space representation of electrolytes from the trained DKL model by showing suggestions from different batches. If the model is indeed exploiting, then the suggestions from later batches should converge to a particular region of the chemical space. The authors can also show distribution of uncertainties for suggestions from different batches and if the model is progressing towards exploitation, then the distribution of uncertainties should shift towards lower values.
8. How are the molecular components represented? I do not see any discussion in the Methods section. Do they represent molecules using Morgan fingerprints or RDKit features or graph neural networks (GNN) or some sort of one-hot encoding? If one-hot encoding is used to represent molecular components in the electrolytes, then their model cannot really be inferred on molecular components unseen by the DKL model. If that's the case, then the claim of "optimization over discrete electrolyte space" again does not really hold true.
9. I also have reservations about their developed TSC approach. The average degree of interaction (ADI) values from this TSC approach can only be obtained post training of the DKL model and is not learned during the training itself. This limits the utility of this approach. Also, continuing from previous point, one needs to know the ADI values of unseen molecules a priori in order to make predictions: "For TSC coding the initial ADIs of DEP-or TO-related interactions were assigned as the prior p, which corresponds to the mean values of existing interactions." This will severely limit the applicability of their approach on finding electrolytes constituting unexplored solvent, salts, or additives, as one would need to first perform real-world experiments using these unexplored molecular components.
10. Other non-technical comments:
a) The introduction section is a bit too long. Even though there is no word limit in the Nature Communications journal, I think it contains redundant information and so can be easily reduced to two-thirds.
b) Some of the sentences are also too long and are hard to follow, e.g.:
"To complicate it further, unlike the optimization of nonchemical (particularly numerical) subjects of interest – the examples of which include battery fast-charging protocols, and physical properties of electrolyte (solubility and concentration, ionic conductivities and viscosity) that vary over a continuous space of numbers – the materials space for electrolyte design is highly discrete, because the component-performance relationship is highly sensitive to many equilibria and inequilibria

processes existing in the cell environment, such as the interphasial chemistry and related properties, where the change of a single chemical or its concentration may trigger an exponential increase in potential component interactions, resulting in the combinatorial explosion of search space."
c) Some of the words used in the draft can be replaced with more suitable ones, e.g., "recommendation" with "suggestion" since the word recommendation gives the idea that a recommendation system is being utilized to get new suggestions in each batch, which is not the same as an AL framework.
b) Add a separate "Discussion" section in the revised draft (which is allowed as per journal's guidelines) clearly highlighting limitations of their approach as pointed out above.

(Remarks on code availability)
Comments on code availability have been discussed in the overall comments to the authors.

Reviewer #3

(Remarks to the Author)
The author describes a deep active learning approach to discover new electrolytes for lithium metal batteries. Overall, the work is well structured and clearly presented. I believe that the novelty lies in the combination of deep learning and active learning. However, the author primarily focuses on convincing readers of the importance of this technique for improving the efficiency of electrolyte screening for lithium metal batteries. The main conclusions of the work appear somewhat scattered. I suggest that the author highlight the key novelty of the study more explicitly. I have some concerns for the author to consider, listed below:
1.The work illustrated in the paper is too specific, and I can imagine that the author spent substantial time completing the experiments. How, then, can the author convince us that this method truly reduces time costs?
2.The performance of lithium metal batteries is important; however, it is still not sufficient to represent or transfer the overall performance to other devices. Thus, the experimental design is critical. LMO is not a typical cathode for lithium metal batteries—why not use NCM cathodes for validation?
3.The authors need to emphasize the importance and necessity of DKL and Thompson sampling by comparing them with the standard BO process.
4.Overall, I think this work is highly interdisciplinary. Researchers from the battery field and the machine learning field could easily become confused. Therefore, integrating the two effectively will be a major challenge for the authors to address in order to improve the work.

(Remarks on code availability)
The explanation of the code is not very well incorporated. I think the code needs to be commented better for broader audience.

Version 1:

Reviewer comments:

Reviewer #1

(Remarks to the Author)
The manuscript has been revised pretty well according to the 1st round review. All my concerns have been well addressed and I believe this work can cause wide interests and broad impact.

(Remarks on code availability)

Reviewer #2

(Remarks to the Author)
The authors have provided a detailed response to my comments in the first round of revision and many of my comments have been satisfactorily addressed, including the release of codes and datasets. However, some critical comments remain unaddressed. See below:
1. Comment #2 in the first round on predicting aleatoric uncertainties has not been taken care of but can be addressed. I recommend the authors to follow this reference for guidance: Zhong et al., Nat. Commun. 16, 4522 (2025). Predicting aleatoric uncertainties is critical for quantifying reproducibility in the experimental measurements.
2. Comparison of t-SNE plots of all electrolytes (suggested ones should be clearly highlighted) from different batches in point #7 in the first round are important. The authors can refer to this reference for guidance: Khalak et al., J. Chem. Theory Comput. 18, 6259-6270 (2022) (Figure 5).
3. The authors' reasons for not using any chemical representation in the present framework is not justified (point #8 in the first round). If data-scarcity was the issue then one can use a pretrained model to generate molecular features for solvents, salts, and additives and feed to the surrogate model. The current approach may not lead to any new molecular design of electrolytes in a zero-shot manner, which stands in contrast to their claims of "optimization over discrete electrolyte space".
4. When I asked to include a "Discussion" section, the goal was for the authors to clearly highlight the limitations. Currently, it is very brief and looks very generic and none of the main issues of their approach, which they themselves accept in responses to points #2, #8, and #9 from the first round, have been explicitly highlighted in the manuscript. It is important to highlight all the limitations of their approach such that the reader is better informed and future.

(Remarks on code availability)

Reviewer #3

(Remarks to the Author)
I appreciate the response of the authors to my comments. I think the questions are overall very well presented. However, the answer to my Comment #4 is vague. I cannot find the details about the improvements completed by the author. The improvements to enhanced the significant of this work to the audience is highly essential. I hope this point can be addressed further.

(Remarks on code availability)

Version 2:

Reviewer comments:

Reviewer #2

(Remarks to the Author)
The comments have been addressed.

(Remarks on code availability)

Open Access This Peer Review File is licensed under a Creative Commons Attribution 4.0 International License, which permits use, sharing, adaptation, distribution and reproduction in any medium or format, as long as you give appropriate credit to the original author(s) and the source, provide a link to the Creative Commons license, and indicate if changes were made.
In cases where reviewers are anonymous, credit should be given to 'Anonymous Referee' and the source.
The images or other third party material in this Peer Review File are included in the article's Creative Commons license, unless indicated otherwise in a credit line to the material. If material is not included in the article's Creative Commons license and your intended use is not permitted by statutory regulation or exceeds the permitted use, you will need to obtain permission directly from the copyright holder.
To view a copy of this license, visit https://creativecommons.org/licenses/by/4.0/

Response Letter to Reviewers' Comments on Manuscript

NCOMMS-25-60533

We thank the reviewers for their valuable comments. In response, we further elaborated on the details of the algorithm, provided explanations regarding data noise, reliability, and the design of the two-stage optimization framework, as well as made textual technical modifications to enhance the logicality and readability, diligently addressed each comment and incorporated the suggestions into the revised manuscript (major changes highlighted in red for clarity), as explained below in detail. Answers to the reviewers' comments are presented in blue type, while a portion of the updates to the manuscript and Supplemental Information are copied here and marked in red.

Reviewer #1 (Remarks to the Author):

The manuscript by Hong et al., presents a sophisticated deep active learning framework to accelerate materials discovery. By integrating deep kernel learning with TSC, the authors have developed a two-stage AI framework that addresses a key challenge in the field by effectively exploring the discontinuous property landscapes of electrolytes. Critically, this framework can also dynamically incorporate new, advanced molecules into the optimization process without requiring a full restart. This AI framework is shown to substantially accelerate the identification of high-performance candidates within complex, multicomponent electrolyte systems.

This study is both timely and significant. It demonstrates a meaningful integration of experimental investigation with advanced machine learning methodologies, highlighting the importance of aligning algorithmic development with realistic experimental scenarios. The authors provide convincing experimental evidence that their framework mitigates key challenges arising from high chemical discontinuities and large experimental noise, thereby enhancing the reliability and efficiency of the electrolyte discovery process.

Overall, this work represents a valuable contribution to the field of energy storage and will be of broad interest to researchers focused on electrolyte design and data-driven materials discovery. I endorse its publication in Nature Communications and provide the following comments to further strengthen the manuscript.

Our Response: We sincerely thank the reviewer for the positive evaluation of our work on integrating deep active learning with TSC for accelerated electrolyte discovery. As highlighted, our framework was specifically designed to address key practical challenges in electrolyte development, including the discontinuous property landscape, substantial experimental noise, and the dynamic incorporation of new molecules. By adopting a two-stage deep active learning strategy, we achieved efficient and reliable optimization in complex multicomponent electrolyte systems, as further validated by our experimental results. We also hope this framework can

attract broader attention from researchers and help speed up the development of electrolytes and other materials.

1. During the knowledge transfer stage, ADI values were calculated to guide further electrolyte design via DAL. Could the authors elaborate on whether these values could also serve as practical indicators for electrolyte chemists, thereby enabling more rational and efficient electrolyte formulation?

Our Response: Thank you for this valuable comment. The ADI value is a statistical measure that links electrolyte formulations with their estimated outcomes in the parameter space. It reflects the overall interaction among components with respect to LMA stability. While it cannot serve as a direct chemical descriptor like solvent polarity, it captures the combined effect of different components. We therefore believe ADI values can help connect electrolyte composition to the resulting lithium metal performance. For example, as discussed in the manuscript, the ADI value of 96 for DME-LiFSI reflects the known stabilizing role of DME together with inorganic SEI formation from FSI^- decomposition, whereas the higher ADI value of 114 for EC-DOL suggests the stabilizing effect of DOL on carbonate-based electrolytes. We have now added further discussion in the manuscript on the potential of ADI as a practical indicator for electrolyte chemistry.

We have now added the discussion to the manuscript as:

Notably, the ADI value provides a statistical perspective for electrolyte chemistry, such as the literature-reported correlation of DME-LiFSI (ADI: 96) and the newly identified correlation of EC-DOL (ADI: 114), offering further insights into electrolyte design.

2. The electrolyte for lithium metal batteries needs to balance the stability of both the lithium metal anode and the cathode. The authors first evaluated lifespan in Li||Li symmetric cells and then extended the exploration to full-cell systems. Would direct application to full-cell configurations be feasible and potentially more efficient?

Our Response: The discontinuous nature of electrolyte properties exists in both Li||Li symmetric cells and full-cell system. Therefore, our DAL method which targets discontinuous structure-property landscapes, can in fact be applied directly to full-cell electrolyte discovery. As shown in our few-shot optimization experiments with full-cells, our two-stage framework also demonstrates efficient exploration. In practice, especially for industrial applications where full-cell performance is of primary concern, electrolyte formulations and full-cell performance data can indeed be directly integrated into our framework for optimization. However, as you noted, the stability of both the lithium metal anode and the high-voltage cathode must be

considered simultaneously. For this reason, in our current study we first separated the problems for clarity, using symmetric cells as a demonstrative case. This allows the optimization process and the DAL-TSC framework to be better understood, while still being extendable to full-cell design.

3. The comparison between TSC-derived knowledge and reported results shows an 85% overlap, but also a 15% discrepancy. Will this discrepancy may influence the effectiveness of subsequent optimization strategies?

Our Response: Thank you for this valuable comment. To further verify the validity of ADI values in electrolyte design, we compared our DAL-derived results with literature-reported electrolytes that have similar component structures. Since the literature data and our ADI values are independent—and the reported studies often differ in testing methods and conditions—some inconsistencies in component interactions are observed (as noted in **Supplementary Data 2**) and were not included in the DAL comparison sequences. The 85% agreement indicates strong consistency between literature statistics and ADI values, while the 15% discrepancy mainly arises from the limited number of statistically meaningful sequences in the literature and from the design of the DAL reward function. Specifically, DAL allocates higher precision to high-ADI interactions (which favor longer lifespan) and lower precision to low-ADI interactions, thereby balancing accuracy and sample efficiency. As shown in **Fig. 4d**, this strategy strengthens the certainty of high-ADI interactions during iterations, so the 85% overlap is reasonable and does not undermine the overall exploration efficiency.

We have now added the discussion to the manuscript as:

Our analysis shows that approximately 85% of previously reported correlations align with our DAL-derived knowledge, validating DAL's ability to generate meaningful insights for electrolyte design, while remaining discrepancies reflect limited literature statistics and the DAL framework's focus on interactions that enhance lifespan.

4. While BO has been applied and discussed, there exist other promising optimization methods (e.g., PHOENICS or other global optimization algorithms). A brief discussion of alternative strategies may broaden the applicability and impact of the proposed framework.

Our Response: Thank you for your comment. While various optimization strategies exist, including Bayesian optimization frameworks like PHOENICS that you mentioned, PSO (Particle Swarm Optimization) and CMA-ES (Covariance Matrix Adaptation Evolution Strategy), they typically handle discontinuous variables through continuous relaxation (e.g., assigning percentages to discrete salt choices) or encoding schemes (e.g., binary variables for

all possible combinations). Such encoding approaches significantly increase the dimensionality of the optimization space, making it challenging to achieve effective optimization with limited experimental samples. Such approaches often increase the dimensionality of the search space and make optimization with limited experimental data less effective. PHOENICS, for example, achieves efficient optimization in high-dimensional continuous spaces via Bayesian kernel density estimation. However, the dynamic nature of electrolyte discovery requires an optimization method that can also perform transfer learning, so that newly introduced molecules can be integrated without discarding accumulated knowledge.

Our two-stage optimization framework addresses these challenges by combining deep active learning with knowledge transfer. This allows efficient exploration of discontinuous and complex electrolyte spaces, reduces experimental cost, and enables adaptive incorporation of new candidate molecules while retaining historical insights. As a comparison, we also applied conventional Bayesian optimization (GP-AL) with the same encoding scheme. Trained on the same randomly selected batches, GP-AL did not significantly improve cycle life (**Fig. R1, Supplementary Fig. 3e**). In contrast, our deep kernel learning-based approach operated effectively in the discrete space and captured complex discontinuities, resulting in a clear improvement in lifespan. This enhanced modeling capacity makes our framework especially valuable in data-limited experimental scenarios.

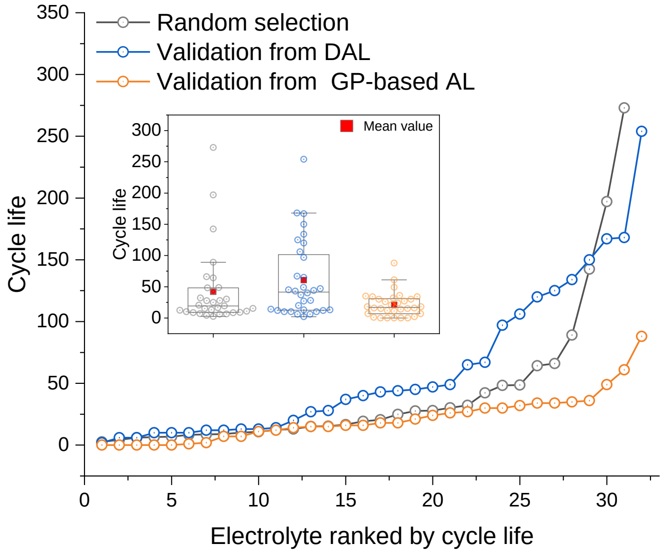

Fig. R1. The experimental validation to compare the DAL and GP-AL methods. Randomly selected data were used as training data for both methods. The experimental electrolytes recommended by the DAL approach (blue) exhibited a higher lifespan compared to those from the GP-AL approach (orange). Inset: statistical result.

We have now added the discussion to the manuscript as:

Compared with the standard Gaussian process-based active learning (GP-AL), our DAL approach can build a more accurate surrogate model for such a rapidly oscillating objective function (**Fig. 2e** and **Supplementary Fig. 3**), as further validated by experimental results (**Supplementary Fig. 3e**).

5. In repeated experimental results, such as those presented in Fig. 3D and Fig. 5C, the standard deviation of the measured lifetimes appears to be relatively large. The author should provide the reasons for such large deviation.

Our response: We appreciate the reviewer for pointing out this important point. We note that the measurement noise, i.e. the large standard deviation, reflects one of the main features of our DAL method which is to deal with large experimental errors over optimization. The possible reasons are:

1) Variations of the potential profiles:

Fig. R2 shows a particular case of high data variability: the potential-capacity profiles of 5 repeated $Li^0|Li^0$ symmetric cells using the same electrolyte. Cells A and B clearly exhibit soft short-circuits, while Cell E experiences a hard short-circuit, *i.e.* a sudden drop of the overpotential. This difference should arise mainly from the unevenness of LMA surface, which can lead to localized dendritic growth at specific points.

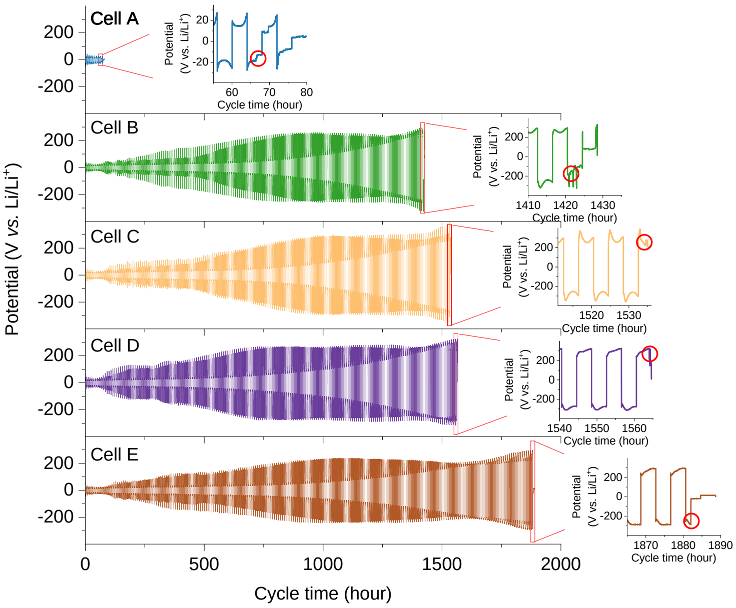

Fig. R2. The detailed voltage profiles for the validating electrolyte (1m LiDFOB DME/ES+5wt.% FEC) with five repeated cells. The red circles indicate short-circuit.

2) Variations of laboratory experimental conditions:

Cell performance measurement variability is a particular challenge for laboratory experiments. For example, even for testing of commercial cylinder batteries, the variability is also large (e.g. *Nature*, 578, 397, 2020; **Fig. R3**). In our work, we have included all generated data, which vividly shows the variability inherent in these tests.

[Figure Redacted]

We have now added the discussion to the manuscript as:

Even when the training samples are deliberately assigned with large noise, generally arising from minor differences and large errors in lithium metal anode in experiments, the DAL approach efficiently identifies the optimal solution as early as the second iteration (**Supplementary Fig. 4**).

6. In exploring a larger parameter space, the authors employed a broader range of components, including two types of salts, three types of solvents, and multiple additives, resulting in 5,400 different electrolyte combinations. What were the practical considerations behind this configuration?

Our response: In principle, the composition of electrolytes can be expanded almost infinitely. In industrial practice, the variety of lithium salts and primary solvents is usually limited to around 6-8 options, largely due to considerations of product consistency and manufacturability. In our demonstration study, the initial parameter space was intentionally constrained to a single lithium salt, two solvents, and either one or no additive, in order to enable rapid optimization with DAL. In subsequent investigations of larger parameter spaces, we expanded the number of lithium salts, solvents, and additives to better reflect practical application requirements.

We have now added the discussion to the manuscript as:

Commercial electrolytes typically contain multiple more than two solvents and additives at varying concentration ratios, creating a parameter space that is prohibitively complex and time-consuming to explore through exhaustive experimental testing.

Reviewer #1 (Remarks on code availability):

The code runs successfully, allowing the main results to be reproduced.

Our response: Thank you for this positive feedback. We are pleased that the provided code enables successful reproduction of the main results.

Reviewer #2 (Remarks to the Author):

The study by Hong et al. presents a deep active learning (DAL) approach for optimization of electrolyte formulations for lithium metal battery (LMB) full cells using deep kernel learning (DKL). The approach was successfully used by the authors to find efficient electrolytes compared to initial electrolytes tested. The authors also developed a target statistic encoding (TSC) framework to quantify learned knowledge and then leverage it to find newer electrolytes with few-shot learning.

The present study provides certain advancements in the data-driven battery materials discovery field and carries out >100 real-world battery testing. However, there are significant limitations and loopholes in the developed approach that should be addressed to make it truly generalizable. Here are detailed comments:

Our response: Thank you for your valuable comments that largely help improve the quality of our manuscript. Below we provide point-by-point responses that clarify challenges of experiment-driven electrolyte optimization, expand on algorithmic details, and supply additional data and analyses requested. We believe these revisions strengthen the manuscript and address the reviewer's concerns.

1. The manuscript revolves around the central idea that their approach can be utilized to perform optimization of electrolyte properties in the discrete electrolyte chemical space as they mentioned it multiple times in the introduction and elsewhere: "… these approaches have been confined to scenarios featuring a numerically continuous space." However, some of the recent works have already been developed utilizing AL framework to carry out optimization of electrolytes in the discrete space, e.g., Noh et al., Nat Commun 15, 2757 (2024); Zhang et al., Mater. Today Energy 51, 101909 (2025). It should be clearly stated along with proper citations of these references. I have further reservations on their "optimization in discrete space" claim, which I will point out below.

Our response: We thank the reviewer for the comment. As you rightly pointed out, optimization of multi-component electrolytes inherently involves two dimensions: (i) the selection of molecular components, which is discrete in nature, and (ii) the adjustment of their ratios, which can be treated as a continuous variable. In the works cited by the reviewer (Noh *et al.*, *Nat. Commun.* 15, 2757, **2024**; Zhang *et al.*, *Mater. Today Energy* 51, 101909, **2025**), both aspects were recognized, but the emphasis was primarily placed on continuous ratio tuning.

- Noh *et al.*, *Nat. Commun.* 15, 2757, **2024**: The parameter space was constructed from binary solvent mixtures across continuous volume fractions, resulting in ~2000 formulations derived from 22 molecular solvents. Because their input data were obtained

from a computationally accessible database, the Gaussian process-based active learning (GP-AL) approach was suitable and effective for ratio optimization. However, in experimentally expensive domains such as ours, we found that GP-based methods are less robust when faced with strongly discontinuous property variations, whereas the DAL framework offers superior adaptability.

- Zhang *et al.*, *Mater. Today Energy* 51, 101909, **2025**: The optimization variables were the molar ratios of LiFSI:DME (X_1) and LiFSI:TTE (X_2), which are continuous in nature. As also confirmed in our **Supplementary Figs. 2d-f**, properties such as ionic conductivity and viscosity typically vary smoothly with concentration, making GP-AL an appropriate tool in such cases.

In contrast, our work focuses on the discrete molecular selection problem, where the choice of individual components often leads to dramatic variations in electrolyte performance. We have now cited these relevant works in the revised manuscript and clarified this distinction. Moreover, our experimental results demonstrate that standard GP-AL methods, which are better suited for continuous spaces, are less effective when applied to this discrete molecular optimization problem, thereby underscoring the necessity of our DAL approach (**Fig. R4.**).

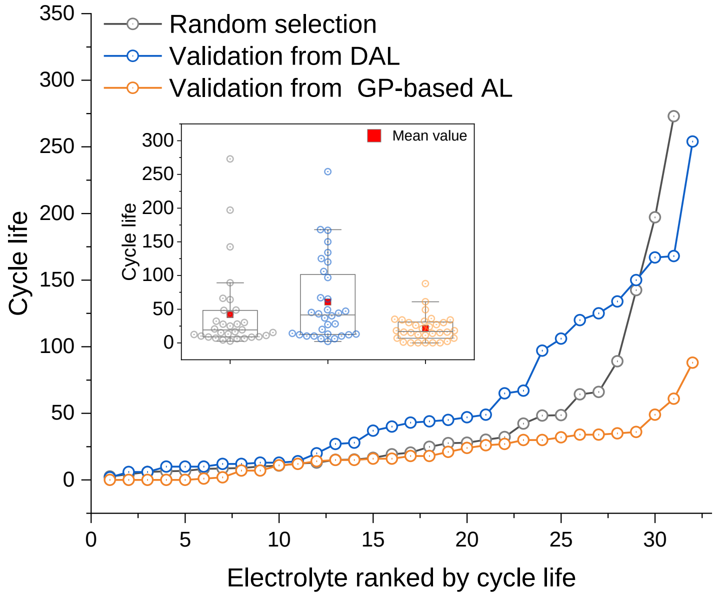

Fig. R4. The experimental validation to compare the DAL and GP-AL methods. Randomly selected data were used as training data for both methods. The experimental electrolytes recommended by the DAL approach (blue) exhibited a higher lifespan compared to those from the GP-AL approach (orange). Inset: statistical result.

We have now added these references to the manuscript:

17. Noh, J. *et al.* An integrated high-throughput robotic platform and active learning approach for accelerated discovery of optimal electrolyte formulations. *Nat. Commun.* **15**,

2757 (2024).

38. Zhang, J. *et al.* Dual-objective optimization of lithium metal battery electrolytes via machine learning. *Mater. Today Energy* **51**, 101909 (2025).

2. The authors also mention "large experimental variability" in reporting battery testing metrics such as cycle life, which is certainly true and is also apparent from Figures 3 and 5. So, I commend the authors for reporting such true and large variations in the manuscript, which is seldom reported in the literature. But my question is how to predict such large measurement noise, i.e., aleatoric uncertainties? A Bayesian technique such as DKL can be used to predict such aleatoric uncertainties, so the authors should check if their DKL model can indeed reproduce the noise in their measurements. It will be highly beneficial to the community.

Our response: We thank the reviewer for this insightful comment and for highlighting the importance of reporting large experimental variability. We agree that this aleatoric uncertainty is an inherent and critical aspect of battery research that warrants more attention.

The central design principle of our work is to accelerate materials discovery via Bayesian framework. Consequently, its primary function is to efficiently guide the search for high-performing electrolyte formulations rather than to explicitly model or reproduce the statistical distribution of the experimental noise.

Nevertheless, aleatoric uncertainty is implicitly managed within our Gaussian process with deep kernel learning (GP-DKL) approach. The GP-DKL surrogate models the predictive posterior distribution over the design space, and its variance term inherently captures the uncertainty associated with its predictions. Our Thompson sampling-based acquisition function leverages this full posterior distribution—not just the mean prediction—to balance exploration and exploitation. By sampling from the surrogate, the algorithm naturally accounts for regions of high uncertainty, which are influenced by both a lack of data (epistemic uncertainty) and inherent measurement noise (aleatoric uncertainty). This optimization-focused approach is particularly well-suited to our application context, where experimental evaluation of electrolyte performance presents significant challenges: each sample requires ~3 months of testing time and is subject to considerable experimental variability.

We agree with the reviewer that a direct assessment of the DKL model's capability to reproduce the observed aleatoric uncertainty would be a valuable contribution and an interesting direction for future research. However, for the specific objective of this work—accelerated optimization under severe experimental constraints—the key performance metric is the efficiency of discovering optimal candidates. Therefore, the success of our framework is best measured by its demonstrated ability to rapidly identify promising electrolyte formulations with minimal experimental overhead, as shown through our active learning iterations and the

successful knowledge transfer.

2. The authors did not open source their codes, which is one of the requirements of the journal. While they did provide access to their partial codebase, I would also need access to data, model checkpoints and remaining codes in order to ensure it is fully reproducible. The provided codebase is also not well-documented. So, I recommend that the authors upload all their codes, datasets, and model checkpoints on GitHub and/or Zenodo with detailed documentation on how to use it including the python libraries involved.

Our response: Thank you for your comment. All the codes and datasets have been uploaded to GitHub (https://github.com/XWang1999/Electrolyte_Optimization_Deep_Active_Learning) with detailed documentation specifying the Python library dependencies and step-by-step instructions for reproducing the optimization workflow.

- Our framework is designed as an online optimization process that operates in tandem with sequential experimental measurements, where each experimental iteration informs the next candidate selection. Accordingly, the released repository includes a demonstration notebook (*Electrolyte_Optimization_Demo_DAL.ipynb*) that reproduces the full optimization workflow using three active learning iterations, providing a transparent and executable example of how the method operates in practice.
- The Deep Kernel Learning (DKL) implementation is provided in the *dknet* folder, which details the construction and training of the hybrid neural network–Gaussian process surrogate. In addition, a numerical example implemented in *DAL_numerical_demo.py* illustrates the optimization process on a benchmark test function, offering a clear demonstration of how the DKL-based surrogate performs iterative learning and guides the search toward optimal regions.
- For optimization in expanded parameter spaces, the framework can be directly extended by modifying the Target Statistic Coding (TSC) module according to the annotations and explanations provided in the notebook. This procedure enables knowledge transfer between tasks and has been explicitly described in both the manuscript and the released code, ensuring that the workflow can be readily adapted for broader electrolyte design and related optimization scenarios.

3. Why choose Thompson sampling as acquisition function over standard and more common acquisition functions such as the expected improvement? There is no justification provided in the draft.

Our response: We selected Thompson Sampling (TS) over standard acquisition functions like Expected Improvement (EI) primarily for its superior and more straightforward scalability for

batch-parallel Bayesian Optimization [R1]. This is a critical requirement by our experimental hardware.

Our experimental platform is a 32-channel battery cycler, which allows us to test 32 distinct electrolyte formulations simultaneously. To fully leverage this capability and accelerate the optimization process, we need select a diverse and promising batch of candidates at each iteration, rather than a single point.

Thompson Sampling is well-suited for this batch scenario. To generate a batch of k candidates (in our case, k=32), we can simply draw k independent function samples from the posterior of the Gaussian Process surrogate model. The location of the maximum of each sample is then chosen as a candidate for the batch. This procedure is computationally efficient and naturally promotes exploration and diversity within the batch, as each sample represents a different plausible hypothesis of the true objective function.

In contrast, extending acquisition functions like EI to the batch setting is more complex. A naive approach of simply selecting the top k points with the highest EI values would result in a highly redundant batch, with points clustered around a single peak in the acquisition function. More sophisticated methods like q-Expected Improvement (q-EI) are required to properly account for the joint improvement over a batch, but they come at a much higher computational cost and implementation complexity [R2].

Given our experimental constraint of performing 32 parallel evaluations, Thompson Sampling provides an elegant, scalable, and effective solution for maximizing the efficiency of our discovery workflow.

To improve the justification, the text below has been inserted into the "Deep active learning approach" Section of the revised manuscript: "To fully leverage the experimental throughput of parallel evaluations, an acquisition function of Thompson Sampling (see **Methods**) is then constructed based on the posterior distribution from the surrogate model of DKL. This strategy enables scalable batch-parallel optimization and efficiently sample the parameter space of electrolyte formulas by balancing exploration (i.e., testing regions with high uncertainty) and exploitation (i.e., testing promising regions based on completed experiment results) [50]"

[R1] Kandasamy, K. *et al.* Parallelised Bayesian optimisation via Thompson sampling. *International Conference on Artificial Intelligence and Statistics.* 133-142 (2018).

[R2] Gelbart, M.A. Constrained Bayesian Optimization and Applications. Harvard University, 2015.

4. a) The optimization is carried over only 13 molecular components (6 solvents, 4 salts, 3 additives) and later two more molecular components were added in the search space. While we know that molecular chemical space is enormous, estimated to be on the order of 10^{60}. So, is their approach truly carrying out optimization over discrete electrolyte space then?

Our response: Thank you for this important question, which touches on a crucial distinction. The reviewer is correct that the universal molecular chemical space is astronomically large. Our work, however, is not designed to address that ***molecular generative space.*** Instead, it targets the ***formulation compositional space***, which is a practical and important challenge in experimental battery research.

This compositional space arises from the combinatorial mixing of a pre-selected set of viable components (solvents, salts, and additives). The primary bottleneck here is not computational but experimental. Each data point—representing a single electrolyte formulation—requires complex cell assembly, pre-conditioning, and ***months-long cycling tests to evaluate performance.*** Furthermore, these battery-level evaluations suffer from ***significant experimental noise***, where identical formulations can yield considerable variations in cycle life. Because of this extreme cost and inherent variability, typical experimental studies report only a handful of formulations.

While our initial set of 13 components is modest, it generates a combinatorial space of over 5,400 unique formulations—a number far too large to screen exhaustively via brute-force experimentation. The addition of just two new components expands this space beyond 10,000, demonstrating the combinatorial explosion that necessitates an intelligent search strategy. Our framework was specifically developed to navigate this ***discrete, noisy, and expensive-to-evaluate space,*** providing a practical bridge between theoretical chemistry and real-world experimental constraints.

b) The search space is also very small – 720, 5400 and 5726. Do we really need AL for such small spaces? AL is usually carried over large search spaces on the order of thousands, if not millions, of input samples.

Our response: We agree with the reviewer that these search spaces are not large in a “big data” context. However, the need for Active Learning (AL) in our work is dictated not by ***the absolute size of the search space***, but by the ***prohibitive cost of each experimental evaluation***.

In this domain, our total “experimental budget” is severely limited. Each sample requires months of testing and occupies a channel on our specialized equipment. Therefore, our primary objective is to achieve maximum ***sample efficiency***—to locate the optimal formulation in the fewest possible experimental steps.

This is precisely the scenario where AL excels. AL is a powerful tool for problems with a high query cost, where it intelligently selects the next most informative experiment to run. By sequentially learning and updating its model of the landscape, it minimizes the number of costly evaluations needed to converge on a high-performing candidate. The goal is not to exhaustively search the space, but to find the optimum with a budget of just a few dozen experiments. Our two-stage framework further enhances this by allowing the incorporation of new molecules without restarting the entire process, directly addressing a key limitation in practical research.

Thus, the use of AL is not only justified but essential for making progress in a field constrained by such severe experimental bottlenecks.

c) A frequentist ML model can well be utilized to find optimal candidates in such small search spaces.

Our response: While a frequentist ML model could certainly be trained on a completed dataset to predict performance, it is fundamentally the ill-suited tool for the task of **sequential experimental design**, which is the core of our work.

The critical distinction is that a standard frequentist model provides a **point estimate** (a single predicted value), but it lacks a measure of its own **predictive uncertainty**. In a data-scarce and expensive discovery campaign like the electrolyte discovery problem, this uncertainty is arguably more important than the prediction itself. It is the uncertainty that answers the most crucial question: **"Which experiment should I run next to find the optimum most efficiently?"**

Our Bayesian active learning framework is designed to answer exactly that question. By maintaining a full posterior distribution, it quantifies the model's confidence across the entire search space. This uncertainty is fed into our Thompson sampling acquisition function, which naturally balances two critical needs:

- **Exploration**: Testing in regions where the model is uncertain but the potential could be high.
- **Exploitation**: Refining candidates in regions already known to be promising.

A frequentist model, lacking this uncertainty, cannot perform this intelligent exploration and would resort to a purely exploitative or random search, leading to lower sample efficiency and a higher risk of converging to a local optimum. Therefore, we chose a Bayesian approach not just as a preference, but as a necessity for enabling the sample-efficient, guided search required to solve this problem with high cost of each experiment.

5. In Figure 2e, I believe the comparison between GP-AL and DAL is made using the RBF covariance kernel in GPs. While it is true that RBF is suited for smooth and continuous

functions, there are also other standard covariance kernels that can be used when there is roughness in the functions, e.g., Matern, rational quadratic, pairwise, etc. Multiple covariance kernels can also be combined together to achieve the desired effect. So, their choice of using DKL over GP in the present study is not well justified. They should compare GPs with aforementioned covariance kernels to truly establish the superiority of DKL over GPs in such scenarios.

Our response: We thank the reviewer for the comment and agree that the choice of covariance kernel critically affects the performance of Gaussian process (GP) models. In Figure 2e, the baseline GP-AL results were shown with the radial basis function (RBF) kernel, which is a standard default due to its smoothness assumptions and wide use in Bayesian optimization. Importantly, our comparison was not restricted to this case. In additional numerical studies, we explicitly tested GP models with alternative kernels, including the Matern class, as reported in **Supplementary Figs. 3** and **4**. While Matern kernels provided some improvement over RBF in certain synthetic benchmarks, our DAL framework consistently achieved superior performance across scenarios, indicating that the observed benefits are not an artifact of kernel choice.

Beyond direct comparisons, the broader motivation for adopting DKL lies in the challenge of kernel specification in our application domain. Each kernel encodes specific assumptions about the underlying functional landscape—for example, RBF assumes infinitely differentiable smoothness, while Matern allows controlled roughness. However, in electrolyte optimization the true correlation structure among solvents, salts, and additives is highly complex and not known a priori. Selecting and tuning a fixed kernel, or combining multiple kernels, requires strong manual assumptions and extensive heuristic effort, which risks mis-specifying the model. By contrast, DKL provides an adaptive mechanism to learn effective kernel representations directly from data, enabling the model to flexibly capture the relationships among solvents, salts, and additives without relying on predetermined smoothness assumptions. This adaptivity is particularly valuable given the high experimental variability and the complex interactions among electrolyte formulations in our system.

To improve the justification, the text below has been inserted into the "Deep active learning approach" Section of the revised manuscript: "On one hand, the deep neural network can learn structure-property relationships directly from data, automatically forming adaptive kernel representations and thereby avoiding the need for manually defined covariance kernels with predefined smoothness assumptions, which enhances the representation ability of models."

6. Along the same lines, the authors mention that "…the black-box functions with rapid oscillations, which are characteristic of the objective function in the electrolyte optimization

problem…" But it has not been explicitly shown that the objective function for electrolyte optimization problem is indeed "rapidly oscillating".

Our response: By "rapid oscillations," we refer to the large parameter–performance discontinuities observed in our experimental data. As shown in **Supplementary Fig. 2** (here cited as **Fig. R5** for readability), even when varying only one component (e.g., additive, lithium salt concentration, salt identity, or solvent), the resulting cycle life shows sharp and non-monotonic changes, rather than smooth trends. For instance, in **Supplementary Figs. 2b**, **h**, and **k**, small changes in composition can lead to either significant improvement or drastic deterioration in lifetime, with no gradual transition. This confirms that the objective function in electrolyte optimization is indeed highly non-smooth and discontinuous. To improve clarity , we have revised the manuscript to explicitly connect the term "rapid oscillations" with the experimental evidence presented in **Supplementary Fig. 2**.

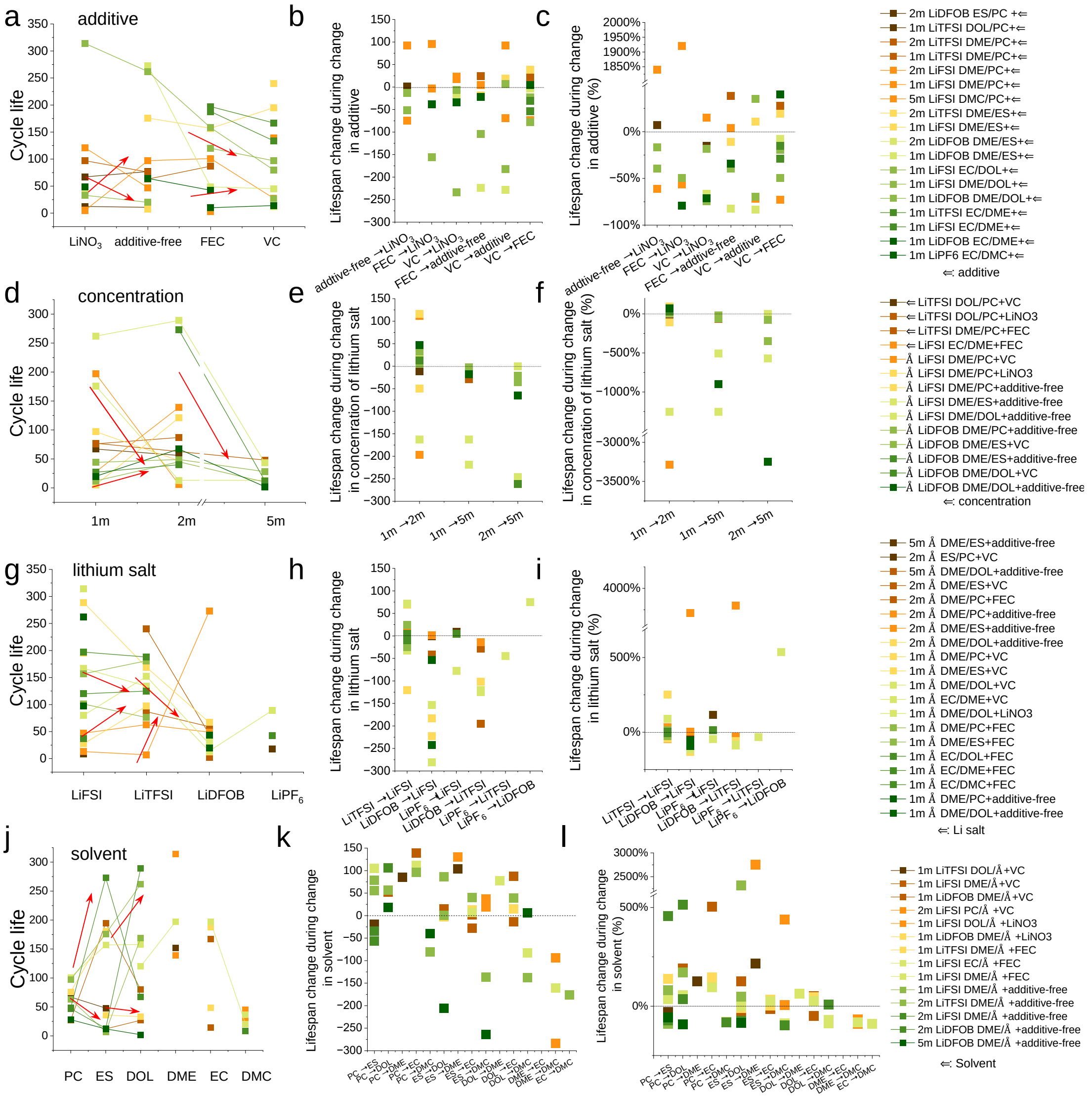

Fig. R5 | Experimental verification of the large parameter-performance discontinuity (leading to the discreteness of the parameter space) in relating the electrolyte components with the cell cycle life. A variable-control statistical method was here used to analyze the change of cells' cycle life as one single component is changed in the electrolyte: **a-c**: change of only the additive, **d-f**: change of only the lithium salt concentration, **g-i**: change of only the lithium salt, **j-l**: change of only one solvent. All the data points analyzed here are from the experimentally measured electrolyte formulas during the three DAL iterations. To illustrate the large parameter-performance discontinuity, for each scenario of component change (that is, each row), we plot the raw data of absolute values of cells' cycle life for each electrolyte (left column, **a, d, g, j**), the change of absolute values of cells' cycle life as the component changes from one to another (central column, **b, e, h, k**) and the corresponding relative ratio of change of cells' cycle life (%, right column, **c, f, i, l**). The color-coding legend of each group of data points is shown on the very right, where the square symbols represent the components to be changed. For the figures in the central and right column, the x-axis shows the specific component that is changed, and each change contains multiple data points with each point representing a specific electrolyte (shown as colored squares).

We have now added the discussion to the manuscript as:

As illustrated in **Supplementary Fig. 2,** the space of electrolyte formulas is discrete in the dimensions of Li salts, solvents and additives, but somewhat relatively continuous in salt concentrations (**Supplementary Figs. 2d-f**), which points to an objective function featuring both discontinuous transitions and continuous variations. This sharp and non-monotonic response of cycle life to single-component changes demonstrates the "rapid oscillations" in the objective function that characterize the electrolyte optimization problem. Therefore, a numerical function of $f = (1 - |x|)\sin(20(1 - |x|)^6)$ was set up to simulate the process to verify the efficacy of the DAL approach based on the surrogate model of DKL (**Fig. 2e** and detailed in **Methods**).

7. The authors also make claims about DAL framework being able to balance between exploration and exploitation. This can be explicitly shown on a reduced latent space representation of electrolytes from the trained DKL model by showing suggestions from different batches. If the model is indeed exploiting, then the suggestions from later batches should converge to a particular region of the chemical space. The authors can also show distribution of uncertainties for suggestions from different batches and if the model is progressing towards exploitation, then the distribution of uncertainties should shift towards lower values.

Our response: Thank you for your comment. In fact, the dynamics of balancing exploration and exploitation during the DAL optimization are already reflected in **Supplementary Fig. 5** (here

cited as **Fig. R6** for readability). Specifically:

First, iteration improvement (**Fig. R6a** and **b**): The cycle life of the tested electrolytes shows consistent enhancement across iterations, with progressively diminishing gains (**Fig. S6b**). This trend demonstrates that the model initially explores a broader parameter space but gradually shifts towards exploitation by converging to high-performing regions.

Second, exploration-exploitation balance (Fig. R6c): The overlap of the suggested candidates between the third and fourth iterations (orange points in the red box) highlights exploitation, as the model repeatedly focuses on promising regions. Meanwhile, the blue points with relatively high uncertainty illustrate the continuing element of exploration, maintaining diversity in the search.

Together, these results confirm that the DAL framework naturally achieves a balance between exploration and exploitation, consistent with the reviewer's expectation.

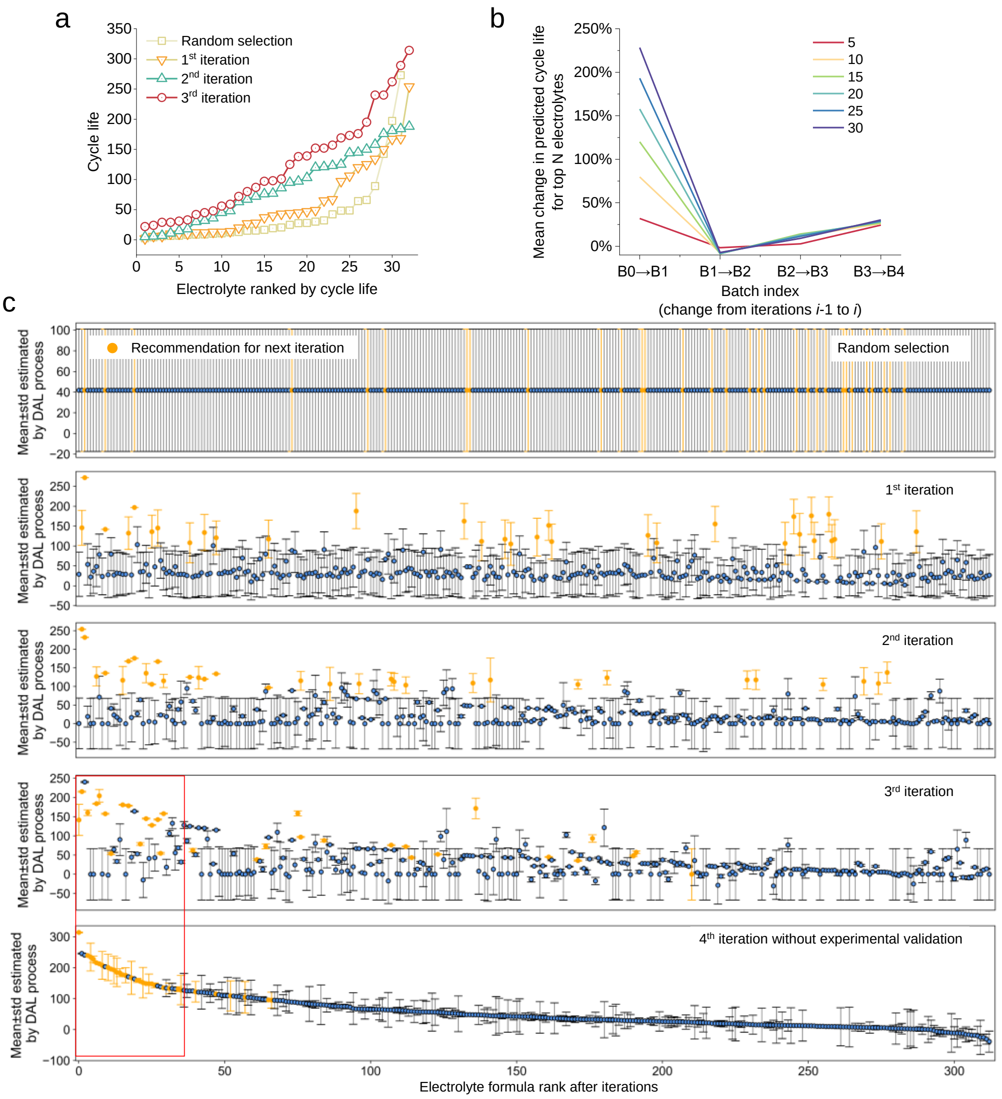

Fig. R6| The results of iterations and termination analysis for the DAL optimization process performed in this study. a, The experimental results of cells' cycle life of measured electrolytes in the initial random selection round and the three iterations, ranked by the cells' cycle life. The data points shown here are the same as those shown in **Figs. R6a, b** (which are plotted differently). We can clearly observe that overall, the cells' cycle life exhibits a continuous improvement over the three iterations, particularly for the range with cell cycle life of over 100 cycles. The illustration here corroborates the results shown in **Figs. R6a, b**. **b**, The relative change (%) of the mean of estimated cells' cycle life (including top N data points) between consecutive iterations, wherein the differently colored lines are calculated based on the data of the top N longest cell cycle life (N= 5, 10, 15, 20, 25, 30). Clearly, as the iteration advances, the change of cells' cycle life between iterations progressively diminishes, highlighting the gradually optimized performance by DAL. **c**, The mean and standard deviation (mean ± std) of the predicted cells' cycle life for each electrolyte after each iteration, ranked by average of the cycle life (not by the numbering of electrolytes). Note that no experimental validation was performed for the 4th iteration. The electrolyte formula rank after each iteration displays predicted cell cycle life of each electrolyte (electrolytes with longer life are listed on the left side of the figure and those with shorter life on the right). The blue points represent predicted electrolytes and their performances in the current iteration, and the orang points represent the electrolytes that are to be validated for the next iteration. As the iteration advances, we can see that the points of evaluation for next iteration (yellow points) in general shift towards the left, highlighting the DAL's ability to identify parameter space regions of electrolytes with long cell cycle life. Further, there is a large overlap between evaluation points of the fourth and third iterations (orang points highlighted in the red boxed area with relatively longer predicted cell cycle life). In contrast, for the blue points highlighted in the red box area, the cell cycle life exhibits a higher uncertainty or relatively higher mean values in the third iteration, indicating a call for balance between exploration and exploitation.

8. How are the molecular components represented? I do not see any discussion in the Methods section. Do they represent molecules using Morgan fingerprints or RDKit features or graph neural networks (GNN) or some sort of one-hot encoding? If one-hot encoding is used to represent molecular components in the electrolytes, then their model cannot really be inferred on molecular components unseen by the DKL model. If that's the case, then the claim of "optimization over discrete electrolyte space" again does not really hold true.

Our response: We thank the reviewer for raising this point. Representation of molecular components is indeed central to our framework. We acknowledge that molecular fingerprints (e.g., Morgan), RDKit features, and graph neural networks (GNNs) are powerful tools for cheminformatics, as they encode detailed structural and physicochemical information. However, these approaches are fundamentally designed for large-scale datasets. They rely on thousands

or even millions of labeled molecules to learn stable representations and to generalize robustly to unseen structures. In contrast, electrolyte discovery operates under radically different conditions: each data point requires weeks to months of electrochemical testing, experimental reproducibility is limited, and datasets are inevitably small, sparse, and noisy. These constraints make the aforementioned complex representations impractical, leading to inaccurate models and a high risk of overfitting. . Our approach therefore emphasizes a representation strategy that is both experimentally feasible and aligned with the discrete, high-cost nature of electrolyte optimization. Specifically:

(1) **Representation strategy.** In practice, we do not rely on simple one-hot encoding, which would restrict the model to a closed set of molecules and prevent generalization. Instead, each electrolyte formulation is represented by relational features that capture pairwise, triplet, and higher-order interactions among salts, solvents, additives, and concentrations. These features are quantified using the average degree of interaction (ADI), defined as the mean cycle life across all tested formulations containing a given component combination. For example, the ADI between DOL and EC reflects the average cycle life measured across all electrolytes that include both solvents. This representation embeds experimentally derived interaction knowledge into the feature space and can be incrementally refined as additional data or new components are incorporated. Such adaptability is integral to our two-stage framework, in which knowledge obtained from smaller, well-characterized spaces is systematically transferred to larger or newly expanded spaces, thereby ensuring both scalability and continuity of optimization under realistic experimental conditions.

(2) **Why not molecular fingerprints or GNNs.** These molecular representations are indeed powerful tools in cheminformatics, as they encode detailed structural and physicochemical information and can, in principle, generalize to unseen molecules. However, their effectiveness relies on access to large, diverse, and well-labeled datasets. In electrolyte discovery, each new data point requires costly preparation and long-term cycling tests (often extending over weeks or months) making the collection of datasets at the scale required for graph- or fingerprint-based approaches impractical. Our study instead targets discrete electrolyte formulations, where the dominant challenge is to extract meaningful knowledge from scarce but high-value data. Relational features derived directly from experimental outcomes provide a more practical and chemically aligned representation, as they capture transferable interaction knowledge that can be reused when the search space expands. This allows subsequent optimization to fully leverage prior insights rather than restarting from scratch, thereby improving efficiency under realistic experimental constraints.

(3) **Handling unseen components.** We agree with the reviewer that predicting performance for entirely new molecules is inherently challenging. Our framework is not intended for zero-

data prediction of unseen components, but rather supports few-shot transfer. When a new solvent, salt, or additive is introduced, a limited number of targeted experiments are conducted to estimate its pairwise ADI values with existing components. Once these priors are established, the model can incorporate the new component into the optimization process while leveraging the interaction knowledge already captured from the existing component library. **Importantly, this adaptive mechanism allows the framework to expand iteratively over time, incorporating new molecules and enlarging the search space without the need for complete retraining.**

9. I also have reservations about their developed TSC approach. The average degree of interaction (ADI) values from this TSC approach can only be obtained post training of the DKL model and is not learned during the training itself. This limits the utility of this approach. Also, continuing from previous point, one needs to know the ADI values of unseen molecules a priori in order to make predictions: "For TSC coding the initial ADIs of DEP-or TO-related interactions were assigned as the prior p, which corresponds to the mean values of existing interactions." This will severely limit the applicability of their approach on finding electrolytes constituting unexplored solvent, salts, or additives, as one would need to first perform real-world experiments using these unexplored molecular components.

Our response: We thank the reviewer for this important comment. The reviewer is correct that the Average Degree of Interaction (ADI) values are calculated post-training. ADI is not intended to be a predictive feature learned by the model itself, but rather a post-hoc abstraction of the knowledge captured by the trained DKL surrogate. It serves to distill the complex, non-linear component interactions into an interpretable and transferable format.

The core of the reviewer's concern is how to handle entirely new molecules. Our framework is designed for efficient few-shot optimization, not for zero-shot prediction, which remains a largely unsolved problem in experimental chemistry. When introducing a novel component, we initialize its interaction values with the mean ADI of existing components. This approach provides an informed, neutral prior—a more principled starting point than a random guess—from which the active learning loop can begin.

The key is that this initial prior is not the final word; it is the starting point for exploration. The framework is designed to leverage this prior to select the most informative initial experiments involving the new molecule, allowing it to learn that molecule's specific interaction profile with maximum efficiency. **<u>This stands in sharp contrast to conventional methods, which would require restarting the optimization from scratch and discarding all previously acquired knowledge.</u>**

The practical power of this strategy is demonstrated in our results. As shown in **Fig. 5b**, our framework successfully identified a high-performing formulation containing new molecular components within a single iteration of active learning. This result validates that the TSC-based knowledge transfer, even when initiated with a simple prior, dramatically accelerates discovery in expanded chemical spaces.

We have inserted the below text into the "Generalization ability of DAL for optimizing full cells" Section of the revised manuscript to clarify this point: "This demonstrates that our DAL framework can effectively transfer previously acquired knowledge to guide electrolyte design involving newly introduced molecular components. By initializing unknown interactions with statistically derived priors and incorporating only a small number of experimental measurements, the framework dynamically expands the searchable space, enabling efficient optimization as new molecules are discovered without restarting the process."

10. Other non-technical comments:
a) The introduction section is a bit too long. Even though there is no word limit in the Nature Communications journal, I think it contains redundant information and so can be easily reduced to two-thirds.

Our response: Done as suggested. Please refer to the Introduction Section (changes are marked in red) of the revised manuscript for the details.

b) Some of the sentences are also too long and are hard to follow, e.g.:
"To complicate it further, unlike the optimization of nonchemical (particularly numerical) subjects of interest – the examples of which include battery fast-charging protocols, and physical properties of electrolyte (solubility and concentration, ionic conductivities and viscosity) that vary over a continuous space of numbers – the materials space for electrolyte design is highly discrete, because the component-performance relationship is highly sensitive to many equilibria and inequilibria processes existing in the cell environment, such as the interphasial chemistry and related properties, where the change of a single chemical or its concentration may trigger an exponential increase in potential component interactions, resulting in the combinatorial explosion of search space."

Our response: Thank you for pointing this out. We have revised this sentence, and conducted a thorough review of the full text and revised other similarly lengthy expressions accordingly.

"To complicate it further, unlike the optimization of nonchemical (particularly numerical) subjects of interest — for example, battery fast-charging protocols or tuning electrolyte properties of solubility, conductivity, and viscosity—the materials space for electrolyte design

is highly discrete. This is because the relationship between components and performance is extremely sensitive to many equilibria and inequilibria processes existing in the cell environment. As a result, even a single component change can introduce exponential shifts in chemical interactions, leading to a combinatorial explosion of the design space."

c) Some of the words used in the draft can be replaced with more suitable ones, e.g., "recommendation" with "suggestion" since the word recommendation gives the idea that a recommendation system is being utilized to get new suggestions in each batch, which is not the same as an AL framework.

Our response: Done as suggested. The changes have been highlighted in red in the revised version.

d) Add a separate "Discussion" section in the revised draft (which is allowed as per journal's guidelines) clearly highlighting limitations of their approach as pointed out above.
Our response: Thank you for your comment. A new "Discussion and outlook" section has been inserted into the revised manuscript to clearly highlight the limitations of the proposed approach as pointed out above, which is reproduced below for your convenience.

Discussion and outlook

In this work, the two-stage optimization framework is developed in response to practical challenges, such as the high experimental cost, limited data availability, and inevitable data noise in electrolyte research. As an experiment-driven framework, improving the treatment and even prediction of data noise through the DKL model will be an important yet often overlooked direction for making experimental data more reliable and informative. Looking forward, with the advancement of experimental automation and the accumulation of broader databases, more sophisticated methods—such as molecular encoding, which requires richer datasets—can be further integrated into our DAL optimization framework. Importantly, the discreteness of electrolyte formulations remains intrinsic; thus, with more comprehensive and large-scale databases, the development of adaptive algorithms and optimization frameworks will become feasible. In addition, the TSC-based knowledge transfer serves as a critical tool for quantifying electrolyte formulation design. It provides a pathway to map electrolyte molecular chemistry onto electrochemical performance, offering quantifiable and comparable metrics that can benefit electrolyte chemistry and formulation research.

Reviewer #2 (Remarks on code availability):

Comments on code availability have been discussed in the overall comments to the authors.
Our response: Thank you for your comment.

Reviewer #3 (Remarks to the Author):

The author describes a deep active learning approach to discover new electrolytes for lithium metal batteries. Overall, the work is well structured and clearly presented. I believe that the novelty lies in the combination of deep learning and active learning. However, the author primarily focuses on convincing readers of the importance of this technique for improving the efficiency of electrolyte screening for lithium metal batteries. The main conclusions of the work appear somewhat scattered. I suggest that the author highlight the key novelty of the study more explicitly. I have some concerns for the author to consider, listed below:

Our response: Thank you for your valuable comments that largely help improve the quality of our manuscript. As suggested, we have revised the manuscript to more clearly highlight the key novelty and contributions of this work.

1.The work illustrated in the paper is too specific, and I can imagine that the author spent substantial time completing the experiments. How, then, can the author convince us that this method truly reduces time costs?

Our response: We thank the reviewer for this comment and appreciate the recognition of the experimental effort involved. The experimental validation in this study consists of two stages:

In the first stage, we conducted three rounds of active learning iterations within an initial space of 720 electrolyte compositions, with each round testing only 32 electrolyte formulations. This setup was deliberately designed to reflect realistic laboratory constraints, where exhaustive testing of the combinatorial design space is impractical.

In the second stage, we performed targeted experimental validations in extended chemical spaces, including zero-shot predictions, one-round optimization in full-cell configuration, and an iteration involving new molecule integration via the DAL-TSC framework.

Although the overall experimental process took time due to the nature of battery testing (each round requiring ~3 months), our method was able to identify high-performance electrolyte formulations **using only ~4.4% of the experimental resources** compared to traditional grid-search approaches. This demonstrates the efficiency of our framework in significantly reducing the experimental workload. More importantly, substantial time was saved in the optimization phase once the discrete nature of electrolyte performance was identified and the DAL framework was in place.

2.The performance of lithium metal batteries is important; however, it is still not sufficient to represent or transfer the overall performance to other devices. Thus, the experimental design is critical. LMO is not a typical cathode for lithium metal batteries—why not use NCM cathodes for validation?

Our response: Thank you for pointing this out. We agree that the relationship quantified by the current optimization for Li|LMO cells cannot be directly transferred to other cathodes, because they have different chemical requirements. We did not mean to achieve this goal as it is fundamentally impossible to do this. Our goal is to develop a two-state DAL framework that combines the algorithm and chemical experiments. We can transfer the framework to optimization of other systems but we just need to input different set of experiments.

We apologize for the confusion caused by our terminology. To clarify, **in our full-cell validation experiments, we indeed used NCM811 ($LiNi_{0.8}Co_{0.1}Mn_{0.1}O_2$)**, which is a widely adopted layered metal oxide cathode for lithium metal battery studies due to its high energy density and practical relevance. In the manuscript, we referred to it as a "layered metal oxide", which might have been misinterpreted as spinel-type $LiMn_2O_4$, a different cathode chemistry. We have revised the manuscript to clearly indicate the use of NCM811 to avoid confusion.

3.The authors need to emphasize the importance and necessity of DKL and Thompson sampling by comparing them with the standard BO process.

Our response: We thank the reviewer for this valuable comment. The use of deep kernel learning (DKL) and Thompson sampling (TS) in our framework is a methodological necessity dictated by the unique characteristics of electrolyte discovery in this work. Conventional BO methods—based on smooth surrogate functions and sequential sampling—are often effective for continuous, well-behaved optimization landscapes. However, electrolyte design exhibits fundamentally different characteristics. As shown in Supplementary Fig. 2, even a small variation in formulation (e.g., changing an additive, lithium salt concentration, or solvent) can lead to sharp, non-monotonic changes in cycle life, resulting in a highly discontinuous and multimodal objective surface. These characteristics violate the smoothness assumptions inherent to standard Gaussian Process (GP) models.

Moreover, our experimental platform that is a 32-channel battery cycler allows simultaneous testing of multiple electrolyte formulations, making large-batch parallel exploration essential to accelerate the optimization process optimization under costly and time-intensive evaluations. Our adoption of DKL and TS is thus driven by both the need for an adaptive mechanism to learn effective kernel representations and efficient large-batch parallel exploration, as elaborated below.

Deep kernel learning (DKL). Standard GP-based BO assumes a stationary kernel such as the radial basis function (RBF), which encodes infinitely differentiable smoothness. This assumption becomes restrictive when modeling the discrete and compositionally nonlinear landscape of electrolyte formulations. DKL mitigates this limitation by introducing a learnable kernel structure:

$$k_{\mathrm{DKL}}(\boldsymbol{x}, \boldsymbol{x}' | \boldsymbol{\omega}, \boldsymbol{\theta}) = k_{\mathrm{base}}(g(\boldsymbol{x}|\boldsymbol{\omega}), g(\boldsymbol{x}'|\boldsymbol{\omega})|\boldsymbol{\theta}) \quad (1)$$

where $k_{\mathrm{base}}(\boldsymbol{x}, \boldsymbol{x}')$ denotes the base kernel function, $\boldsymbol{\theta}$ is the hyperparameter of the base kernel, and g is the nonlinear mapping learned by the deep neural network, which is parameterized by the weights $\boldsymbol{\omega}$.

By maximizing the marginal likelihood of DKL, we learn all deep kernel hyperparameters, including the parameter $\boldsymbol{\omega}$ of the deep neural network and the parameter $\boldsymbol{\theta}$ of the base kernel. ***The kernel function in (1) achieves adaptive learning from the data*** by leveraging the flexible representational capabilities of deep neural networks while maintaining the robust uncertainty quantification inherent to Gaussian processes. This stands in contrast to standard GP methods that use traditional kernels, which are constrained by predefined similarity metrics. We demonstrate the superior effectiveness of our proposed DKL approach in **Fig. 2D** of the revised manuscript, where it successfully captures the complex relationships in electrolyte optimization that elude conventional kernels.

Thompson sampling (TS). Efficient parallel exploration is a critical requirement imposed by our experimental workflow. Each experimental round is costly and time-consuming, yet our platform enables simultaneous testing of multiple electrolyte formulations. To fully leverage this capability and accelerate the optimization process, we need to select a diverse and promising batch of candidates at each iteration rather than a single point.

Thompson Sampling is well-suited for this batch scenario. To generate a batch of k candidates (in our case, k=32), we can simply draw k independent function samples from the posterior of the Gaussian Process surrogate model. The location of the maximum of each sample is then chosen as a candidate for the batch. This procedure is computationally efficient and naturally promotes exploration and diversity within the batch [R1], as each sample represents a different plausible hypothesis of the true objective function. In contrast, extending the commonly used acquisition functions in standard BO like Expected Improvement (EI) to the batch setting is more complex. A naive approach of simply selecting the top k points with the highest EI values would result in a highly redundant batch, with points clustered around a single peak in the acquisition function. More sophisticated methods like q-Expected Improvement (q-EI) are required to properly account for the joint improvement over a batch, but they come at a much higher computational cost and implementation complexity [R2]. Given our experimental constraint of performing 32 parallel evaluations, TS offers an elegant, scalable, and effective acquisition strategy for maximizing the efficiency of our discovery workflow.

[R1] Kandasamy, K. *et al.* Parallelised Bayesian optimisation via Thompson sampling. *International Conference on Artificial Intelligence and Statistics.* 133-142 (2018).

[R2] Gelbart, M.A. Constrained Bayesian Optimization and Applications. Harvard University, 2015.

To address this comment, the text below has been inserted into:

- the second paragraph of the "Deep active learning approach" Section of the revised manuscript: "On one hand, the deep neural network can learn structure-property relationships directly from data, automatically forming adaptive kernel representations and thereby avoiding the need for manually defined covariance kernels with predefined smoothness assumptions, which enhances the representation ability of models."
- the fourth paragraph of the "Deep active learning approach" Section of the revised manuscript: "To fully leverage the experimental throughput of parallel evaluations, an acquisition function of Thompson Sampling (see **Methods**) is then constructed based on the posterior distribution from the surrogate model of DKL. This strategy enables scalable batch-parallel optimization and efficiently sample the parameter space of electrolyte formulas by balancing exploration (i.e., testing regions with high uncertainty) and exploitation (i.e., testing promising regions based on completed experiment results) [50]"

4.Overall, I think this work is highly interdisciplinary. Researchers from the battery field and the machine learning field could easily become confused. Therefore, integrating the two effectively will be a major challenge for the authors to address in order to improve the work.

Our response: We thank the reviewer for this suggestion. To ensure clarity for readers from both battery and machine learning fields, we have revised the Abstract, Introduction, and Conclusion sections to better highlight the fundamental motivation, the key technical challenges, and our corresponding strategies. For clarity and readability, these revisions are marked in red in the revised manuscript.

Reviewer #3 (Remarks on code availability):

The explanation of the code is not very well incorporated. I think the code needs to be commented better for broader audience.

Our response: Thank you for your comment. In the revised version, we have further improved the code documentation and explanation to make it more accessible for a broader audience. All source codes have been uploaded to our public GitHub repository (https://github.com/XWang1999/Electrolyte_Optimization_Deep_Active_Learning), which

now includes detailed comments, structured docstrings, and comprehensive documentation specifying the Python library dependencies and step-by-step instructions for reproducing the optimization workflow. Specifically:

- The main demonstration notebook (*Electrolyte_Optimization_Demo_DAL.ipynb*) has been enriched with additional explanations and comments for each active learning iteration, providing an executable example of how the method operates in practice.
- The Deep Kernel Learning (DKL) implementation is provided in the *dknet* folder, which details the construction and training of the hybrid neural network–Gaussian process surrogate. In addition, a numerical example implemented in *DAL_numerical_demo.py* illustrates the optimization process on a benchmark test function, offering a clear demonstration of how the DKL-based surrogate performs iterative learning and guides the search toward optimal regions.
- For optimization in expanded parameter spaces, the framework can be directly extended by modifying the Target Statistic Coding (TSC) module according to the annotations and explanations provided in the notebook. This procedure enables knowledge transfer between tasks and has been explicitly described in both the manuscript and the released code, ensuring that the workflow can be readily adapted for broader electrolyte design and related optimization scenarios.

These improvements enhance the explanation of the code ensuring that researchers from different backgrounds can readily understand and apply the workflow.

Response Letter to Reviewers' Comments on Manuscript
NCOMMS-25-60533A

We thank the reviewers for their valuable comments. In response, we have carefully addressed each point and incorporated the suggested changes into the revised manuscript (major changes are highlighted in red for clarity), as detailed below. Answers to the reviewers' comments are presented in blue, and the corresponding revised portions of the manuscript are copied here and marked in red.

REVIEWER COMMENTS

Reviewer #1 (Remarks to the Author):

The manuscript has been revised pretty well according to the 1st round review. All my concerns have been well addressed and I believe this work can cause wide interests and broad impact.
Our response: We thank the reviewer for the positive assessment and are grateful for the helpful comments from the first round, which have significantly strengthened the manuscript.

Reviewer #2 (Remarks to the Author):

The authors have provided a detailed response to my comments in the first round of revision and many of my comments have been satisfactorily addressed, including the release of codes and datasets. However, some critical comments remain unaddressed. See below:
1. Comment #2 in the first round on predicting aleatoric uncertainties has not been taken care of but can be addressed. I recommend the authors to follow this reference for guidance: Zhong et al., Nat. Commun. 16, 4522 (2025). Predicting aleatoric uncertainties is critical for quantifying reproducibility in the experimental measurements.
Our response: We thank the reviewer for emphasizing the importance of explicitly modeling aleatoric uncertainty and for pointing us to the recent work by Zhong *et al.* (*Nat. Commun.* 16, 4522, 2025). Disentangling aleatoric and epistemic uncertainties is valuable for assessing experimental reproducibility and robustness. From an algorithmic perspective, Zhong *et al.* decompose predictive uncertainty by sampling from the posterior distribution of network weights, defining the aleatoric component as the expected predictive entropy under fixed model parameters and the epistemic component as the residual uncertainty induced by variability across weight samples. In contrast, our framework employs a Gaussian process with deep kernel learning under a homoscedastic noise assumption, in which observation noise is treated as input-independent. As a result, while the model provides well-calibrated predictive uncertainty that primarily reflects **epistemic** uncertainty arising from sparse sampling and limited coverage of the compositional design space, it does not explicitly parameterize input-dependent aleatoric uncertainty. Consequently, a formal decomposition of predictive uncertainty into aleatoric and epistemic components is not directly supported within the current modeling formulation.

Importantly, this modeling choice is aligned with the primary objective of our study and is also consistent with the conclusions reported by Zhong *et al.* (*Nat. Commun.* 16, 4522, 2025). In the small-sample experimental regime considered here, uncertainty estimates are used to guide exploration-exploitation trade-offs under a limited experimental budget, where epistemic uncertainty dominates and relative uncertainty rankings are sufficient for identifying informative

regions of the search space. Consistent with the findings of Zhong *et al.*, in early-stage active learning with limited data, uncertainty decomposition provides limited practical benefit due to unstable epistemic estimates, and acquisition strategies based on overall predictive uncertainty are more robust and data-efficient, even in the absence of an explicit aleatoric-epistemic separation.

We agree with the reviewer that extending the present framework to explicitly model input-dependent aleatoric uncertainty represents an important direction for future work. While the current DKL-GP formulation adopts a homoscedastic noise assumption, future work will consider heteroscedastic Gaussian process models to explicitly capture input-dependent experimental noise, thereby improving uncertainty decomposition and reproducibility-oriented analyses. We have now explicitly acknowledged this limitation and the corresponding future research direction in the **Discussion section** of the revised manuscript.

2. Comparison of t-SNE plots of all electrolytes (suggested ones should be clearly highlighted) from different batches in point #7 in the first round are important. The authors can refer to this reference for guidance: Khalak et al., J. Chem. Theory Comput. 18, 6259-6270 (2022) (Figure 5).

Our response: We thank the reviewer for this suggestion. Low-dimensional visualizations are powerful for illustrating how an algorithm explores a complex design space. In Khalak *et al.* (*J. Chem. Theory Comput.* 18, 6259-6270 (2022)), t-SNE is applied to a **molecular encoding space**, where each molecule is represented by a high-dimensional descriptor vector. In that context, the high dimensionality arises primarily from the choice of molecular descriptors and encoding scheme, and t-SNE is well suited to reveal latent structure in this continuous, descriptor-based chemical space.

In our work, however, the underlying design problem is fundamentally different. We do not optimize over individual molecular structures, but over electrolyte formulations, which are defined by a finite set of discrete components (salt, solvent, additive) and their concentrations. Here, the increase in dimensionality is driven by the combinatorial diversity of component types and mixing ratios, rather than by a continuous, high-dimensional molecular descriptor space. Encoding each formulation into a high-dimensional vector (e.g., via one-hot representations of component identities) and then applying t-SNE would mainly reflect the arbitrary categorical encoding of the formulation space, rather than a physically meaningful notion of molecular similarity. As a result, the apparent "clusters" in such a t-SNE map would largely be determined by how the categories are encoded, and would not necessarily provide additional mechanistic insight into the electrolyte behavior.

For this reason, instead of using a t-SNE embedding of a formulation encoding, we adopted a component-resolved visualization strategy that is more directly aligned with how electrolyte formulations are designed and interpreted. In the revised manuscript, we explicitly analyze how DAL reshapes the exploration of the design space by tracking the evolution of: salt identity (**Fig. 3c**), solvent identity (**Supplementary Fig. 6e**), salt concentration (**Supplementary Fig. 21a**), and additive identity (**Supplementary Fig. 22a**) across different DAL iterations.

These plots show, for example, that DAL persistently and selectively explores formulations containing LiTFSI and LiFSI, while deprioritizing LiDFOB-rich regions after the first iteration due to their high experimental uncertainty and limited performance gains. This component-wise view makes it transparent which parts of the formulation space are being exploited and which are being discarded, and it directly reflects the way experimentalists reason about multi-component

electrolytes.

We have clarified this rationale and strengthened the discussion in the main text. The corresponding paragraph now reads: "We now further show the success of DAL from a microscopic scale. **Figure 3c** shows the evolution of the space that is grouped by the type of salts (as a representative). The electrolyte formulas for the first round are randomly selected with two replicate tests (first column in **Fig. 3c**) to generate an initial dataset for DAL model building. Through only one DAL iteration, some electrolytes (i.e., those containing LiTFSI and LiFSI) are identified as "potential candidates" (second column in **Fig. 3c**). Interestingly, the LiDFOB regions (red dot area, first column in **Fig. 3c**) were allocated with many resources for the first iteration, but much fewer after that, which indicates the high experimental uncertainty in this zone that requires careful validation. By learning and exploiting structural information of the parameter space, DAL avoids evaluating electrolytes with low estimated cell cycle life and directs more resources to the high-performance region. This is also confirmed by individually analyzing other components of the space (discussed in **Supplementary Fig. 6**)."

We hope that this formulation-centric and component-resolved visualization adequately addresses the reviewer's request for a clear depiction of how DAL navigates and concentrates the exploration within the electrolyte design space.

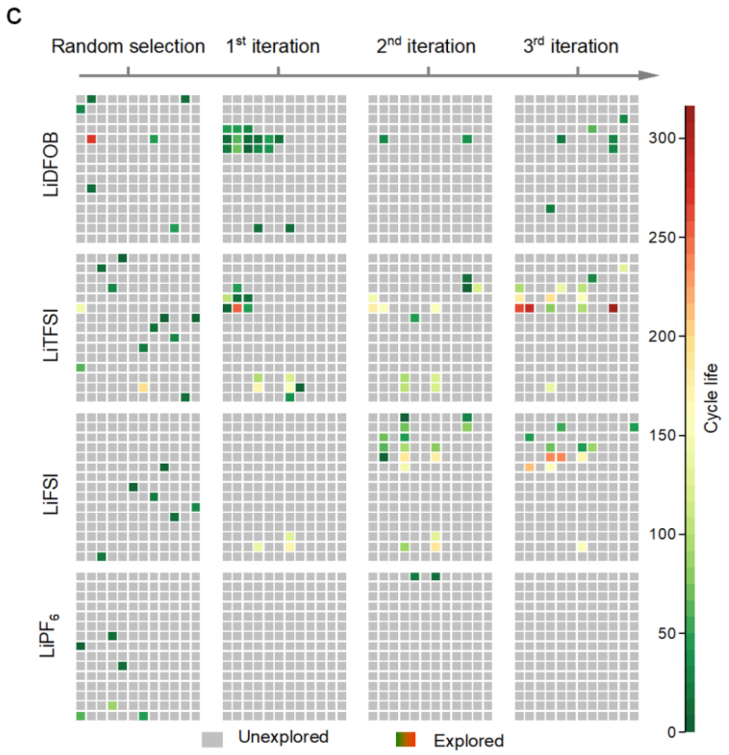

Fig. 3c, The visualization of the DAL iterations showing the evolution of distribution of tested electrolytes and its battery cycle life (color coded) as iteration progress, which is herein representatively grouped by the type of lithium salts for easy visualization.

3. The authors' reasons for not using any chemical representation in the present framework is not justified (point #8 in the first round). If data-scarcity was the issue then one can use a pretrained model to generate molecular features for solvents, salts, and additives and feed to the surrogate

model. The current approach may not lead to any new molecular design of electrolytes in a zero-shot manner, which stands in contrast to their claims of "optimization over discrete electrolyte space".

Our response: We thank the reviewer for this point and for highlighting the possibility of using pretrained molecular representations. The molecular foundation models are becoming central to de novo molecular design, and pretrained encoders can provide rich molecular features for solvents, salts, and additives.

Our goal in this work, however, is more modest and differs in scope from de novo, zero-shot molecular design. We focus on **data-efficient optimization of electrolyte formulations** for long-term cycle life within a **fixed, experimentally accessible library** of salts, solvents, and additives, under a specific cycling protocol. Each labeled data point corresponds to a full-cell life test that typically requires months to complete and is strongly influenced by electrodes, cell geometry, formation procedures, and test conditions. Consequently, the number of reliably labeled formulations in our study is orders of magnitude smaller than the 10^4–10^6 molecules or mixtures typically used to train or fine-tune current chemical foundation models, which are often built on MD-derived structural descriptors, transport properties, or equilibrium thermodynamic quantities.

In principle, one could take an existing pretrained molecular encoder and use it to generate fixed embeddings for the solvents, salts, and additives in our library, and then feed these embeddings into the DKL surrogate model. In our setting, however, the main challenge is **not only data scarcity,** but also **task and domain misalignment** between available pretrained models and our target property. Existing electrolyte-related foundation models are generally pretrained on simulated or literature-derived data that emphasize equilibrium or short-timescale properties (e.g., solvation structure, ionic conductivity, viscosity, density). By contrast, the cycle life of a full cell over hundreds of cycles is governed by long-timescale interfacial degradation, parasitic reactions, gas evolution, and coupled electrode-electrolyte failure mechanisms, which are only indirectly reflected in those pretraining tasks. In a small-data regime such as ours, adding high-dimensional molecular embeddings that are not well aligned with the target property can in fact make the surrogate harder to train and less stable, without guaranteeing a corresponding gain in predictive power.

Importantly, our current framework does not omit chemical information; rather, it adopts a formulation-centric chemical representation that is tightly coupled to the experimental objective. Instead of representing individual molecules in a continuous descriptor space, we encode experimentally derived interaction descriptors such as the ADI for pairs and higher-order combinations of components. These features summarize, in a low-dimensional and task-specific way, how particular combinations of salts, solvents, and additives affect the observed cycle life across all available formulations. As new experiments are performed, these interaction descriptors are updated, providing an end-to-end, experiment-driven representation that directly reflects the "composition-structure-performance" relationship relevant to long-term cycling stability.

This design has two practical consequences. First, it enables combinatorial optimization over a large discrete formulation space spanned by a fixed library of experimentally accessible components, which is the actual focus of this work. Second, it naturally supports few-shot expansion of the space: when a new component is introduced, only a small number of targeted experiments are needed to estimate its interaction descriptors with existing components, after which it can be seamlessly integrated into the optimization loop without retraining a separate molecular encoder.

To avoid overstating our current capabilities, we have revised the wording of our claims in the

Abstract and Discussion. Specifically, we replace "optimization over discrete electrolyte space" with "optimization of formulations within a fixed discrete component library". We also add a short paragraph in the Discussion outlining how pretrained molecular representation models could be integrated into our framework in future work, once sufficiently large and task-aligned pretraining datasets for long-term cycling performance become available.

4. When I asked to include a "Discussion" section, the goal was for the authors to clearly highlight the limitations. Currently, it is very brief and looks very generic and none of the main issues of their approach, which they themselves accept in responses to points #2, #8, and #9 from the first round, have been explicitly highlighted in the manuscript. It is important to highlight all the limitations of their approach such that the reader is better informed and future.

Our response: We thank the reviewer for this comment. Following the suggestions in both the first and second rounds, we have substantially revised the **Discussion and outlook** section to explicitly highlight the main limitations of our framework, especially those related to points #2, #8, and #9.

Specifically, we now:

1. **Clarify the scope of optimization and the lack of zero-shot molecular design (related to points #8 and #9).** We explicitly state that the current framework optimizes over combinatorial electrolyte formulations built from a fixed, experimentally accessible component library, and does not perform de novo, zero-shot molecular design. New solvents, salts, or additives still require targeted experiments before they can be incorporated into the optimization.
2. **Highlight the limitations of our formulation-centric representation and the TSC/ADI scheme** (related to points #8 and #9). We emphasize that chemical information is encoded via interaction statistics (e.g., ADIs) rather than detailed molecule-level embeddings, and that TSC and ADI are post-hoc abstractions. This design is pragmatic for small, noisy datasets but limits our ability to capture fine-grained structure–property relationships and implies a few-shot, rather than true zero-shot, capability for new components.
3. **Explicitly acknowledge limitations in uncertainty modeling (**related to point #2**).** We clarify that the current framework models uncertainty under a homoscedastic noise assumption, and therefore does not explicitly disentangle or quantify input-dependent aleatoric uncertainty. While this choice is appropriate for small-sample active learning where epistemic uncertainty dominates, extending the framework to heteroscedastic uncertainty modeling is considered as an important direction for future work and is now explicitly discussed.

These revisions are now summarized in the *Discussion and outlook* section of the revised manuscript: "In this work, we show that a DAL framework, built around a DKL surrogate and experimentally derived interaction descriptors, can accelerate the discovery of long-lifespan electrolyte formulations by steering experiments toward promising regions of a combinatorial formulation space. This implementation, however, is currently restricted to a fixed, experimentally accessible component library and to the specific cell chemistry and cycling protocol studied here; it does not perform de novo, zero-shot molecular design, but instead enables data-efficient optimization and few-shot expansion, for which newly introduced components still require targeted experiments. Chemical information is encoded in a formulation-centric manner through interaction statistics (such as ADIs), and TSC/ADI act as post hoc abstractions rather than learned molecular

embeddings, which is pragmatic for small, noisy datasets but limits the resolution of structure-property relationships and generalization across chemically distant components. In addition, the current framework models predictive uncertainty under a homoscedastic noise assumption, such that uncertainty estimates primarily reflect epistemic uncertainty arising from sparse sampling and limited coverage of the formulation space. As a result, input-dependent aleatoric uncertainty is not explicitly quantified, which constrains the assessment of experimental variability and reproducibility across heterogeneous conditions. Looking ahead, as larger, task-aligned electrolyte datasets available, molecular foundation models and pretrained mixture-level encoders could be integrated with these interaction-based features to improve generalization, while heteroscedastic uncertainty modeling could further enhance the characterization of experimental variability and reproducibility, all while preserving data efficiency and enabling a gradual transition from formulation-level optimization toward more genuine molecular design.

Reviewer #3 (Remarks to the Author):

I appreciate the response of the authors to my comments. I think the questions are overall very well presented. However, the answer to my Comment #4 is vague. I cannot find the details about the improvements completed by the author. The improvements to enhanced the significant of this work to the audience is highly essential. I hope this point can be addressed further.

Our response: We thank the reviewer for the follow-up comment, and we apologize that our previous response did not clearly describe the corresponding revisions. In the revised manuscript, we have implemented several concrete changes to make the interdisciplinary significance of this work more explicit for both battery and machine-learning audiences:

1. **Abstract (last two sentences).** We have rewritten the final two sentences of the Abstract to clearly state what the framework offers to each community. This change is intended to explicitly communicate the relevance of our framework to both readerships at the very beginning of the paper. The Abstract now ends with: "This work provides an experiment-driven, sample-efficient route to explore complex electrolyte formulation spaces and quantify inter-component correlations, as well as a realistic, high-cost, small-data benchmark for probabilistic surrogate modeling and sequential decision-making in discrete chemical spaces. Together, these advances establish an AI-assisted discovery framework for rapid exploration, knowledge learning and transfer in complex materials spaces, without relying on detailed mechanistic models or empirical design rules."
2. **Introduction (new "bridge" sentence).** At the end of the Introduction, we have added an explicit bridging sentence that summarizes what each community can take away from the DAL–TSC workflow:

   "From the perspective of battery research, this framework organizes costly cycling experiments into a structured, sample-efficient exploration of electrolyte formulations; from the perspective of machine learning, it provides a realistic, high-cost and small-data testbed for probabilistic surrogate modeling and sequential decision-making in discrete chemical spaces."

   This sentence is meant to guide battery researchers in understanding how the method translates into practical experimental strategies, and to help machine-learning researchers view the work as a well-defined benchmark problem rather than a black-box application.
3. **Discussion and outlook (explicit limitations and future directions).** We have substantially revised the **Discussion and outlook** section to make the limitations and future needs of this cross-disciplinary approach explicit for both fields. In particular, we now emphasize that:

   (i) the present DAL–DKL implementation is restricted to a fixed, experimentally accessible component library and a specific cell chemistry and protocol, and thus does not perform de novo, zero-shot molecular design;

   (ii) chemical information is encoded in a formulation-centric manner through interaction statistics (such as ADIs), with TSC/ADI acting as post-hoc abstractions rather than learned molecular embeddings, which is pragmatic for small, noisy datasets but limits structure–property resolution and generalization across chemically distant components; and

   (iii) the workflow still relies on specific design choices (component library, network

architecture, acquisition strategy and noise treatment) that may require adaptation in other chemistries or testing protocols.

We also explicitly outline future research directions on both sides: the need for larger, task-aligned electrolyte datasets and more physically grounded electrolyte models from the battery perspective, and the opportunity to integrate molecular foundation models and pretrained mixture-level encoders with our interaction-based features from the machine-learning perspective, in order to move from formulation-level optimization toward more genuine molecular design.

We hope that these targeted revisions clarify both the significance and the current limitations of our interdisciplinary framework for readers from the battery and machine-learning communities.
